# Diastereoselective 1,3-nitrooxygenation of bicyclo[1.1.0]butanes†

**DOI:** 10.1039/d4sc08753d

**Published:** 2025-03-25

**Authors:** Anirban Maity, Kuruva Balanna, Constantin G. Daniliuc, Armido Studer

**Affiliations:** a Organisch-Chemisches Institut, Universität Münster Corrensstraße 40 48149 Münster Germany studer@uni-muenster.de

## Abstract

Cyclobutanes are strained carbocycles found in many drugs and natural products. Herein, we report a diastereoselective 1,3-nitrooxygenation of bicyclo[1.1.0]butanes with *tert*-butylnitrite and TEMPO to access 1,1,3-trisubstituted cyclobutanes. Various bicyclo[1.1.0]butanes effectively participated in the radical reaction yielding the substituted cyclobutane scaffolds with excellent yields and diastereoselectivity. The reaction is catalyst-free, easy to perform, and scalable and can be conducted in open air. Products obtained serve as substrates for the synthesis of 1,1,3,3-tetrasubstituted cyclobutanes with good yields and diastereoselectivity.

## Abstract 2

Diastereoselective 1,3-nitrooxygenation of bicyclo[1.1.0]butanes with *tert*-butylnitrite and TEMPO to access 1,1,3-trisubstituted cyclobutanes is reported.

## Graphical abstract

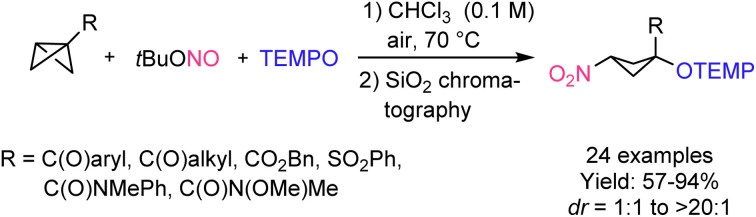

## Introduction

**Scheme 1:**
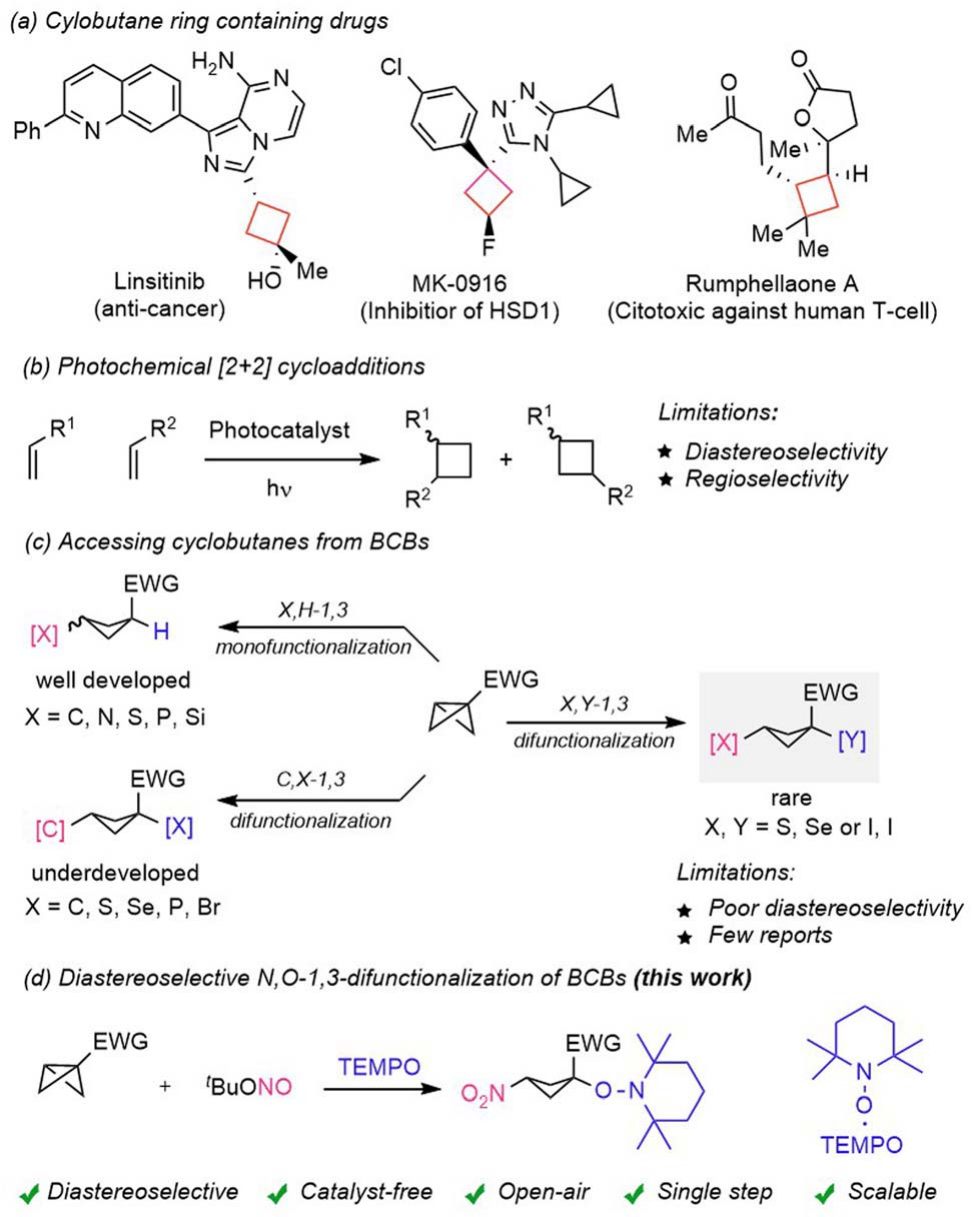
(a) Cyclobutane ring containing drugs. (b) Photochemical [2 + 2] cycloaddition. (c) Accessing cyclobutanes from BCBs. (d) This work: diastereoselective *N*,*O*-1,3-difunctionalization of BCBs.

Cyclobutanes are important structural units found in many naturally occurring and pharmaceutically active bio-molecules (Scheme 1a).^1^ In this regard, nitrocyclobutanes^1*e*,*f*^ are highly valuable intermediates in organic synthesis^1*g*^ due to their unique dual reactivity, arising from both the strained cyclobutane core and the electron-withdrawing nitro group. The nitro functionality serves as a versatile handle for various transformations, including reductions to amines, Henry reactions, and Nef reactions, which facilitate the formation of ketones and other functional groups. More specifically, highly functionalized and well-decorated cyclobutanes are often challenging to synthesize in a straightforward manner. Major synthetic routes for the synthesis of these strained carbocycles include radical cyclization,^2^ Wolff-rearrangement,^3^ oxidative pinacol-rearrangement,^4^ and the recently reported nitrogen-deletion strategy.^5^ Photocatalyst mediated intermolecular [2 + 2] cycloaddition^6^ has been extensively used for the construction of such strained carbocycles (Scheme 1b). However, despite significant advancement in energy transfer mediated photochemical [2 + 2] cycloadditions the outcome of these reactions is often complicated because of regio- and diastereoselectivity issues,^6^ and careful selection of π-systems is often a prerequisite criterion to achieve the desired selectivity. In recent years an additional approach has emerged, which involves the exploitation of strained bicyclo[1.1.0]butanes (BCBs)^7^ to access a variety of complex cyclobutane scaffolds (Scheme 1c). Due to the 96% p-character of the central C–C bond of a BCB, such compounds show a diverse range of reactivities varying from nucleophilic, electrophilic and radicalophilic.^7^ The pioneering work of nucleophilic additions to BCBs was realized by Gaoni *et al.*^8^

Moreover, the Baran group^9^ developed amination of BCBs using amines as nucleophiles. Biju *et al.*^10^ and Feng, Qu, Yu *et al.*^11^ independently developed diastereoselective arylation of disubstituted-BCBs. Along with nucleophilic ring-openings, several radical additions^1,12^ to BCBs were reported, predominantly for mono-functionalization. Considering difunctionalizing ring-opening of BCBs, except for few cases, the majority of these transformations suffer from low diastereoselectivity. Along these lines, diastereoisomerically enriched 1,1,3-trisubstituted cyclobutanes were successfully prepared by the Fox group^13^ through organocuprate addition and subsequent trapping using an external electrophile. The Glorius group^14^ developed a 1,3-carbothiolation of BCBs through an intramolecular group transfer process to secure high *syn*-selectivity. The Hari group^15^ presented a 1,3-carboheterodifunctionalization of BCBs for the synthesis of spirocyclobutyl lactones and lactams, albeit with moderate diastereocontrol. Boronate-BCBs were investigated by the Aggarwal group^16^ who achieved highly diastereoselective 1,3-carbodifunctionalization by employing various electrophiles or electrophilic radicals to induce the ring-opening process. The preinstalled boronate functionality controls both regio- and diastereoselectivity of the products, 1,1,3-trisubstituted cyclobutanes.

In addition to mono and 1,3-difunctionalization, cycloaddition^17^ and insertion reactions^1^ of BCBs have offered synthetic chemists direct and straightforward routes to access cyclobutane rings containing complex 3D-enriched scaffolds. However, only carbon–carbon (C–C) and carbon–heteroatom (C–S) 1,3-difunctionalization was presented in a diastereoselective manner to date. The diastereoselective heteroatom–heteroatom (X,Y) 1,3-difunctionalization of bicyclo[1.1.0]butanes is rare.^18^ In order to achieve a heteroatom–heteroatom, particularly (O–N) 1,3-difunctionalization, we considered using *tert*-butyl nitrite (^*t*^BuONO)^19^ as a nitrogen atom source in combination with the TEMPO radical^20^ as an oxygen atom source (Scheme 1d). ^*t*^BuONO upon thermal homolysis will generate a persistent NO-radical, which in the presence of molecular oxygen will be oxidized to a transient NO_2_-radical. The NO_2_-radical should then add along the central C–C bond of the BCB to generate a transient adduct cyclobutyl radical, which can engage in a selective cross-coupling with the persistent TEMPO radical to afford the desired 1,3-difunctionalized cyclobutane product. Interestingly, in this reaction two persistent radicals (NO and TEMPO) are present and the persistent NO-radical is selectively converted to a transient NO_2_-radical during the course of the reaction, while TEMPO remains unchanged. The suggested difunctionalizing BCB ring-opening reaction presents challenges that are product stability^21^ and diastereoselectivity control, as radical/radical cross couplings generally occur with very low barriers.

## Results and discussion

Readily prepared (see the ESI†) bicyclo[1.1.0]butan-1-yl(naphthalen-2-yl)methanone (1) was chosen as the model substrate. We were pleased to observe that the reaction of 1 with ^*t*^BuONO (0.20 mmol, 2.0 equiv.) and TEMPO (0.15 mmol, 1.5 equiv.) in DMSO at 70 °C for 18 hours afforded the desired 1,3-nitrooxygenated product 2 in 38% NMR-yield, albeit with a moderate 2.2 : 1 diastereoselectivity (Table 1, entry 1). Then, different solvents were tested under otherwise identical conditions. In CHCl_3_, yield of the product 2 increased to 82% but diastereoselectivity further decreased (Table 1, entry 2). Other solvents such as DCE (76%), toluene (48%), 1,4-dioxane (64%), and CH_3_CN (74%) afforded lower yields and lower diastereoselectivities (Table 1, entries 3–6). The lower efficiency of the transformation in toluene and 1,4-dioxane can be rationalized by considering the competitive hydrogen atom transfer (HAT) reaction with the reactive NO_2_-radical from the solvent.

**Table 1:** Reaction optimization

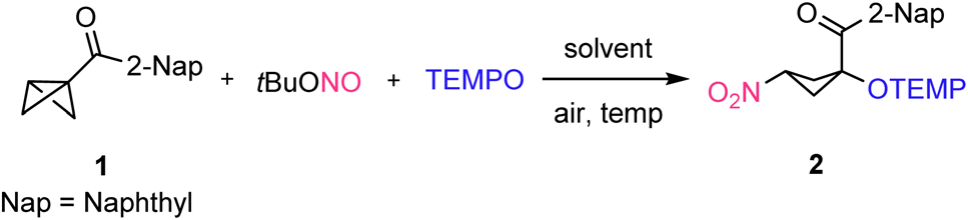				
#	Solvent	Temperature	dr	Yield^a^^,^^b^ (%)
1	DMSO	70 °C	2.2 : 1	38
**2**	**CHCl**_**3**_	**70 °C**	**1.4 : 1**	**82**
3	DCE	70 °C	1.4 : 1	76
4	Toluene	70 °C	1.2 : 1	48
5	1,4-Dioxane	70 °C	1.1 : 1	64
6	CH_3_CN	70 °C	1.0 : 1	74
7	CHCl_3_	25 °C	1.7 : 1	63
8	CHCl_3_	100 °C	3.9 : 1	81
9^c^	CHCl_3_ (DMAP)	70 °C	4.4 : 1	82
10^c^	CHCl_3_ (DBU)	70 °C	5.8 : 1	61
11^c^	CHCl_3_ (Cs_2_CO_3_)	70 °C	5.3 : 1	81
**12**	**CHCl**_**3**_	**70 °C**	**>20 : 1**	**74^d^**

a Reaction conditions: 1 (0.1 mmol), ^*t*^BuONO (0.20 mmol), TEMPO (0.15 mmol), solvent (0.1 M), air, 18 h.

b Determined by ^1^H NMR using 1,3,5-trimethoxy benzene as an internal standard.

c After removal of solvent the crude was re-dissolved in CHCl_3_ (0.1 M) in the presence of the given base (20 mol%) and stirred for 18 h at rt.

d Isolated yield and diastereomeric ratio after silica gel column chromatography of the reaction mixture.

The optimization campaign was continued by varying reaction temperature using CHCl_3_ as the solvent. At room temperature a significantly lower yield (63%) resulted, possibly due to the slow decomposition of ^*t*^BuONO (Table 1, entry 7). Upon increasing temperature from 70 °C to 100 °C the yield of the reaction remained unchanged (81%); however, an improved diastereoselectivity was noted. This surprising stereochemical outcome indicated that isomerization likely occurred after initial BCB-nitrooxygenation, probably through deprotonation/re-protonation of the acidic proton next to the nitro group. Despite the fact that reversible alkoxyamine C–O-bond homolysis in 2 could not be excluded as the isomerization process at that temperature, we tested whether the poor diastereomeric ratio can be improved by applying a deprotonation/re-protonation strategy through simple addition of a base. To this end, several bases were tested as catalysts for the isomerization of 2. After radical nitrooxygenation, the solvent was removed and the crude nitro-cyclobutane 2 was then re-dissolved in CHCl_3_ in the presence of DMAP (20 mol%) and stirred for 18 h at room temperature. The diastereoselectivity improved without compromising the yield (Table 1, entry 9, 82%, dr 4.4 : 1). With DBU (20 mol%) as the base, the diastereomeric ratio further improved, albeit at the expense of the NMR yield (Table 1, entry 10, 61%, dr 5.8 : 1). In the presence of Cs_2_CO_3_ (20 mol%), product 2 was obtained with a diastereomeric ratio of 5.3 : 1 without scarifying the yield (Table 1, entry 11). Pleasingly, upon purification of the crude product 2 through silica gel column chromatography without any prior base treatment, we found complete isomerization and 2 (*syn*-isomer) was isolated in 74% yield with an excellent diastereomeric ratio (dr > 20 : 1, Table 1, entry 12). The relative configuration was unambiguously assigned by X-ray analysis and other compounds prepared in this study were assigned in analogy. Obviously, the additional isomerization step using an external base is not required, as isomerization to the thermodynamic *syn*-product efficiently occurs during silica gel chromatography. With these optimized conditions in hand, we then investigated the scope of the reaction.

**Scheme 2:**
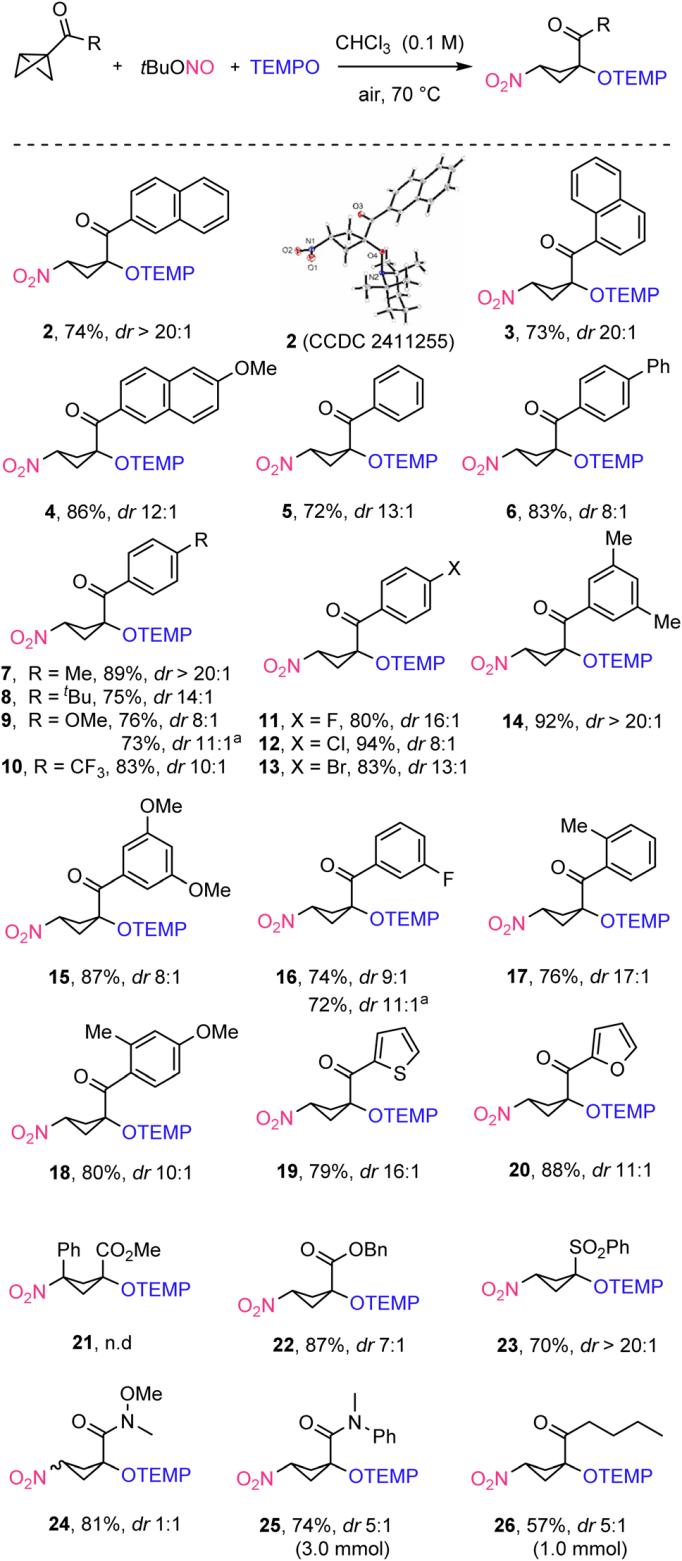
Reaction conditions: bicyclo[1.1.0]butane (0.2 mmol, 1.0 equiv.), ^*t*^BuONO (0.4 mmol, 2.0 equiv.), TEMPO (0.3 mmol, 1.5 equiv.), CHCl_3_ (2.0 mL). Yields and the diastereomeric ratio are reported after isolation *via* column chromatography. ^*a*^A second column chromatography was performed.

First, the aryl group of the keto BCB was varied. 1-Naphthyl and 6-methoxy-substituted 2-naphthyl-keto-BCBs afforded the desired products 3 and 4 in 73% and 86% yield with high diastereoselectivity (dr 20 : 1 and 12 : 1) (Scheme 2). The unsubstituted phenyl keto-BCB provided the desired cyclobutane 5 in 72% yield and high stereoselectivity (dr 13 : 1). Aryl keto-BCBs carrying an electron donating or withdrawing group at the aryl ring were eligible substrates. Thus, BCBs carrying an aryl group or an alkyl group at the 4-position of the aryl substituent such as 4-phenyl, 4-methyl, or 4-*tert*-butyl, afforded the desired products 6–8 with good to excellent yields (75–89%) and good to excellent diastereoselectivity (dr 8 : 1 to > 20 : 1). The reason for the varying selectivity as a function of the *para*-substituent is not fully understood. Along the same lines, electron-rich 4-MeOC_6_H_4_ and electron-poor 4-CF_3_C_6_H_4_-substituted BCBs afforded the desired products 9 and 10 in 76% and 83% yield, with good diastereoselectivity (dr 8 : 1 and 10 : 1). Renewed chromatography of 9 led to a slightly improved diastereoselectivity (11 : 1 *versus* 8 : 1). 4-Halo-substituted aryl keto-BCBs delivered the targeted 1,3-nitrooxygenated products 11–13 in good to excellent yields (80–94%) and good diastereoselectivity (dr 8 : 1 to 16 : 1). Installing a substituent at the *meta* position of the aryl ring such as in 3,5-dimethyl-C_6_H_3_, 3,5-dimethoxy-C_6_H_3_, and 3-fluoro-C_6_H_4_-substituted keto-BCBs afforded the desired products 14–16 in good to excellent yields (74–92%) and good to excellent diastereoselectivity (dr 8 : 1 to dr > 20 : 1). *ortho*-Methyl substituted phenyl keto-BCB delivered the 1,3-nitrooxygenated product 17 in 76% yield with high diastereoselectivity (dr 17 : 1). A 2,4-disubstituted phenyl keto-BCB worked well to afford 18 in 80% yield with good diastereoselectivity (dr 10 : 1). Heterocyclic rings such as thiophene and furan are tolerated and the corresponding 1,3-nitrooxygenated products 19 and 20 were isolated in 79% and 88% yields with high stereoselectivity (dr 16 : 1 and 11 : 1). However, a 1,3-disubstituted-BCB did not afford the desired 1,3-nitrooxygenated product 21, possibly because of steric reasons: the 3-phenyl substituent in the BCB hinders NO_2_-radical attack and ring opening does not occur.

Moreover, phenylsulfonyl-activated BCBs also engage in the radical cascade reaction, as shown by the successful synthesis of sulfone 23 that was isolated in 70% yield with excellent diastereoselectivity (dr > 20 : 1). Notably, this methodology is not restricted to aromatic keto-BCBs. It is also effective for the ring-opening di-functionalization of ester-BCBs, amide-BCBs, and alkyl keto-BCBs, yielding trisubstituted cyclobutane derivatives with good to excellent yields and low to moderate diastereoselectivity (22, 87%, dr 7 : 1; 24, 81%, dr 1 : 1; 25, 74%, dr 5 : 1; 26, 57%, dr 5 : 1).

**Scheme 3:**
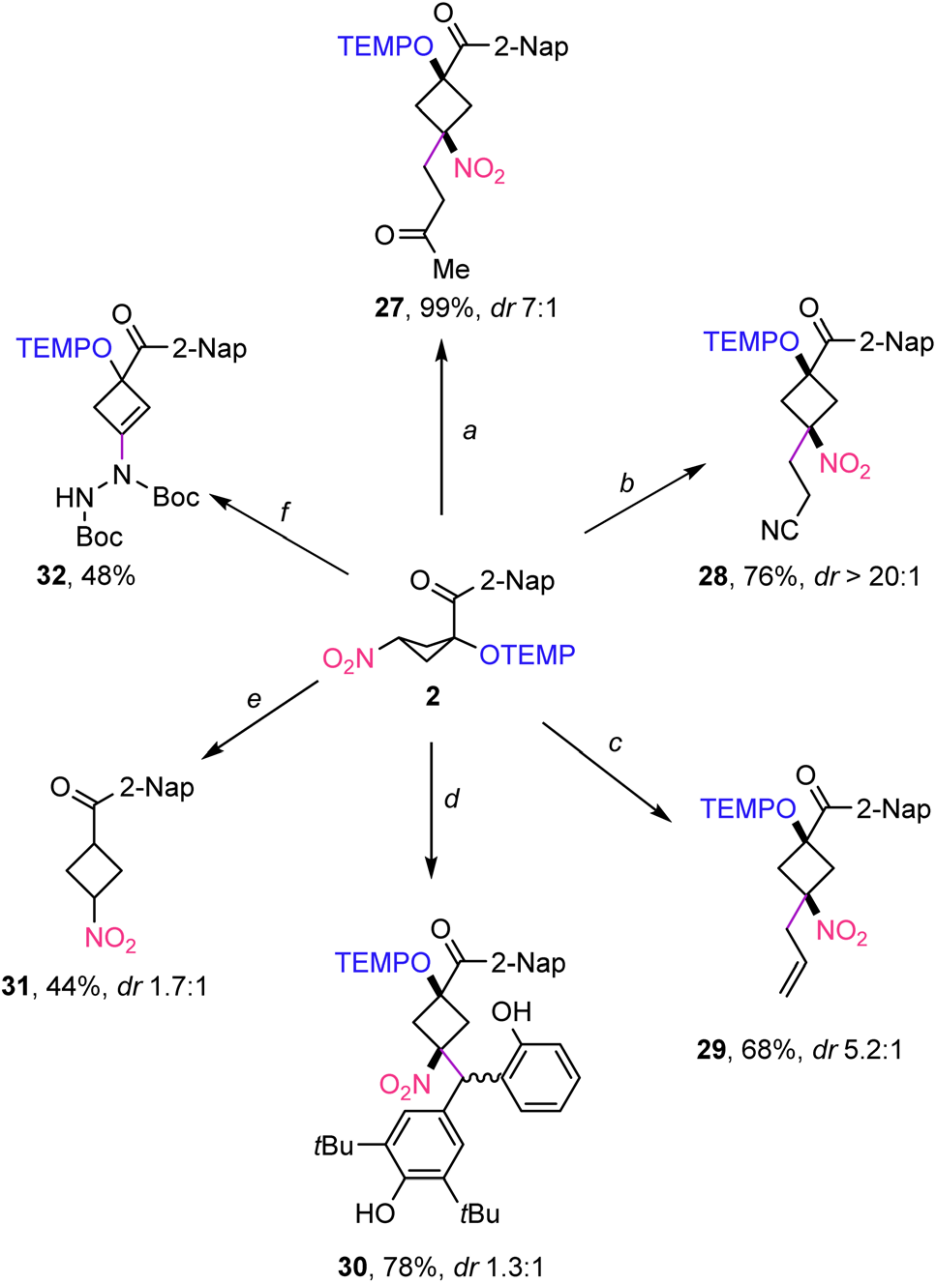
Follow-up chemistry. ^*a*^Reaction conditions: 2 (0.15 mmol, 1.0 equiv.), methyl vinyl ketone (0.18 mmol, 1.2 equiv.), tetramethyl guanidine (0.075 mmol, 0.5 equiv.), THF (0.9 mL). ^*b*^Reaction conditions: 2 (0.15 mmol, 1.0 equiv.), acrylonitrile (0.18 mmol, 1.2 equiv.), tetramethyl guanidine (0.075 mmol, 0.5 equiv.), THF (0.9 mL). ^*c*^Reaction conditions: 2 (0.2 mmol, 1.0 equiv.), allyl alcohol (0.6 mmol, 3.0 equiv.), Pd(PPh_3_)_4_ (0.02 mmol, 0.1 equiv.), DMSO (1.2 mL). ^*d*^Reaction conditions: 2 (0.1 mmol, 1.0 equiv.), 2,6-di-*tert*-butyl-4-(2-hydroxybenzylidene)cyclohexa-2,5-dien-1-one (0.1 mmol, 1 equiv.), Cs_2_CO_3_ (0.11 mmol, 1.1 equiv.), Bi(OTf)_3_ (0.02 mmol, 0.2 equiv.) DCE (1.0 mL). ^*e*^Reaction conditions: 2 (0.1 mmol, 1.0 equiv.), γ-terpene (0.3 mmol, 3.0 equiv.), *tert*-butanol (5.0 mL). ^*f*^Reaction conditions: 2 (0.10 mmol, 1.0 equiv.), di-*tert*-butyl (*E*)-diazene-1,2-dicarboxylate (0.15 mmol, 1.5 equiv.), Cs_2_CO_3_ (0.11 mmol, 1.1 equiv.), DCE (1.0 mL).

The synthetic value of the nitrocyclobutane products was documented by conducting several follow-up reactions (Scheme 3). Michael reaction of 2 with methyl acrylate and acrylonitrile afforded the 1,1,3,3-tetrasubstituted cyclobutanes 27 and 28 in 99% and 76% yields with good to excellent diastereoselectivity (dr 7 : 1 and dr > 20 : 1). Palladium catalysed allylation of 2 with allyl alcohol was achieved to afford 29 in 68% yield with moderate diastereoselectivity (dr 5.2 : 1). The relative configuration of the separable isomers was assigned by NOE experiments (see the ESI†). Selectivity of the other products derived from 2 was assigned in analogy. *para*-Quinomethide as an electrophilic coupling partner delivered cyclobutane 30 in 78% yield, albeit with poor diastereoselectivity. Thermal homolysis of the weak C–O bond of alkoxyamine 2 in the presence of γ-terpene as a hydrogen atom transfer (HAT)^22^ reagent afforded the 1,3-disubstituted cyclobutane 31 in 44% yield (88%, brsm) and moderate diastereoselectivity. Hydrazination of nitrocyclobutane 2 under basic conditions gave cyclobutene 32 in 48% isolated yield. Hence, the targeted nitrocyclobutane obtained after initial hydrazination eliminates HNO_2_ under the applied conditions.

**Scheme 4:**
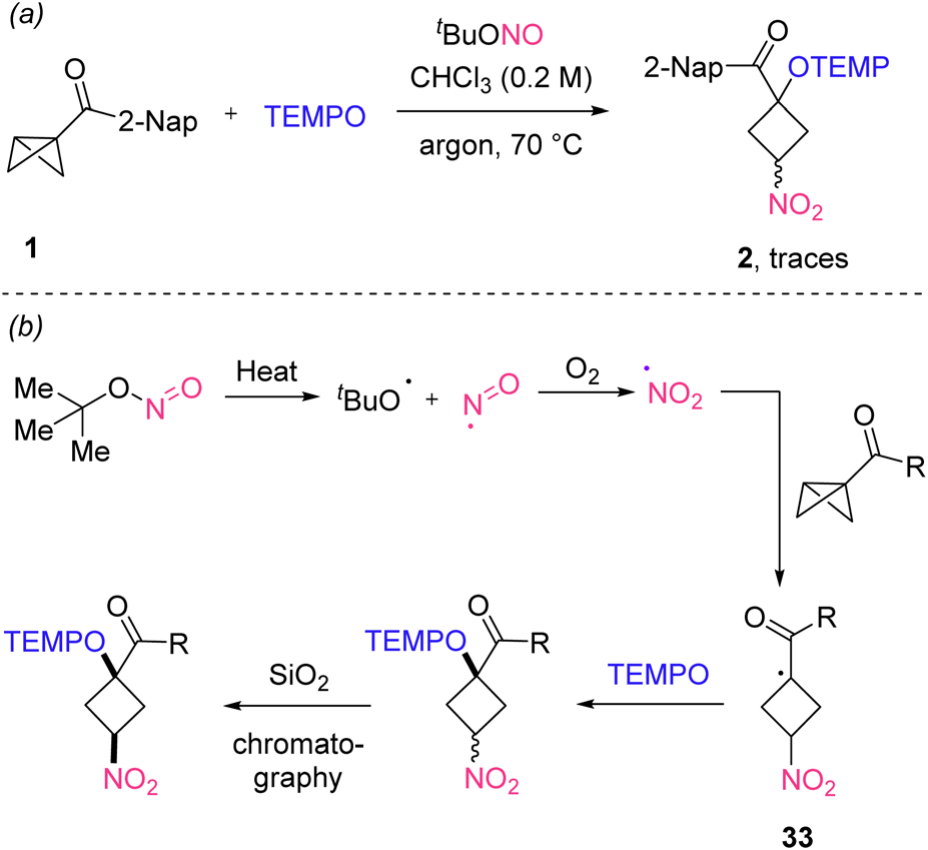
(a) Control experiment. (b) Plausible reaction mechanism.

In order to study the effect of O_2_, the reaction of 1 was repeated under an argon atmosphere and only traces of product 2 were detected (Scheme 4a). Based on this observation and literature reports^19^ we propose a plausible mechanism that is depicted in Scheme 4b. Thermal homolysis of the N–O bond in ^*t*^BuONO leads to the formation of the *tert*-butoxyl radical along with the persistent nitroso (NO) radical, which in the presence of molecular oxygen is directly oxidized to the NO_2_-radical. The NO_2_-radical then engages in a homolytic substitution at carbon cleaving the central C–C bond of the bicyclo[1.1.0]butane to generate the adduct radical 33. This adduct radical is then trapped by the persistent TEMPO radical^23^ to deliver the isolated product. Of note, the TEMPO-trapping does not occur with a high stereoselectivity and isomerization through a deprotonation/re-protonation sequence forming a thermodynamically more stable isomer during silica gel chromatography and ensures the very high diastereoselectivity observed in many of our transformations.

## Conclusions

In conclusion we have developed a highly diastereoselective 1,3-nitrooxygenation of bicyclo[1.1.0]butanes for the preparation of 1,1,3-trisubstituted cyclobutane scaffolds. It is shown that the radical ring-opening TEMPO-trapping occurs with low or moderate stereoselectivity. Nevertheless, due to the simple isomerization through deprotonation/re-protonation during silica gel chromatography, the thermodynamic *syn*-isomer can be obtained with good to excellent diastereoselectivity. The reported cascade worked for a wide variety of substrates, delivering the targeted nitrocyclobutane products in good to very good yields. Follow-up chemistry demonstrated that the ring-opening products formed through the radical cascade can be used as substrates for the synthesis of 1,1,3,3-tetrasubstituted cyclobutane scaffolds with good diastereocontrol.

## Data availability

Experimental procedures and analytical data (NMR, HRMS, IR, melting points) that support the findings of this study are available in the ESI.†

## Author contributions

A. M. and K. B. conducted all the experiments and characterized all novel compounds. C. G. D. measured and solved the X-ray crystal structure. A. M., K. B. and A. S. designed the experiments and wrote the manuscript.

## Conflicts of interest

There are no conflicts to declare.

## Supplementary Material

SC-OLF-D4SC08753D-s001

SC-OLF-D4SC08753D-s002
