# Supplementary material for: Diastereoselective 1,3-nitrooxygenation of bicyclo[1.1.0]butanes
Source: Chem Sci. 2025 Mar 25;16(17):7264–9. doi: 10.1039/d4sc08753d (PMC11956531; doi:10.1039/d4sc08753d)
Supplement: SC-016-D4SC08753D-s001 [file SC-016-D4SC08753D-s001.pdf]

Supplementary Information (SI) for Chemical Science.
This journal is © The Royal Society of Chemistry 2025

Supporting information

Diastereoselective 1,3-Nitrooxygenation of Bicyclo[1.1.0]butanes

*Anirban Maity, Kuruva Balanna, Constantin G. Daniliuc and Armido Studer**

1. General methods and material……………………………………………………………………….2

2. General procedure for 1,3-nitrooxygenation of bicyclo[1.1.0]butanes (GP 1)……………………..3

2.1 Procedure for the diastereomeric enrichment of compound 2 via stirring with silica gel………6

3. Substrate structures……………………………………………………………………………………9

3.1 General procedure for the synthesis of bicyclo[1.1.0]butanes (GP 2)………..………………………10

4. Physical data of the compounds…………………………………………………………………..10

5. X-ray crystal data of 2……………………………………………………………..……………….....25

6. References…………………………………………………………………………………..…...26

7. NMR data……………………………………………………………………………………………28

1. General methods and material

Experimental procedure: All reactions that are air and moisture sensitive were performed in oven-heated glassware under argon atmosphere by using Schlenk-technique.

Solvent and reagents: Anhydrous tetrahydrofuran (THF) was refluxed over elemental Na and freshly distilled from K metal before use. Anhydrous dichloromethane (CH_2Cl_2) was dried over P_4O_{10} and freshly distilled before use. All reagents were purchased from Sigma Aldrich, Acros Organics, ABCR, TCI, Alfa Aesar, BLDPharm and Fluorochem and were used without any further purification. Solvents for column chromatography were purchased in technical grade and purified by distillation prior to use.

TLC: Thin layer chromatography (TLC) was performed on Merck silica gel 60 F-254 plates and visualized by fluorescence quenching under UV light or staining with $KMnO_4$ (1.5 g in 400 mL H_2O, 5 g $NaHCO_3$).

Flash column chromatography (FC): Column chromatography was performed on Merck or VWR silica gel 60 (40-63 μm) using a compress air pressure of 0.3-0.5 bar.

NMR: ^{1}H NMR, ^{13}C NMR and ^{19}F NMR spectra were measured on DPX 300, AV 400 or 500 at 300 K and chemical shift (δ) is expressed in ppm unit. Coupling constants were reported in Hertz (Hz), singlet is defined as s; broad singlet as brs; doublet as d; triplet as t; quartet as q; doublet of doublet as dd; triplet of triplet as tt; multiplet as m.

HRMS (ESI-MS): Spectra were measured on a Thermo Fisher Scientific LTQ XL Orbitrap and Thermo Fisher Scientific Orbitrap Velos Pro spectrometer.

Infrared spectra (IR): Spectra were measured on a Jasco FT/IR-4600 spectrometer and bands are given by wavenumber (cm^{-1}).

Melting points (M.P.): Melting points were measured by Büchi Melting Point *M-560* device and are not corrected.

2. General procedure for 1,3-nitrooxygenation of bicyclo[1.1.0]butanes (GP 1):

In a flame dried Schlenk-tube containing a magnetic stir bar, bicyclo[1.1.0]butane (0.20 mmol, 1.00 equiv.) and TEMPO (0.300 mmol, 1.50 equiv., 46.8 mg), chloroform (2.00 mL) was added in open air. Then ^{t}BuONO (0.400 mmol, 2.00 equiv., 48.0 μL) was added into the reaction mixture and the resulting solution was stirred at 70 °C for 18 h. Then, the solvent was evaporated in rotavapor and directly purified by flash column chromatography (silica gel: Merck silica, column diameter approximately 1.6 cm, column length 15-17 cm, compressed air pressure for column 0.3-0.5 bar, column run time approximately 5-6 hours.) to obtain the desired product.

Note: *The crude reaction mixture should be kept in column chromatography for a minimum of 5 hours. Initially, 300 mL of pentane is used for elution, after which the elution is stopped, allowing the crude mixture to remain in the column for 4 hours. After this, the sample can be eluted over the next hour or longer. The duration for which the crude reaction mixture stays in the column will depend on the polarity of the corresponding compounds.*

We have provided NMR spectral data demonstrating how the diastereomeric ratio (*dr*) was successfully improved from 1.2:1 to >20:1 over time through the column chromatography process (see the ^{1}H NMR spectra below).

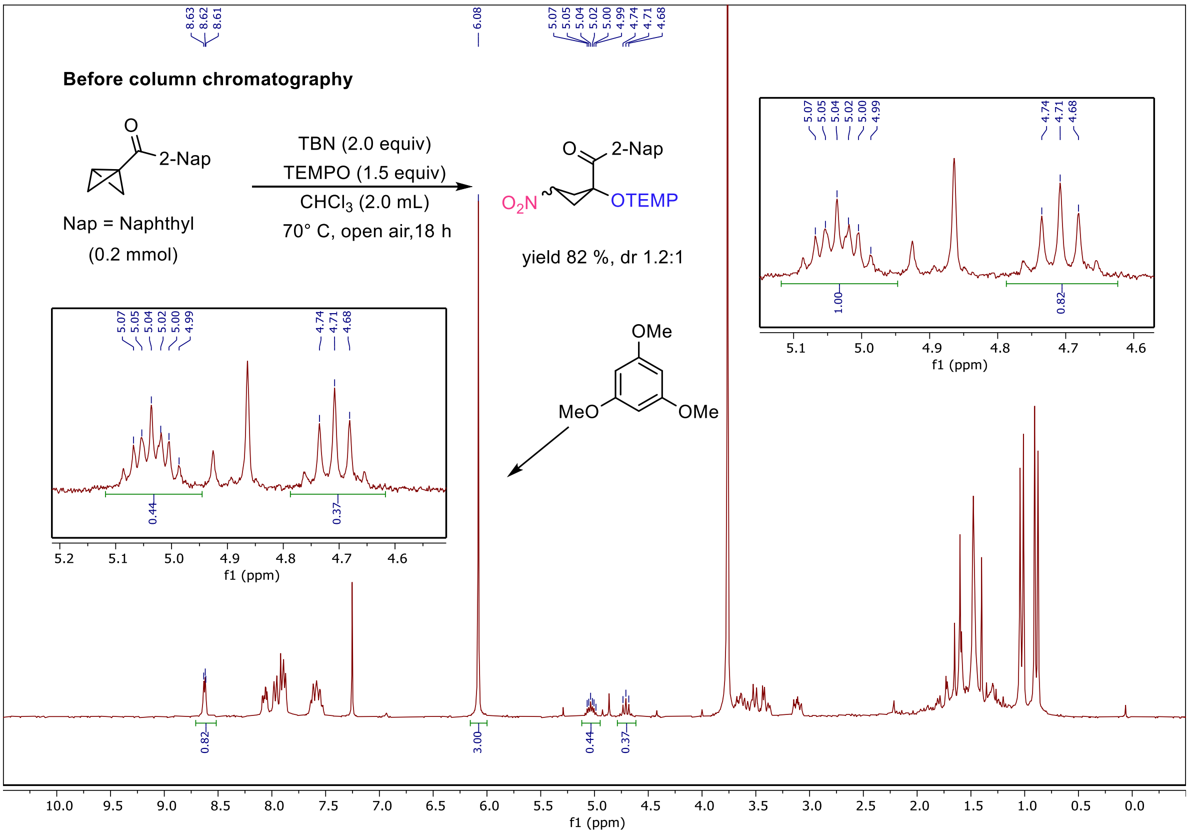

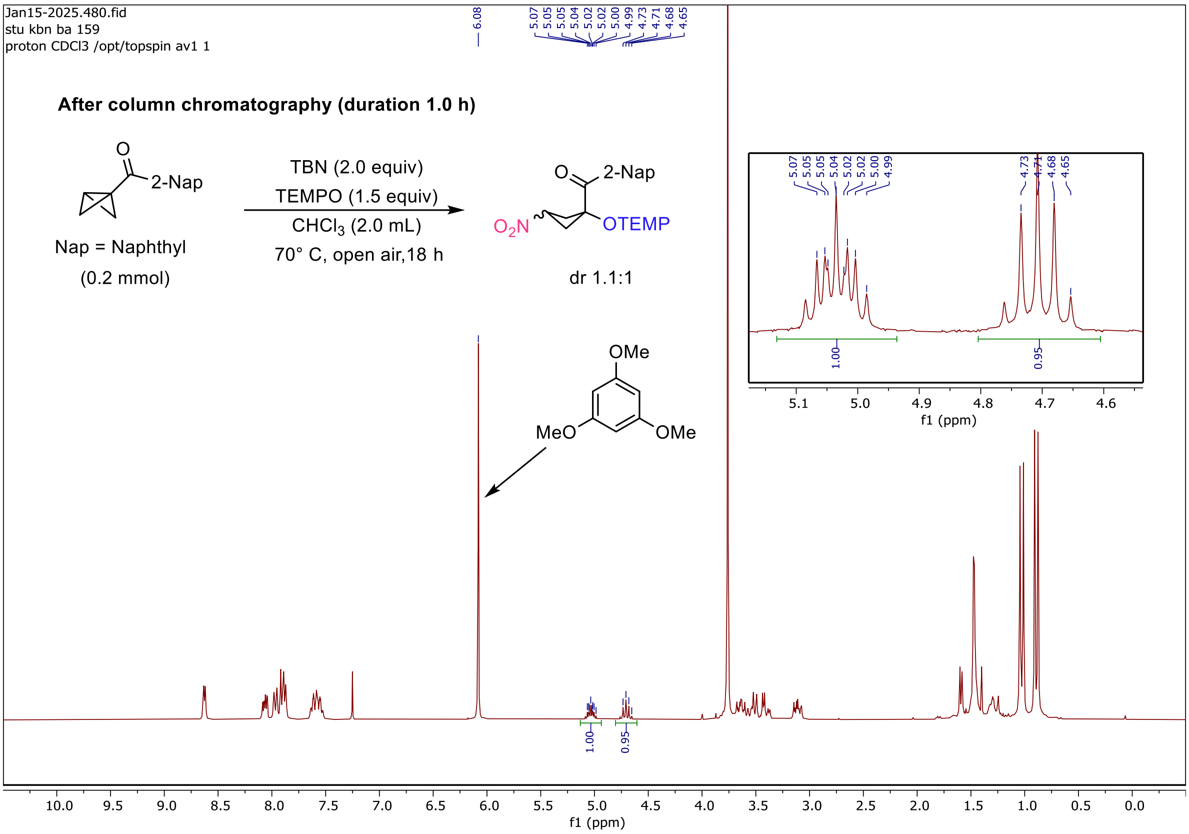
Jan15-2025.480.fid
stu kbn ba 159
proton CDCl3 /opt/topspin av1 1
After column chromatography (duration 1.0 h)
2-Nap
Nap = Naphthyl
(0.2 mmol)
TBN (2.0 equiv)
TEMPO (1.5 equiv)
CHCl3 (2.0 mL)
70° C, open air,18 h
2-Nap
O2N
OTEMP
dr 1.1:1
OMe
MeO
OMe
f1 (ppm)

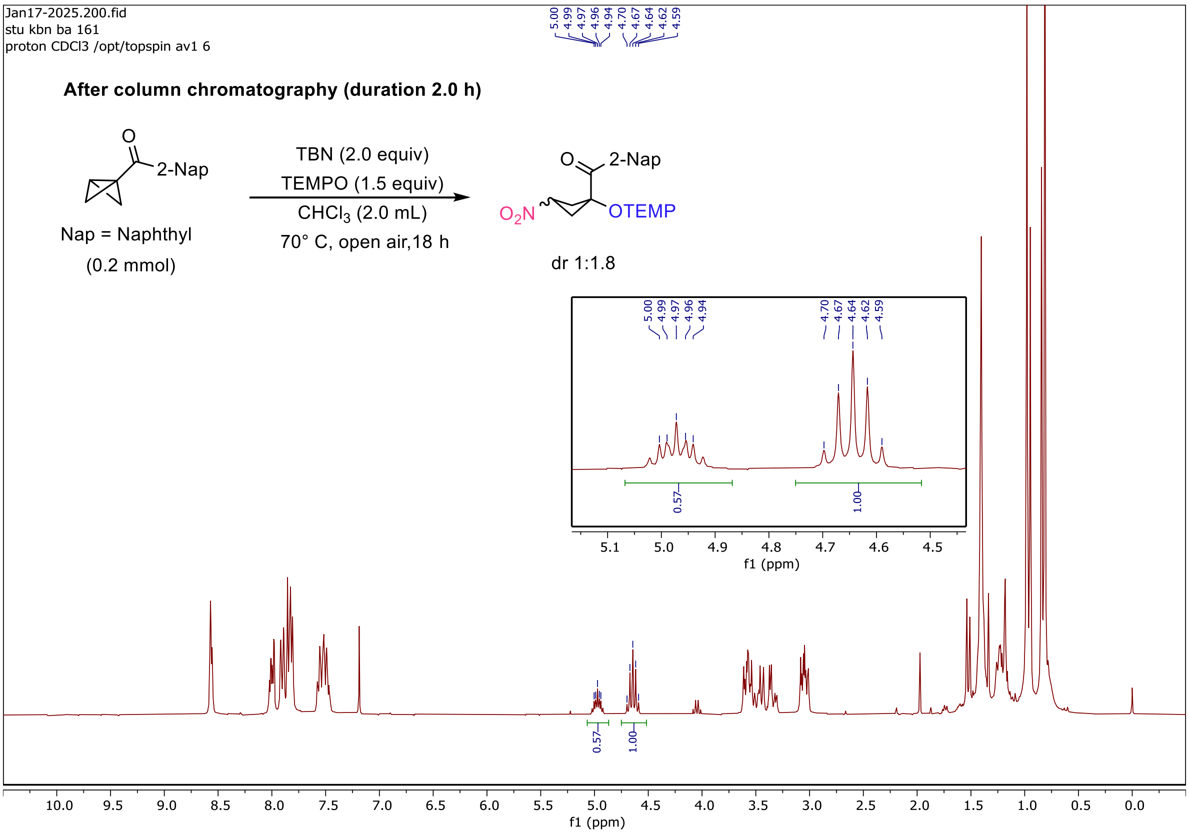
Jan17-2025.200.fid
stu kbn ba 161
proton CDCl3 /opt/topspin av1 6
After column chromatography (duration 2.0 h)
2-Nap
Nap = Naphthyl
(0.2 mmol)
TBN (2.0 equiv)
TEMPO (1.5 equiv)
CHCl3 (2.0 mL)
70° C, open air,18 h
2-Nap
O2N
OTEMP
dr 1:1.8
f1 (ppm)

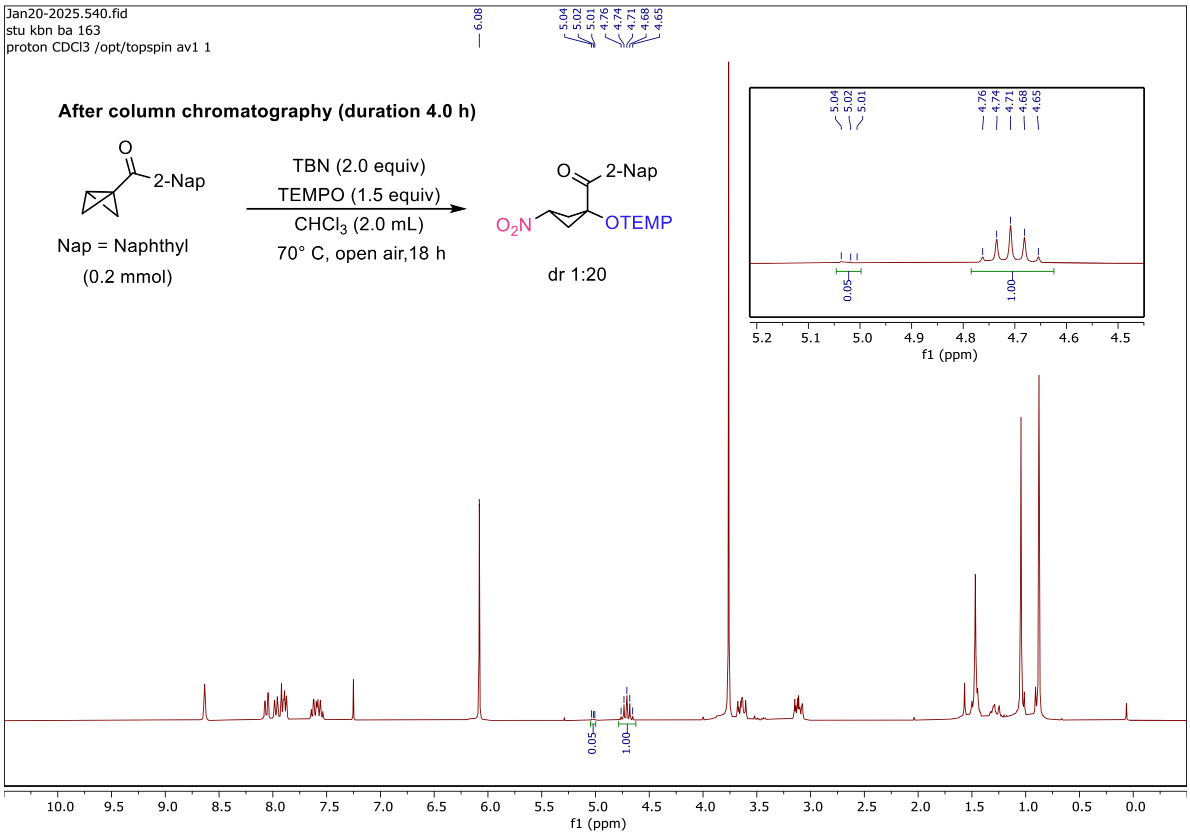
Jan20-2025.540.fid
stu kbn ba 163
proton CDCl3 /opt/topspin av1 1
After column chromatography (duration 4.0 h)
2-Nap
Nap = Naphthyl
(0.2 mmol)
TBN (2.0 equiv)
TEMPO (1.5 equiv)
CHCl3 (2.0 mL)
70° C, open air,18 h
O2N
2-Nap
OTEMP
dr 1:20
6.08
5.04
5.02
5.01
4.76
4.74
4.71
4.68
4.65
0.05
1.00
f1 (ppm)

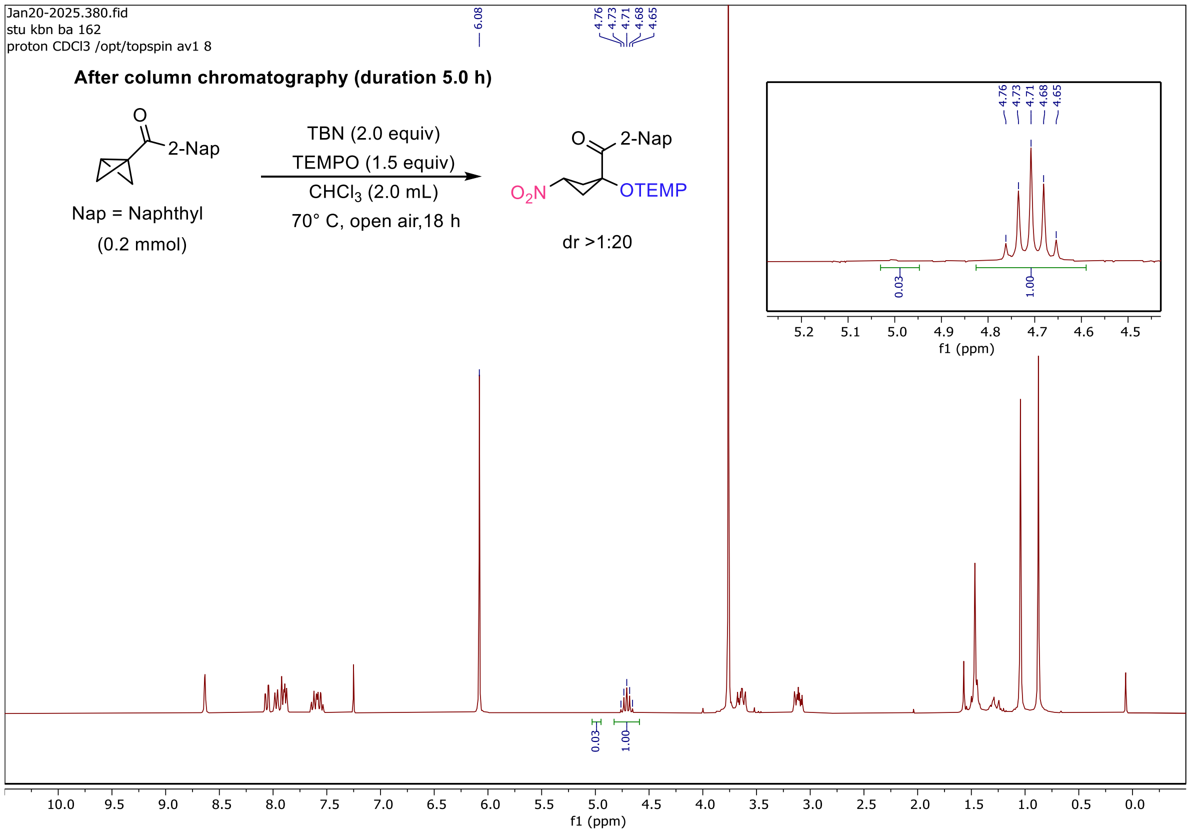
Jan20-2025.380.fid
stu kbn ba 162
proton CDCl3 /opt/topspin av1 8
After column chromatography (duration 5.0 h)
2-Nap
Nap = Naphthyl
(0.2 mmol)
TBN (2.0 equiv)
TEMPO (1.5 equiv)
CHCl3 (2.0 mL)
70° C, open air,18 h
O2N
2-Nap
OTEMP
dr >1:20
6.08
4.76
4.73
4.71
4.68
4.65
0.03
1.00
f1 (ppm)

2.1 Procedure for the diastereomeric enrichment of compound 2 via stirring with silica gel:
In an oven-dried reaction vessel containing a magnetic stir bar, 1,3-nitrooxygenated product **2** (32 mg, *dr* 1:1, 0.078 mmol, 1.0 equiv.) obtained by quick 1 h flash column chromatography, was added. After that, 500 mg Merck silica gel and 5 mL distilled 5% of EtOAc/Pentane were added under open air and the reaction vessel was closed by a septum.
The reaction mixture was stirred overnight at room temperature, then filtered using a sodium sulfate (Na_2SO_4) layer over cotton in a funnel. The silica gel was washed with dichloromethane (DCM, 3 × 5 mL), and the organic solvents were evaporated using a rotary evaporator. This process yielded the 1,3-nitrooxygenated product **2** with an improved diastereomeric ratio (*dr* 6:1) without compromising the overall yield (78%).
To further investigate the effect of prolonged stirring, the same product (*dr* 6:1) was subjected to identical conditions for an additional 24 hours, resulting in a further increase in diastereomeric purity (*dr* 9:1). However, extending the stirring time by an additional 24 hours did not lead to any further enhancement (*dr* remained 9:1).
The results, supported by ^1H NMR spectra (see below), confirm that silica gel plays a crucial role in the diastereomeric enrichment process.

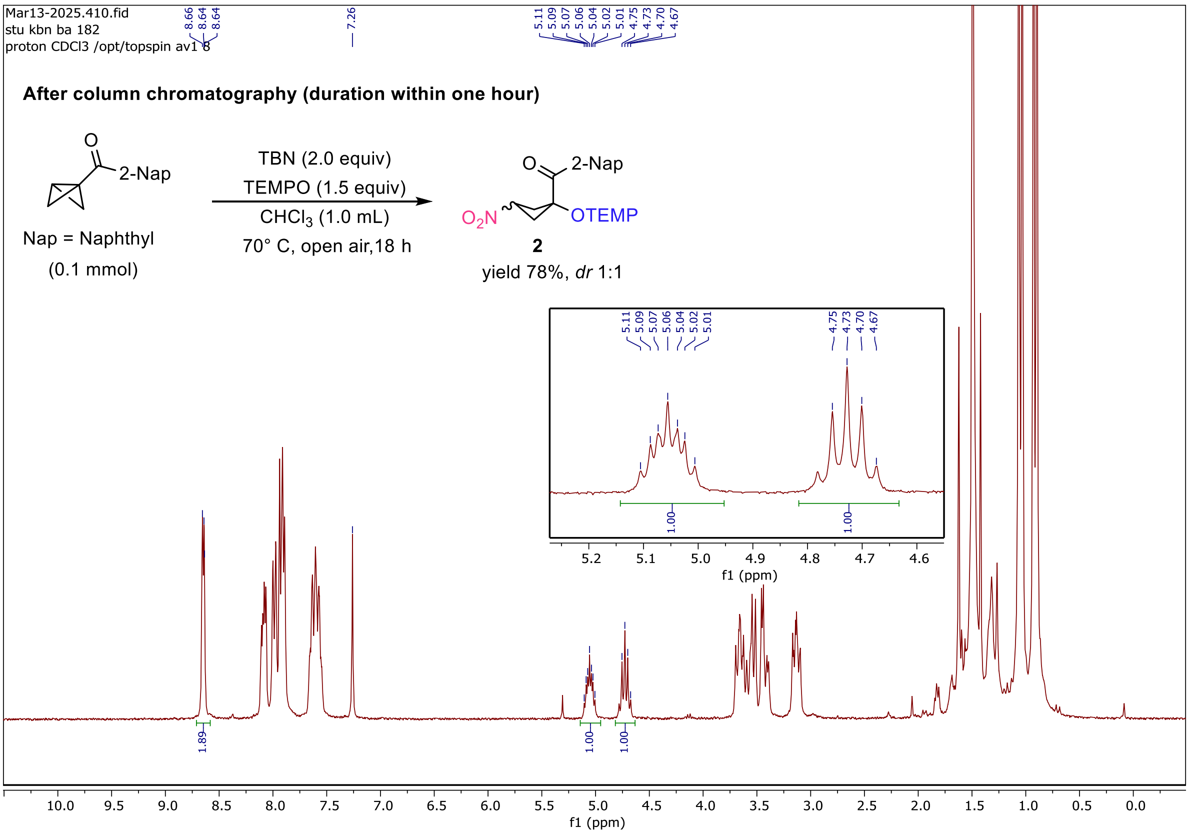

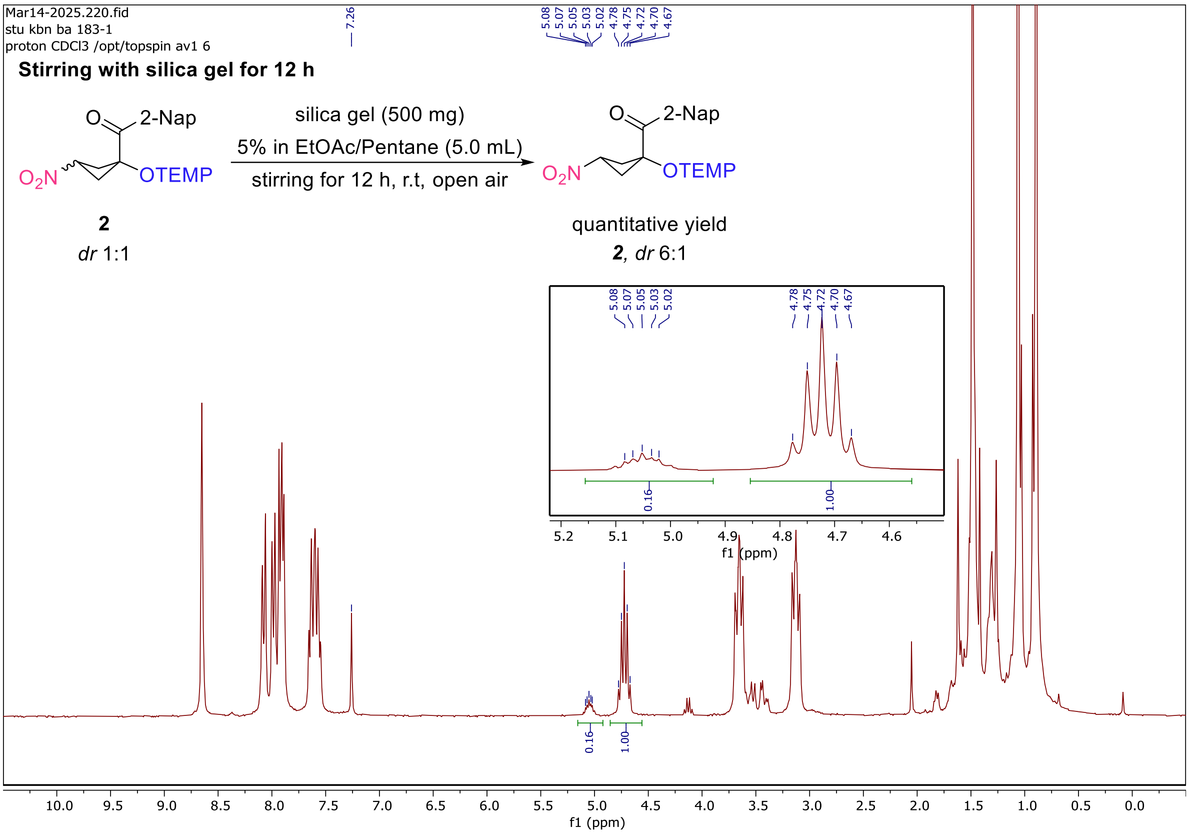
Mar14-2025.220.fid
stu kbn ba 183-1
proton CDCl3 /opt/topspin av1 6
Stirring with silica gel for 12 h
O
2-Nap
O_2N
OTEMP
2
dr 1:1
silica gel (500 mg)
5% in EtOAc/Pentane (5.0 mL)
stirring for 12 h, r.t, open air
O
2-Nap
O_2N
OTEMP
quantitative yield
2, dr 6:1
7.26
5.08 5.07 5.05 5.03 5.02 4.78 4.75 4.72 4.70 4.67
0.16
1.00
f1 (ppm)

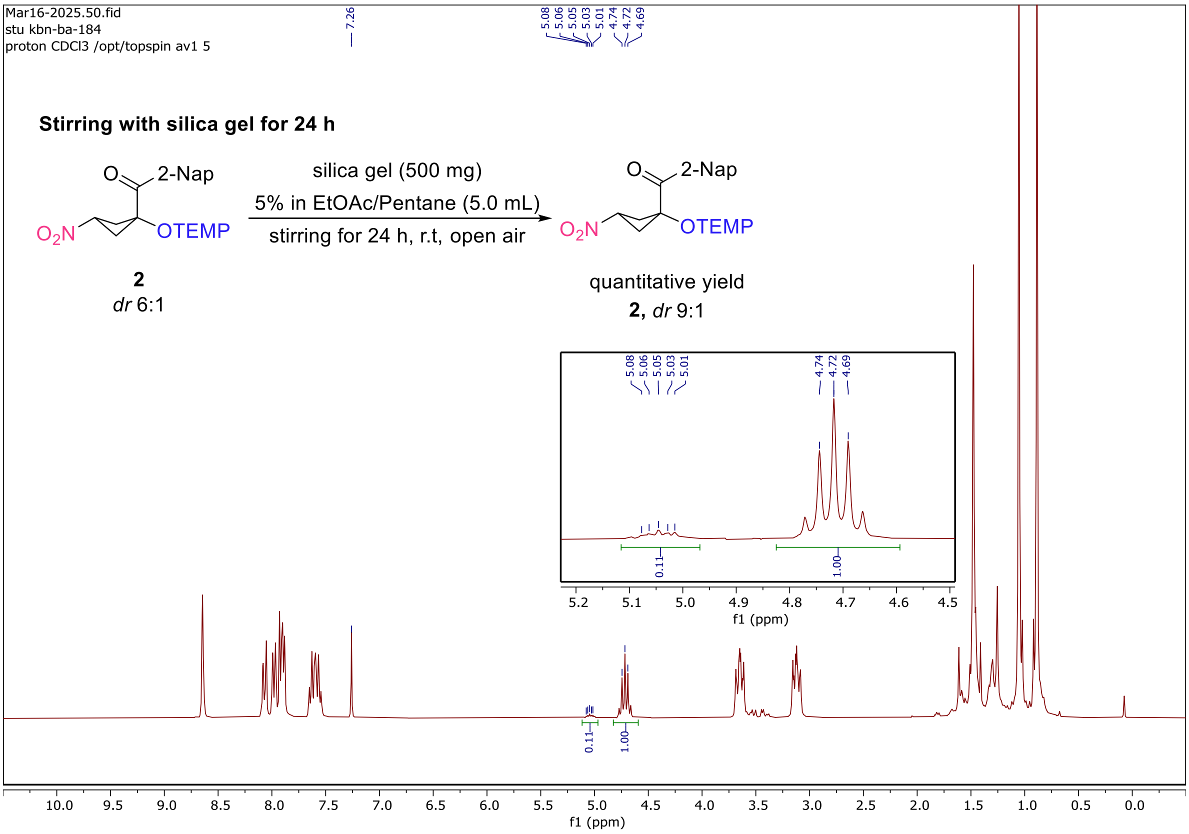
Mar16-2025.50.fid
stu kbn-ba-184
proton CDCl3 /opt/topspin av1 5
Stirring with silica gel for 24 h
O
2-Nap
O_2N
OTEMP
2
dr 6:1
silica gel (500 mg)
5% in EtOAc/Pentane (5.0 mL)
stirring for 24 h, r.t, open air
O
2-Nap
O_2N
OTEMP
quantitative yield
2, dr 9:1
7.26
5.08 5.06 5.05 5.03 5.01 4.74 4.72 4.69
0.11
1.00
f1 (ppm)

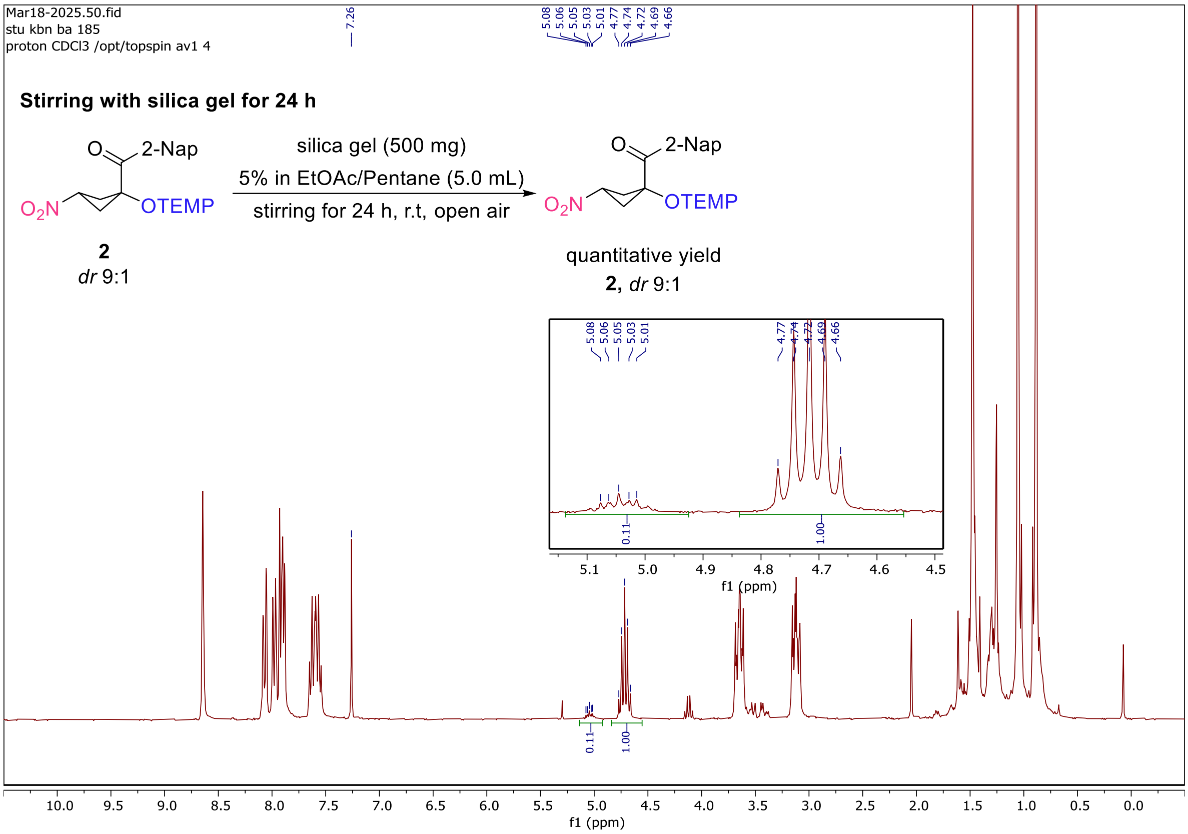
Mar18-2025.50.fid
stu kbn ba 185
proton CDCl3 /opt/topspin av1 4
Stirring with silica gel for 24 h
2-Nap
O_2N
OTEMP
2
dr 9:1
silica gel (500 mg)
5% in EtOAc/Pentane (5.0 mL)
stirring for 24 h, r.t, open air
quantitative yield
2, dr 9:1
f1 (ppm)

3. Substrates structures:

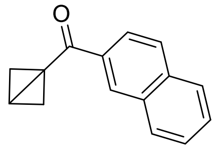

1

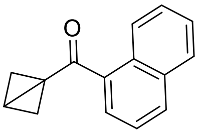

S3

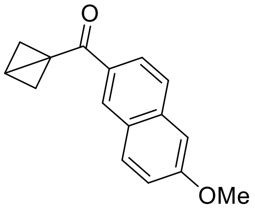

S4

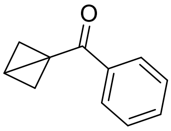

S5

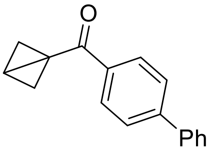

S6

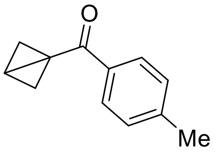

S7

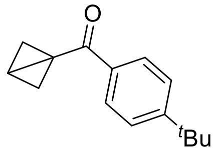

S8

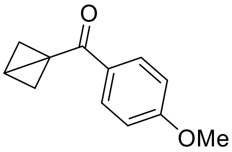

S9

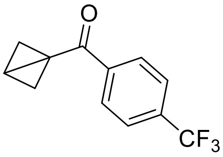

S10

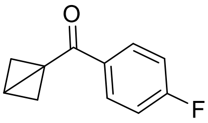

S11

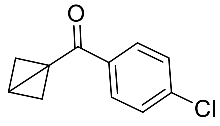

S12

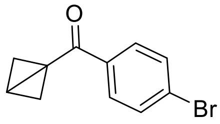

S13

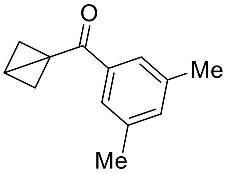

S14

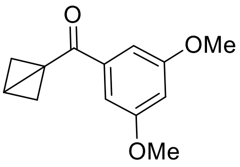

S15

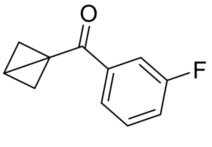

S16

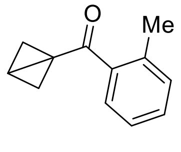

S17

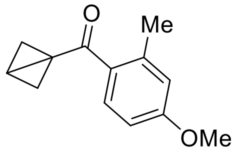

S18

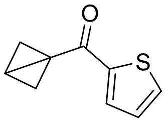

S19

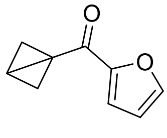

S20

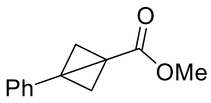

S21

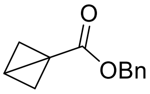

S22

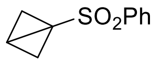

S23

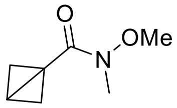

S24

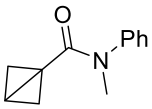

S25

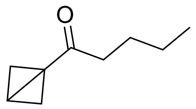

S26

Table S1 Various bicyclo[1.1.0]butanes(BCBs)

Compounds **1**,[1] **S3**,[2] **S4-S7**,[3] **S8**,[4] **S9**,[3] **S10**-S13,[2] **S14**[3], **S15**[2], **S16**,[4] **S17**[2], **S19**,[2] **S20**[2], **S21**,[5] **S22**[6], **S23**[7], and **S24-S26**[3] are all known and were prepared according to the literature known procedure.

3.1 General procedure for the synthesis of bicyclo[1.1.0]butanes (GP 2):

According to the literature known procedures in a flame-dried reaction vessel containing 3-(methoxy(methyl)carbamoyl)cyclobutyl methanesulfonate (712 mg, 3.00 mmol, 1.0 equiv.) in dry THF (20 mL), KO^{t}Bu (freshly made 1M in dry THF, 3.3 mL, 1.1 equiv.) was added in one portion under argon at 0 °C upon vigorous stirring and after that the reaction was continued stirring for 15 minutes. The reaction mixture was quenched with aqueous saturated NH_4Cl (10 mL) at 0 °C. The aqueous layer was then extracted with EtOAc (3x30 mL). The combined organic layers were then washed with brine (50 mL). The organic layer was then dried over $MgSO_4$, filtered and evaporated to afford the N-methoxy-N-methylbicyclo[1.1.0]butane-1-carboxamide, which was used directly without performing any further purification.

In a reaction vessel containing corresponding aryl bromide (3.3 mmol, 1.1 equiv.) in dry THF (15 mL) was cooled to -78 °C and ^{n}BuLi (1.9 mL, 1.6M in hexane) was added and the reaction mixture was stirred under argon for 30 minutes. After that, N-methoxy-N-methylbicyclo[1.1.0]butane-1-carboxamide (dissolved in 3 mL of dry THF) was added into the reaction mixture and stirred for 30 minutes at the same temperature. The reaction mixture was then stirred for 1.5 h at room temperature. Saturated NH_4Cl (10 mL) was added to quench the reaction. The aqueous layer was then extracted with EtOAc (3x30 mL) and combined organic layers were washed with brine (50 mL). The organic layer was then dried over $MgSO_4$, filtered and evaporated and subjected to flash column chromatography to deliver the corresponding aryl keto-bicyclo[1.1.0]butane (BCB).

4. Physical data of the compounds

Naphthalen-2-yl(-3-nitro-1-((2,2,6,6-tetramethylpiperidin-1-yl)oxy)cyclobutyl)methanone (2)

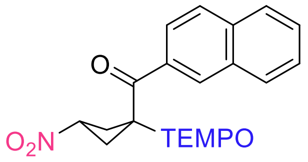

The reaction was performed according to the **GP1**, with bicyclo[1.1.0]butan-1-yl(naphthalen-2-yl)methanone **1** (42 mg, 0.20 mmol, 1 equiv.), *tert*-butyl nitrite (48 μL, 0.40 mmol, 2.0 equiv.) and TEMPO (46.8 mg, 0.300 mmol, 1.50 equiv.) in 2 mL $CHCl_3$ for 18 h. Flash column chromatography (pentane/Et_2O, 100/0 to 98.5/1.5) afforded naphthalen-2-yl(-3-nitro-1-((2,2,6,6-tetramethylpiperidin-1-yl)oxy)cyclobutyl)methanone **2** (61 mg, 74%, *dr* >20:1) as a white solid. **MP**: 117-119 °C.

^{1}H NMR (300 MHz, $CDCl_3$) δ 8.76 – 8.58 (m, 1H), 8.17 – 7.82 (m, 4H), 7.72 – 7.49 (m, 2H), 4.72 (p, *J* = 8.1 Hz, 1H), 3.77 – 3.55 (m, 2H), 3.22 – 2.99 (m, 2H), 1.63 – 1.43 (m, 5H), 1.38 – 1.24 (m, 1H), 1.06 (s, 6H), 0.89 (s, 6H).

^{13}C NMR (76 MHz, $CDCl_3$) δ 199.2, 135.6, 132.3, 130.7, 129.8, 128.7, 128.0, 127.8, 126.7, 125.4, 81.7, 69.6, 59.4, 39.9, 37.0, 33.6, 21.0, 16.9.

HRMS (ESI): $[M+Na]^+$ Calcd for $C_{24}H_{30}N_2O_4Na$ 433.2097; Found: 433.2097.

FTIR (neat): ν(cm^{-1}) 3059, 2973, 2932, 1676, 1626, 1596, 1546, 1466, 1437, 1415, 1363, 1292, 1255, 1233, 1208, 1192, 1178, 1145, 1132, 1119, 1063, 1041, 1020, 971, 934, 908, 865, 819, 807, 792, 772, 761, 731, 649, 624, 564, 506.

Naphthalen-1-yl(-3-nitro-1-((2,2,6,6-tetramethylpiperidin-1-yl)oxy)cyclobutyl)methanone (3)

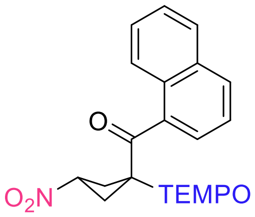

The reaction was performed according to the **GP1**, with bicyclo[1.1.0]butan-1-yl(naphthalen-1-yl)methanone S**3** (42 mg, 0.20 mmol, 1 equiv.), *tert*-butyl nitrite (48 μL, 0.40 mmol, 2.0 equiv.) and TEMPO (46.8 mg, 0.300 mmol, 1.5 equiv.) in 2 mL $CHCl_3$ for 18 h. Flash column chromatography (pentane/Et_2O, 100/0 to 98.5/1.5) afforded naphthalen-1-yl(-3-nitro-1-((2,2,6,6-tetramethylpiperidin-1-yl)oxy)cyclobutyl)methanone **3** (60 mg, 73%, *dr* 20:1) as a white solid. **MP**: 162-164 °C.

^{1}H NMR (300 MHz, $CDCl_3$) δ 8.45 (d, *J* = 8.5 Hz, 1H), 7.99 – 7.83 (m, 2H), 7.82 – 7.73 (m, 1H), 7.57 – 7.31 (m, 3H), 4.80 (p, *J* = 8.1 Hz, 1H), 3.74 – 3.50 (m, 2H), 3.23 – 2.95 (m, 2H), 1.53 – 1.23 (m, 5H), 1.21 – 1.09 (m, 1H), 0.90 (s, 6H), 0.57 (s, 6H).

^{13}C NMR (76 MHz, $CDCl_3$) δ 204.4, 133.8, 132.9, 131.0, 130.8, 129.8, 128.4, 127.7, 126.0, 125.0, 123.4, 81.6, 69.6, 59.2, 39.6, 38.1, 33.1, 20.3, 16.6.

HRMS (ESI): $[M+Na]^+$ Calcd for $C_{24}H_{30}N_2O_4Na$ 433.2097; Found: 433.2096.

FTIR (neat): ν(cm^{-1}) 2973, 2932, 1771, 1733, 1716, 1673, 1654, 1636, 1593, 1543, 1508, 1489, 1457, 1437, 1363, 1285, 1259, 1240, 1179, 1151, 1132, 1114, 1066, 1040, 972, 956, 932, 908, 876, 844, 776, 730, 679, 649, 628, 578, 558, 501.

(6-Methoxynaphthalen-2-yl)(-3-nitro-1-((2,2,6,6-tetramethylpiperidin-1-yl)oxy)cyclobutyl)methanone (4)

The reaction was performed according to the **GP1**, with bicyclo[1.1.0]butan-1-yl(6-methoxynaphthalen-2-yl)methanone **S4** (48 mg, 0.20 mmol, 1 equiv.), *tert*-butyl nitrite (48 μL, 0.40 mmol, 2.0 equiv.) and TEMPO (46.8 mg, 0.300 mmol, 1.50 equiv.) in 2 mL $CHCl_3$ for 18 h. Flash column chromatography (pentane/EtOAc, 100/0 to 98.5/1.5) afforded (6-methoxynaphthalen-2-yl)(-3-nitro-1-((2,2,6,6-tetramethylpiperidin-1-yl)oxy)cyclobutyl)methanone **4** (75.5 mg, 86%, *dr* 12:1) as a white solid. **MP**: 156-158 °C.

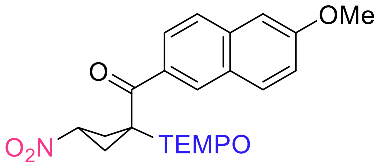

^{1}H NMR (300 MHz, $CDCl_3$, both diastereoisomers) δ 8.46 (s, 1H), 7.94 (dd, *J* = 8.6, 1.7 Hz, 1H), 7.71 (dd, *J* = 23.8, 8.7 Hz, 2H), 7.35 – 6.91 (m, 2H), 4.59 (p, *J* = 8.0 Hz, 1H), 3.82 (s, 3H), 3.64 – 3.39 (m, 2H), 3.25 – 2.84 (m, 2H), 1.47 – 1.28 (m, 5H), 1.23 – 1.12 (m, 1H), 0.94 (s, 6H), 0.79 (s, 6H).

^{13}C NMR (76 MHz, $CDCl_3$, both diastereoisomers) signals corresponding to the two isomers are only partially resolved: δ 198.4, 195.8, 159.5, 159.4, 136.9, 136.8, 131.8, 130.9, 128.1, 127.7, 127.3, 127.2, 126.33, 126.27, 126.0, 125.8, 119.3, 119.2, 105.3, 83.7, 81.2, 72.0, 69.2, 59.0, 55.0, 39.6, 39.5, 36.6, 34.1, 33.2, 32.6, 20.6, 20.4, 16.5.

HRMS (ESI): $[M+Na]^+$ Calcd for $C_{25}H_{32}N_2O_5Na$ 463.2203; Found: 463.2202.

FTIR (neat): ν(cm^{-1}) 2972, 2934, 1672, 1621, 1546, 1503, 1479, 1439, 1415, 1363, 1338, 1291, 1263, 1219, 1197, 1176, 1168, 1138, 1118, 1062, 1029, 972, 956, 904, 875, 856, 841, 816, 791, 764, 728, 649, 621, 570, 522, 505.

(3-Nitro-1-((2,2,6,6-tetramethylpiperidin-1-yl)oxy)cyclobutyl)(phenyl)methanone (5)

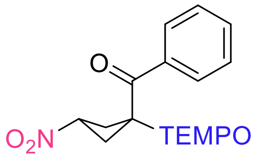

The reaction was performed according to the **GP1**, with bicyclo[1.1.0]butan-1-yl(phenyl)methanone **S5** (32 mg, 0.20 mmol, 1 equiv.), *tert*-butyl nitrite (48 μL, 0.40 mmol, 2.0 equiv.) and TEMPO (46.8 mg, 0.300 mmol, 1.50 equiv.) in 2 mL $CHCl_3$ for 18 h. Flash column chromatography (pentane/Et_2O, 100/0 to 98.5/1.5) afforded (3-nitro-1-((2,2,6,6-tetramethylpiperidin-1-yl)oxy)cyclobutyl)(phenyl)methanone **5** (52.3 mg, 72%, *dr* 13:1) as a white solid. **MP**: 113-115 °C.

^{1}H NMR (300 MHz, $CDCl_3$) δ 8.07 – 7.97 (m, 2H), 7.63 – 7.53 (m, 1H), 7.53 – 7.43 (m, 2H), 4.67 (p, *J* = 8.1 Hz, 1H), 3.65 – 3.49 (m, 2H), 3.15 – 2.95 (m, 2H), 1.56 – 1.41 (m, 5H), 1.35 – 1.23 (m, 1H), 1.00 (s, 6H), 0.88 (s, 6H).

^{13}C NMR (76 MHz, $CDCl_3$) δ 199.5, 133.5, 133.1, 130.0, 128.2, 81.4, 69.5, 59.3, 39.9, 36.9, 33.5, 20.8, 16.9.

HRMS (ESI): $[M+Na]^+$ Calcd for $C_{20}H_{28}N_2O_4Na$ 383.1941; Found: 383.1941.

FTIR (neat): ν(cm^{-1}) 2973, 2932, 1682, 1597, 1581, 1547, 1469, 1448, 1364, 1318, 1289, 1242, 1207, 1179, 1147, 1132, 1115, 1075, 1041, 1016, 1003, 972, 954, 911, 876, 840, 823, 782, 743, 698, 567, 506.

[1,1'-Biphenyl]-4-yl(-3-nitro-1-((2,2,6,6-tetramethylpiperidin-1-yl)oxy)cyclobutyl)methanone (6)

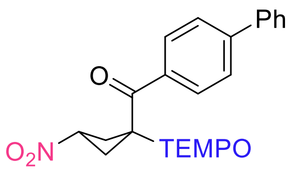

The reaction was performed according to the **GP1**, with [1,1'-biphenyl]-4-yl(bicyclo[1.1.0]butan-1-yl)methanone **S6** (46.8 mg, 0.20 mmol, 1 equiv.), *tert*-butyl nitrite (48 μL, 0.40 mmol, 2.0 equiv.) and TEMPO (46.8 mg, 0.300 mmol, 1.50 equiv.) in 2 mL $CHCl_3$ for 18 h. Flash column chromatography (pentane/Et_2O, 100/0 to 98.5/1.5) afforded [1,1'-biphenyl]-4-yl(-3-nitro-1-((2,2,6,6-tetramethylpiperidin-1-yl)oxy)cyclobutyl)methanone **6** (72.3 mg, 83%, *dr* 8:1) as a white solid. **MP**: 122-124 °C.

^{1}H NMR (300 MHz, $CDCl_3$, both diastereoisomers) δ 8.20 – 8.07 (m, 2H), 7.76 – 7.62 (m, 4H), 7.53 – 7.36 (m, 3H), 4.69 (p, *J* = 8.1 Hz, 1H), 3.72 – 3.52 (m, 2H), 3.23 – 2.94 (m, 2H), 1.57 – 1.43 (m, 5H), 1.37 – 1.26 (m, 1H), 1.05 (s, 6H), 0.94 (s, 6H).

^{13}C NMR (76 MHz, $CDCl_3$, both diastereoisomers) signals corresponding to the two isomers are only partially resolved: δ 198.8, 196.0, 145.6, 145.5, 139.7, 139.6, 132.1, 131.6, 130.7, 130.6, 128.9, 128.8, 128.2, 128.1, 127.1, 126.8, 126.7, 83.9, 81.4, 72.3, 69.5, 65.7, 59.3, 40.0, 39.8, 36.8, 34.30, 33.5, 33.0, 20.9, 20.8, 16.9.

HRMS (ESI): $[M+Na]^+$ Calcd for $C_{26}H_{32}N_2O_4Na$ 459.2254; Found: 459.2255.

FTIR (neat): ν(cm^{-1}) 2973, 2932, 1678, 1603, 1546, 1486, 1469, 1449, 1406, 1363, 1315, 1294, 1242, 1208, 1192, 1179, 1147, 1132, 1115, 1076, 1041, 1007, 972, 953, 908, 876, 854, 832, 793, 774, 766, 746, 730, 696, 669, 649, 620, 568, 550, 505.

(3-Nitro-1-((2,2,6,6-tetramethylpiperidin-1-yl)oxy)cyclobutyl)(p-tolyl)methanone (7)

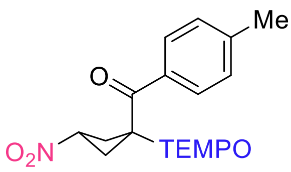

The reaction was performed according to the **GP1**, with bicyclo[1.1.0]butan-1-yl(p-tolyl)methanone **S7** (34.5 mg, 0.200 mmol, 1 equiv.), *tert*-butyl nitrite (48 μL, 0.40 mmol, 2.0 equiv.) and TEMPO (46.8 mg, 0.300 mmol, 1.50 equiv.) in 2 mL $CHCl_3$ for 18 h. Flash column chromatography (pentane/Et_2O, 100/0 to 98.5/1.5) afforded

(3-nitro-1-((2,2,6,6-tetramethylpiperidin-1-yl)oxy)cyclobutyl)(p-tolyl)methanone **7** (66.4 mg, 89%, *dr* >20:1) as a white solid. **MP**: 146-148 °C.
^{1}H NMR (300 MHz, $CDCl_3$) δ 7.85 (d, *J* = 8.3 Hz, 2H), 7.18 (d, *J* = 8.1 Hz, 2H), 4.56 (p, *J* = 8.1 Hz, 1H), 3.56 – 3.36 (m, 2H), 3.09 – 2.83 (m, 2H), 2.33 (s, 3H), 1.49 – 1.31 (m, 5H), 1.28 – 1.17 (m, 1H), 0.92 (s, 6H), 0.81 (s, 6H).
^{13}C NMR (76 MHz, $CDCl_3$) δ 198.9, 144.0, 130.8, 130.2, 129.0, 81.4, 69.6, 59.3, 39.9, 36.9, 33.5, 21.7, 20.9, 16.9.
HRMS (ESI): $[M+Na]^+$ Calcd for $C_{21}H_{30}N_2O_4Na$ 397.2097; Found: 397.2104.
FTIR (neat): ν(cm^{-1}) 2973, 2932, 1678, 1607, 1548, 1468, 1414, 1364, 1315, 1291, 1242, 1206, 1179, 1147, 1132, 1116, 1063, 1040, 1017, 972, 953, 913, 876, 847, 825, 791, 754, 732, 661, 568, 507.

(4-(*tert*-Butyl)phenyl)(-3-nitro-1-((2,2,6,6-tetramethylpiperidin-1-yl)oxy)cyclobutyl)methanone (8)

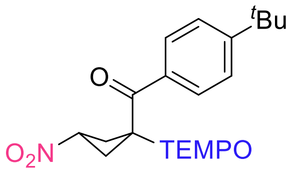

The reaction was performed according to the **GP1**, with bicyclo[1.1.0]butan-1-yl(4-(tert-butyl)phenyl)methanone **S8** (43 mg, 0.20 mmol, 1 equiv.), *tert*-butyl nitrite (48 μL, 0.40 mmol, 2.0 equiv.) and TEMPO (46.8 mg, 0.300 mmol, 1.50 equiv.) in 2 mL $CHCl_3$ for 18 h. Flash column chromatography (pentane/Et_2O, 100/0 to 99/1) afforded (4-(*tert*-butyl)phenyl)(-3-nitro-1-((2,2,6,6-tetramethylpiperidin-1-yl)oxy)cyclobutyl)methanone **8** (62.7 mg, 75%, *dr* 14:1) as a white solid. **MP**: 165-167 °C.
^{1}H NMR (300 MHz, $CDCl_3$, both diastereoisomers) δ 8.04 – 7.92 (m, 2H), 7.53 – 7.42 (m, 2H), 4.63 (p, *J* = 8.1 Hz, 1H), 3.62 – 3.45 (m, 2H), 3.12 – 2.91 (m, 2H), 1.56 – 1.41 (m, 5H), 1.37 – 1.25 (m, 10H), 1.01 (s, 6H), 0.91 (s, 6H).
^{13}C NMR (76 MHz, $CDCl_3$, both diastereoisomers) signals corresponding to the two isomers are only partially resolved: δ 198.7, 195.9, 156.9, 156.7, 130.6, 130.14, 130.06, 125.21, 125.17, 83.9, 81.4, 72.3, 69.5, 59.3, 40.0, 39.9, 36.8, 35.1, 35.0, 34.3, 33.6, 33.0, 31.0, 20.9, 20.8, 16.9.
HRMS (ESI): $[M+Na]^+$ Calcd for $C_{24}H_{36}N_2O_4Na$ 439.2567; Found: 439.2570.
FTIR (neat): ν(cm^{-1}) 2968, 2934, 2871, 1678, 1604, 1548, 1466, 1409, 1364, 1317, 1292, 1242, 1210, 1194, 1180, 1150, 1132, 1109, 1064, 1041, 1018, 973, 954, 909, 876, 852, 824, 792, 768, 731, 709, 649, 571, 546, 506.

(4-Methoxyphenyl)(-3-nitro-1-((2,2,6,6-tetramethylpiperidin-1-yl)oxy)cyclobutyl)methanone (9)

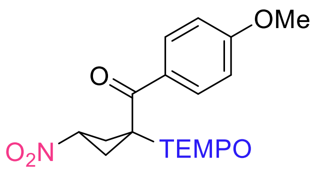

The reaction was performed according to the **GP1**, with bicyclo[1.1.0]butan-1-yl(4-methoxyphenyl)methanone **S9** (37.6 mg, 0.200 mmol, 1 equiv.), *tert*-butyl nitrite (48 μL, 0.40 mmol, 2.0 equiv.) and TEMPO (46.8 mg, 0.300 mmol, 1.5 equiv.) in 2 mL $CHCl_3$ for 18 h. Flash column chromatography (pentane/EtOAc, 100/0 to 98.5/1.5) afforded (4-methoxyphenyl)(-3-nitro-1-((2,2,6,6-tetramethylpiperidin-1-yl)oxy)cyclobutyl)methanone **9** (60.0 mg, 76%, dr 8:1) as a white solid. **MP**: 174-176 °C.
^{1}H NMR (300 MHz, $CDCl_3$, both diastereoisomers) signals corresponding to the two isomers are only partially resolved: δ 8.19 – 7.90 (m, 2H), 7.04 – 6.85 (m, 2H), 4.62 (p, *J* = 8.1 Hz, 1H), 3.86 (s, 3H), 3.59 – 3.41 (m, 2H), 3.14 – 2.91 (m, 2H), 1.58 – 1.39 (m, 5H), 1.35 – 1.24 (m, 1H), 0.99 (s, 6H), 0.90 (s, 6H).
^{13}C NMR (76 MHz, $CDCl_3$, both diastereoisomers) δ 197.5, 195.0, 163.4, 163.3, 132.42, 132.36, 126.0, 113.5, 113.4, 83.7, 81.3, 72.3, 69.5, 59.3, 55.3, 40.0, 39.8, 36.8, 34.2, 33.5, 32.9, 20.9, 20.7, 16.8.

HRMS (ESI): [M+Na]+ Calcd for $C_{21}H_{30}N_2O_5Na$ 413.2046; Found: 413.2047.
FTIR (neat): ν(cm^{-1}) 2975, 2936, 1660, 1601, 1571, 1549, 1510, 1467, 1420, 1365, 1311, 1294, 1260, 1245, 1209, 1173, 1147, 1131, 1116, 1063, 1029, 972, 954, 903, 848, 832, 791, 725, 668, 649, 605, 565, 510.

(3-Nitro-1-((2,2,6,6-tetramethylpiperidin-1-yl)oxy)cyclobutyl)(4-(trifluoromethyl)phenyl)methanone (10)

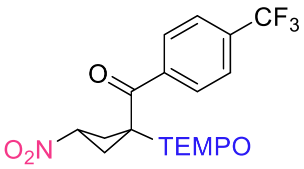

The reaction was performed according to the **GP1**, with bicyclo[1.1.0]butan-1-yl(4-(trifluoromethyl)phenyl)methanone **S10** (45 mg, 0.20 mmol, 1 equiv.), *tert*-butyl nitrite (48 μL, 0.40 mmol, 2.0 equiv.) and TEMPO (46.8 mg, 0.300 mmol, 1.5 equiv.) in 2 mL $CHCl_3$ for 18 h. Flash column chromatography (pentane/Et_2O, 100/0 to 98.5/1.5) afforded (3-nitro-1-((2,2,6,6-tetramethylpiperidin-1-yl)oxy)cyclobutyl)(4-(trifluoromethyl)phenyl)methanone **10** (71.4 mg, 83%, *dr* 10:1) as a colourless oil.
^{1}H NMR (300 MHz, $CDCl_3$, both diastereoisomers) δ 8.13 (d, *J* = 8.6 Hz, 2H), 7.73 (d, *J* = 8.2 Hz, 2H), 4.67 (p, *J* = 8.1 Hz, 1H), 3.68 – 3.51 (m, 2H), 3.15 – 2.93 (m, 2H), 1.52 – 1.40 (m, 5H), 1.36 – 1.25 (m, 1H), 0.99 (s, 6H), 0.86 (s, 6H).
^{13}C NMR (126 MHz, $CDCl_3$, both diastereoisomers) signals corresponding to the two isomers are only partially resolved: 198.40, 195.35, 136.4, 135.8, 134.2 (q, *J* = 32 Hz), 130.3, 130.2, 125.2 (q, *J* = 3 Hz), 123.4 (q, *J* = 273 Hz), 83.8, 81.39, 81.38, 72.0, 69.1, 59.34, 59.32, 56.6, 39.9, 39.8, 36.7, 34.8, 34.2, 33.4, 32.8, 27.3, 20.77, 20.76, 20.64, 20.63, 16.72, 16.70, 16.1.
^{19}F NMR (282 MHz, $CDCl_3$) δ -63.2.
HRMS (ESI): [M+Na]+ Calcd for $C_{21}H_{27}N_2O_4F_3Na$ 451.1815; Found: 451.1816.
FTIR (neat): ν(cm^{-1}) 2975, 2935, 1689, 1549, 1511, 1469, 1411, 1364, 1324, 1290, 1259, 1241, 1208, 1169, 1129, 1114, 1065, 1041, 1017, 973, 954, 911, 876, 856, 831, 792, 780, 767, 733, 696, 650, 595, 563, 506.

(4-Fluorophenyl)(-3-nitro-1-((2,2,6,6-tetramethylpiperidin-1-yl)oxy)cyclobutyl)methanone (11)

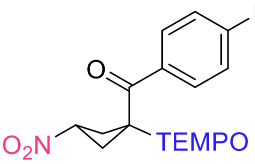

The reaction was performed according to the **GP1**, with bicyclo[1.1.0]butan-1-yl(4-fluorophenyl)methanone **S11** (35 mg, 0.20 mmol, 1 equiv.), *tert*-butyl nitrite (48 μL, 0.40 mmol, 2.0 equiv.) and TEMPO (46.8 mg, 0.300 mmol, 1.5 equiv.) in 2 mL $CHCl_3$ for 18 h. Flash column chromatography (pentane/Et_2O, 100/0 to 98.5/1.5) afforded (4-fluorophenyl)(-3-nitro-1-((2,2,6,6-tetramethylpiperidin-1-yl)oxy)cyclobutyl)methanone **11** (60.5 mg, 80%, *dr* 16:1) as a white solid. **MP**: 97-99 °C.
^{1}H NMR (300 MHz, $CDCl_3$, both diastereoisomers) δ 8.16 – 7.95 (m, 2H), 7.22 – 7.05 (m, 2H), 4.64 (p, *J* = 8.1 Hz, 1H), 3.68 – 3.47 (m, 2H), 3.13 – 2.91 (m, 2H), 1.56 – 1.39 (m, 5H), 1.37 – 1.25 (m, 1H), 0.98 (s, 6H), 0.88 (s, 6H).
^{13}C NMR (76 MHz, $CDCl_3$, both diastereoisomers) signals corresponding to the two isomers are only partially resolved: δ 197.8, 195.0, 165.6 (d, *J* = 255 Hz), 132.9, 132.7 (d, *J* = 9 Hz) 129.8 (d, *J* = 3 Hz), 115.5 (d, *J* = 22 Hz), 83.8, 81.4, 72.2, 69.4, 59.4, 40.0, 39.9, 36.8, 34.3, 33.5, 32.9, 20.9, 20.7, 16.9.
^{19}F NMR (282 MHz, $CDCl_3$) δ -104.3, -104.7.

HRMS (ESI): $[M+Na]^+$ Calcd for $C_{20}H_{27}N_2O_4FNa$ 401.1847; Found: 401.1849.
FTIR (neat): ν(cm^{-1}) 2974, 2934, 1682, 1597, 1548, 1506, 1469, 1410, 1364, 1289, 1237, 1206, 1181, 1157, 1147, 1132, 1114, 1063, 1041, 1014, 972, 953, 911, 876, 851, 839, 791, 761, 733, 695, 661, 568, 531, 504.

(4-Chlorophenyl)(-3-nitro-1-((2,2,6,6-tetramethylpiperidin-1-yl)oxy)cyclobutyl)methanone (12)

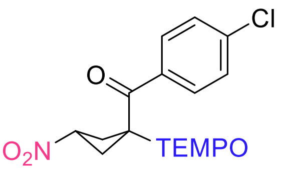

The reaction was performed according to the **GP1**, with bicyclo[1.1.0]butan-1-yl(4-chlorophenyl)methanone **S12** (38.5 mg, 0.20 mmol, 1 equiv.), *tert*-butyl nitrite (48 μL, 0.40 mmol, 2.0 equiv.) and TEMPO (46.8 mg, 0.300 mmol, 1.5 equiv.) in 2 mL $CHCl_3$ for 18 h. Flash column chromatography (pentane/Et_2O, 100/0 to 98.5/1.5) afforded (4-chlorophenyl)(-3-nitro-1-((2,2,6,6-tetramethylpiperidin-1-yl)oxy)cyclobutyl)methanone **12** (74 mg, 94%, dr 8:1) as a white solid. **MP**: 77-79 °C.
^{1}H NMR (300 MHz, $CDCl_3$, both diastereoisomers) δ 8.09 – 7.87 (m, 2H), 7.55 – 7.33 (m, 2H), 4.63 (p, *J* = 8.1 Hz, 1H), 3.71 – 3.41 (m, 2H), 3.15 – 2.90 (m, 2H), 1.58 – 1.39 (m, 5H), 1.37 – 1.25 (m, 1H), 0.97 (s, 6H), 0.87 (s, 6H).
^{13}C NMR (76 MHz, $CDCl_3$, both diastereoisomers) signals corresponding to the two isomers are only partially resolved: δ 198.1, 195.3, 139.6, 139.4, 131.8, 131.5, 131.4, 131.2, 128.62, 128.58, 83.8, 81.3, 72.1, 69.3, 59.3, 39.9, 39.8, 36.7, 34.2, 33.5, 32.9, 20.9, 20.7, 16.8.
HRMS (ESI): $[M+Na]^+$ Calcd for both major isotopes $C_{20}H_{27}N_2O_4ClNa$ 417.1552, 419.1530; Found for both major isotopes: 417.1553, 419.1519.
FTIR (neat): ν(cm^{-1}) 2974, 2933, 1684, 1589, 1548, 1487, 1469, 1400, 1364, 1291, 1242, 1207, 1178, 1148, 1132, 1114, 1091, 1041, 1014, 972, 953, 912, 876, 849, 825, 791, 762, 745, 714, 566, 503.

(4-Bromophenyl)(-3-nitro-1-((2,2,6,6-tetramethylpiperidin-1-yl)oxy)cyclobutyl)methanone (13)

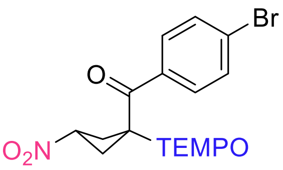

The reaction was performed according to the **GP1**, with bicyclo[1.1.0]butan-1-yl(4-bromophenyl)methanone **S13** (47.5 mg, 0.20 mmol, 1 equiv.), *tert*-butyl nitrite (48 μL, 0.40 mmol, 2.0 equiv.) and TEMPO (46.8 mg, 0.300 mmol, 1.5 equiv.) in 2 mL $CHCl_3$ for 18 h. Flash column chromatography (pentane/Et_2O, 100/0 to 98.5/1.5) afforded (4-bromophenyl)(-3-nitro-1-((2,2,6,6-tetramethylpiperidin-1-yl)oxy)cyclobutyl)methanone **13** (73 mg, 83%, *dr* 13:1) as a white solid. **MP**: 101-103 °C.
^{1}H NMR (300 MHz, $CDCl_3$, both diastereoisomers) δ 8.02 – 7.78 (m, 2H), 7.69 – 7.55 (m, 2H), 4.63 (p, *J* = 8.1 Hz, 1H), 3.65 – 3.47 (m, 2H), 3.10 – 2.88 (m, 2H), 1.57 – 1.36 (m, 5H), 1.36 – 1.21 (m, 1H), 0.97 (s, 6H), 0.87 (s, 6H).
^{13}C NMR (76 MHz, $CDCl_3$, both diastereoisomers) signals corresponding to the two isomers are only partially resolved: δ 198.3, 195.4, 132.2, 131.63, 131.59, 131.5, 128.4, 83.8, 81.4, 72.1, 69.3, 65.7, 59.4, 40.0, 39.8, 36.7, 34.2, 33.5, 32.9, 20.9, 20.8, 16.8, 15.2.
HRMS (ESI): $[M+Na]^+$ Calcd for both major isotopes $C_{20}H_{27}N_2O_4BrNa$ 461.1046, 463.1029; Found: 461.1046, 463.1025.
FTIR (neat): ν(cm^{-1}) 2974, 2933, 1683, 1584, 1547, 1483, 1469, 1397, 1363, 1290, 1241, 1206, 1178, 1146, 1132, 1114, 1070, 1041, 1011, 972, 952, 908, 876, 848, 823, 790, 758, 730, 649, 626, 566, 505.

(3,5-Dimethylphenyl)(-3-nitro-1-((2,2,6,6-tetramethylpiperidin-1-yl)oxy)cyclobutyl)methanone (14)

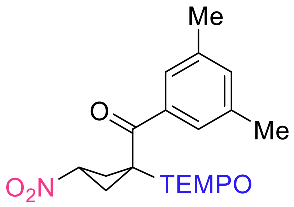

The reaction was performed according to the **GP1**, with bicyclo[1.1.0]butan-1-yl(3,5-dimethylphenyl)methanone **S14** (37.3 mg, 0.200 mmol, 1 equiv.), *tert*-butyl nitrite (48 μL, 0.40 mmol, 2.0 equiv.) and TEMPO (46.8 mg, 0.300 mmol, 1.5 equiv.) in 2 mL $CHCl_3$ for 18 h. Flash column chromatography (pentane/Et_2O, 100/0 to 98.5/1.5) afforded (3,5-dimethylphenyl)(-3-nitro-1-((2,2,6,6-tetramethylpiperidin-1-yl)oxy)cyclobutyl)methanone **14** (72 mg, 92%, *dr* >20:1) as a white solid. **MP**: 134-136 °C.

^{1}H NMR (300 MHz, $CDCl_3$) δ 7.64 (s, 2H), 7.19 (s, 1H), 4.64 (p, *J* = 8.1 Hz, 1H), 3.65 – 3.46 (m, 2H), 3.15 – 2.93 (m, 2H), 2.36 (s, 6H), 1.61 – 1.39 (m, 5H), 1.37 – 1.25 (m, 1H), 1.00 (s, 6H), 0.89 (s, 6H).

^{13}C NMR (76 MHz, $CDCl_3$) δ 199.6, 137.6, 134.8, 133.4, 127.8, 81.4, 69.5, 59.3, 39.8, 36.9, 33.5, 21.2, 20.8, 20.6, 16.9.

HRMS (ESI): $[M+Na]^+$ Calcd for $C_{22}H_{32}N_2O_4Na$ 411.2254; Found: 411.2254.

FTIR (neat): ν(cm^{-1}) 2973, 2932, 1680, 1604, 1549, 1469, 1365, 1314, 1258, 1229, 1197, 1178, 1136, 1114, 1042, 972, 957, 913, 864, 802, 757, 679, 586.

(3,5-Dimethoxyphenyl)(-3-nitro-1-((2,2,6,6-tetramethylpiperidin-1-yl)oxy)cyclobutyl)methanone (15)

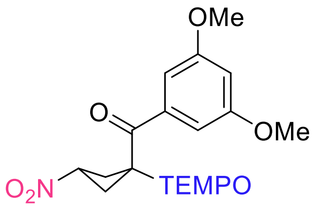

The reaction was performed according to the **GP1**, with bicyclo[1.1.0]butan-1-yl(3,5-dimethoxyphenyl)methanone **S15** (43.6 mg, 0.20 mmol, 1 equiv.), *tert*-butyl nitrite (48 μL, 0.40 mmol, 2.0 equiv.) and TEMPO (46.8 mg, 0.300 mmol, 1.5 equiv.) in 2 mL $CHCl_3$ for 18 h. Flash column chromatography (pentane/EtOAc, 100/0 to 98/2) afforded (3,5-dimethoxyphenyl)(-3-nitro-1-((2,2,6,6-tetramethylpiperidin-1-yl)oxy)cyclobutyl)methanone **15** (73 mg, 87%, *dr* 8:1) as a white solid. **MP**: 106-108 °C.

^{1}H NMR (300 MHz, $CDCl_3$, both diastereoisomers) δ 7.20 (d, *J* = 2.3 Hz, 2H), 6.65 (t, *J* = 2.4 Hz, 1H), 4.62 (p, *J* = 8.1 Hz, 1H), 3.81 (s, 6H), 3.64 – 3.43 (m, 2H), 3.14 – 2.89 (m, 2H), 1.58 – 1.39 (m, 5H), 1.37 – 1.25 (m, 1H), 0.99 (s, 6H), 0.93 (s, 6H).

^{13}C NMR (76 MHz, $CDCl_3$, both diastereoisomers) signals corresponding to the two isomers are only partially resolved: δ 198.4, 195.6, 160.3, 134.8, 134.3, 107.6, 107.5, 105.8, 83.8, 81.4, 72.1, 69.3, 59.3, 55.3, 39.9, 39.8, 36.7, 34.3, 33.4, 32.9, 20.8, 20.7, 16.8.

HRMS (ESI): $[M+Na]^+$ Calcd for $C_{22}H_{32}N_2O_6Na$ 443.2152; Found: 443.2154.

FTIR (neat): ν(cm^{-1}) 2972, 2936, 2841, 1683, 1593, 1550, 1457, 1426, 1363, 1350, 1321, 1306, 1258, 1233, 1205, 1178, 1157, 1133, 1066, 1015, 972, 916, 849, 804, 758.

((3-Fluorophenyl)(-3-nitro-1-((2,2,6,6-tetramethylpiperidin-1-yl)oxy)cyclobutyl)methanone (16)

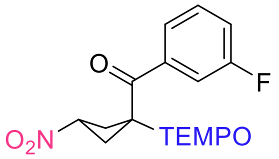

The reaction was performed according to the **GP1**, with bicyclo[1.1.0]butan-1-yl(3-fluorophenyl)methanone **S16** (35.2 mg, 0.200 mmol, 1 equiv.), *tert*-butyl nitrite (48 μL, 0.40 mmol, 2.0 equiv.) and TEMPO (46.8 mg, 0.300 mmol, 1.5 equiv.) in 2 mL $CHCl_3$ for 18 h.

Flash column chromatography (pentane/Et_2O, 100/0 to 98.5/1.5) afforded (3-fluorophenyl)(-3-nitro-1-((2,2,6,6-tetramethylpiperidin-1-yl)oxy)cyclobutyl)methanone **16** (56 mg, 74%, *dr* 9:1) as a white solid. **MP**: 97-99 °C.

^{1}H NMR (300 MHz, $CDCl_3$, both diastereoisomers) δ 7.85 – 7.72 (m, 1H), 7.68 – 7.59 (m, 1H), 7.48 – 7.33 (m, 1H), 7.29 – 7.13 (m, 1H), 4.59 (p, *J* = 8.1 Hz, 1H), 3.62 – 3.41 (m, 2H), 3.06 – 2.87 (m, 2H), 1.49 – 1.33 (m, 5H), 1.32 – 1.19 (m, 1H), 0.93 (s, 6H), 0.82 (s, 6H).

^{13}C NMR (76 MHz, $CDCl_3$, both diastereoisomers) signals corresponding to the two isomers are only partially resolved: δ 198.1 (d, *J* = 2.1 Hz), 195.2, 162.4 (d, *J* = 247.4 Hz),135.5 (d, *J* = 6.5), 129.99, 129.90 (d, *J* = 7.7 Hz), 129.85, 125.90, 125.8 (d, *J* = 3.0 Hz), 120.3, 120.2 (d, *J* = 21.4), 120.0, 116.9, 116.7 (d, J = 22.8 Hz), 116.6, 83.8, 81.4, 72.2, 69.3, 59.4, 40.0, 39.9, 36.8, 34.3, 33.5, 33.4, 33.0, 20.9, 20.8, 16.9.

^{19}F NMR (282 MHz, $CDCl_3$) δ -111.8, -111.8.

HRMS (ESI): $[M+Na]^+$ Calcd for $C_{20}H_{27}N_2O_4FNa$ 401.1847; Found: 401.1848.

FTIR (neat): ν(cm^{-1}) 2974, 2934, 1685, 1586, 1547, 1483, 1470, 1442, 1364, 1294, 1275, 1249, 1224, 1177, 1130, 1063, 1041, 1016, 971, 956, 911, 876, 860, 812, 788, 754, 730, 673, 649, 586, 573, 552, 506.

(3-Nitro-1-((2,2,6,6-tetramethylpiperidin-1-yl)oxy)cyclobutyl)(o-tolyl)methanone (17)

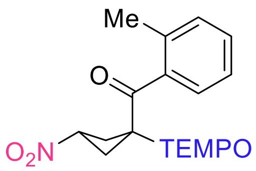

The reaction was performed according to the **GP1**, with bicyclo[1.1.0]butan-1-yl(o-tolyl)methanone **S17** (34.4 mg, 0.20 mmol, 1 equiv.), *tert*-butyl nitrite (48 μL, 0.40 mmol, 2.0 equiv.) and TEMPO (46.8 mg, 0.300 mmol, 1.5 equiv.) in 2 mL $CHCl_3$ for 18 h. Flash column chromatography (pentane/Et_2O, 100/0 to 98.5/1.5) afforded (3-nitro-1-((2,2,6,6-tetramethylpiperidin-1-yl)oxy)cyclobutyl)(o-tolyl)methanone **17** (57 mg, 76%, *dr* 17:1) as a white solid. **MP**: 93-95 °C.

^{1}H NMR (300 MHz, $CDCl_3$, both diastereoisomers) δ 7.67 – 7.54 (m, 1H), 7.36 – 7.26 (m, 1H), 7.24 – 7.12 (m, 2H), 4.73 (p, *J* = 8.1 Hz, 1H), 3.66 – 3.42 (m, 2H), 3.08 – 2.85 (m, 2H), 2.41 (s, 3H), 1.49 – 1.30 (m, 5H), 1.29 – 1.14 (m, 1H), 0.89 (s, 6H), 0.68 (s, 6H).

^{13}C NMR (76 MHz, $CDCl_3$, both diastereoisomers) signals corresponding to the two isomers are only partially resolved: δ 204.5, 200.9, 140.5, 139.4, 134.0, 133.0, 132.2, 132.0, 131.52, 131.49, 130.5, 130.4, 124.64, 124.61, 84.4, 81.6, 72.2, 69.7, 59.3, 40.0, 39.8, 38.0, 35.2, 33.3, 32.6, 21.3, 20.9, 20.5, 20.4, 16.8.

HRMS (ESI): $[M+Na]^+$ Calcd for $C_{21}H_{30}N_2O_4Na$ 397.2097;Found: 397.2099.

FTIR (neat): ν(cm^{-1}) 2972, 2931, 1716, 1683, 1653, 1636, 1601, 1543, 1507, 1489, 1456, 1418, 1363, 1301, 1259, 1241, 1209, 1180, 1150, 1132, 1113, 1083, 1017, 972, 952, 911, 876, 792, 731, 678, 572, 506, 493.

(4-Methoxy-2-methylphenyl)(3-nitro-1-((2,2,6,6-tetramethylpiperidin-1-yl)oxy)cyclobutyl)methanone (18)

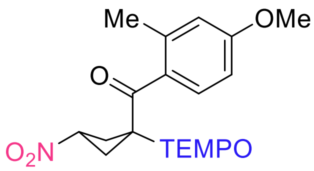

The reaction was performed according to the **GP1**, with bicyclo[1.1.0]butan-1-yl(4-methoxy-2-methylphenyl)methanone **S18** (40.5 mg, 0.20 mmol, 1 equiv.), *tert*-butyl nitrite (48 μL, 0.40 mmol, 2.0 equiv.) and TEMPO (46.8 mg, 0.300 mmol, 1.5 equiv.) in 2 mL $CHCl_3$ for 18 h. Flash column chromatography (pentane/EtOAc, 100/0 to 98.5/1.5) afforded (4-methoxy-2-methylphenyl)(3-nitro-1-((2,2,6,6-

tetramethylpiperidin-1-yl)oxy)cyclobutyl)methanone **18** (64.6 mg, 80%, *dr* 10:1) as a white solid. **MP**: 131-133 °C.

^{1}H NMR (300 MHz, $CDCl_3$, both diastereoisomers) δ 7.79 – 7.65 (m, 1H), 6.81 – 6.68 (m, 2H), 4.72 (p, *J* = 8.1 Hz, 1H), 3.83 (s, 3H), 3.67 – 3.44 (m, 2H), 3.10 – 2.94 (m, 2H), 2.48 (s, 3H), 1.54 – 1.36 (m, 5H), 1.35 – 1.21 (m, 1H), 0.95 (s, 6H), 0.79 (s, 6H).

^{13}C NMR (76 MHz, $CDCl_3$, both diastereoisomers) signals corresponding to the two isomers are only partially resolved: δ 202.0, 198.9, 161.8, 161.7, 144.0, 143.1, 133.3, 133.2, 126.0, 125.2, 117.4, 117.2, 109.93, 109.89, 84.3, 81.5, 72.3, 69.7, 59.2, 55.2, 40.0, 39.8, 37.6, 34.9, 33.2, 32.6, 22.1, 21.8, 20.6, 20.5, 16.8.

HRMS (ESI): $[M+Na]^+$ Calcd for $C_{22}H_{32}N_2O_5Na$ 427.2203; Found: 427.2203.

FTIR (neat): ν(cm^{-1}) 2973, 2933, 1749, 1733, 1716, 1698, 1670, 1635, 1602, 1543, 1507, 1456, 1418, 1363, 1321, 1296, 1239, 1209, 1179, 1111, 1041, 958, 907, 874, 850, 820, 791, 764, 729, 661, 649, 602, 554, 518, 505.

(3-Nitro-1-((2,2,6,6-tetramethylpiperidin-1-yl)oxy)cyclobutyl)(thiophen-2-yl)methanone (19)

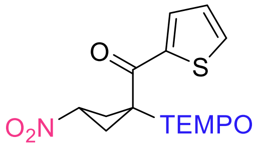

The reaction was performed according to the **GP1**, with bicyclo[1.1.0]butan-1-yl(thiophen-2-yl)methanone **S19** (33 mg, 0.20 mmol, 1 equiv.), *tert*-butyl nitrite (48 μL, 0.40 mmol, 2.0 equiv.) and TEMPO (46.8 mg, 0.300 mmol, 1.5 equiv.) in 2 mL $CHCl_3$ for 18 h. Flash column chromatography (pentane/Et_2O, 100/0 to 98.5/1.5) afforded (3-nitro-1-((2,2,6,6-tetramethylpiperidin-1-yl)oxy)cyclobutyl)(thiophen-2-yl)methanone **19** (58 mg, 79%, *dr* 16:1) as a white solid. **MP**: 89-91 °C.

^{1}H NMR (300 MHz, $CDCl_3$) δ 8.09 – 7.98 (m, 1H), 7.81 – 7.63 (m, 1H), 7.22 – 7.09 (m, 1H), 4.62 (p, *J* = 8.1 Hz, 1H), 3.62 – 3.42 (m, 2H), 3.11 – 2.85 (m, 2H), 1.67 – 1.41 (m, 5H), 1.39 – 1.26 (m, 1H), 1.02 (s, 12H).

^{13}C NMR (76 MHz, $CDCl_3$) δ 191.8, 139.0, 134.8, 134.6, 134.4, 134.3, 128.0, 127.9, 83.7, 81.5, 72.0, 69.4, 59.4, 40.1, 39.9, 36.6, 34.0, 33.5, 32.9, 21.3, 21.0, 16.91, 16.87.

HRMS (ESI): $[M+Na]^+$ Calcd for $C_{18}H_{26}N_2O_4SNa$ 389.1505; Found: 389.1505.

FTIR (neat): ν(cm^{-1}) 2973, 2933, 1656, 1546, 1514, 1469, 1410, 1358, 1296, 1244, 1205, 1180, 1132, 1114, 1080, 1054, 1041, 1017, 972, 956, 909, 876, 857, 812, 790, 724, 648, 620, 561, 522, 505.

(3-Nitro-1-((2,2,6,6-tetramethylpiperidin-1-yl)oxy)cyclobutyl)(furan-2-yl)methanone (20)

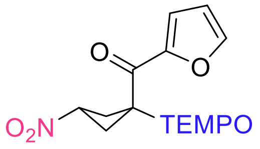

The reaction was performed according to the **GP1**, with bicyclo[1.1.0]butan-1-yl(furan-2-yl)methanone **S20** (29.6 mg, 0.200 mmol, 1 equiv.), *tert*-butyl nitrite (48 μL, 0.40 mmol, 2.0 equiv.) and TEMPO (46.8 mg, 0.300 mmol, 1.5 equiv.) in 2 mL $CHCl_3$ for 18 h. Flash column chromatography (pentane/EtOAc, 100/0 to 98.5/2.5) afforded (3-nitro-1-((2,2,6,6-tetramethylpiperidin-1-yl)oxy)cyclobutyl)(furan-2-yl)methanone **20** (62 mg, 88%, *dr* 11:1) as a white solid. **MP**: 146-148 °C.

^{1}H NMR (300 MHz, $CDCl_3$, both diastereoisomers) δ 7.78 – 7.56 (m, 1H), 7.43 (dd, *J* = 3.6, 0.8 Hz, 1H), 6.69 – 6.43 (m, 1H), 4.59 (p, *J* = 8.1 Hz, 1H), 3.59 – 3.36 (m, 2H), 3.06 – 2.76 (m, 2H), 1.61 – 1.39 (m, 5H), 1.37 – 1.18 (m, 1H), 1.00 (s, 6H), 0.98 (s, 6H).

^{13}C NMR (76 MHz, $CDCl_3$, both diastereoisomers) signals corresponding to the two isomers are only partially resolved: δ 187.3, 185.2, 148.8, 147.5, 147.3, 121.5, 120.7, 112.1, 111.9, 82.9, 80.9, 71.9, 69.2, 59.4, 40.0, 39.9, 36.4, 33.6, 33.5, 32.86, 21.0, 20.7, 16.84, 16.79.
HRMS (ESI): $[M+Na]^+$ Calcd for $C_{18}H_{26}N_2O_5Na$ 373.1733; Found: 373.1736.
FTIR (neat): ν(cm^{-1}) 2976, 2936, 1668, 1548, 1507, 1463, 1391, 1375, 1313, 1254, 1230, 1213, 1133, 1027, 961, 904, 819, 791, 765, 724, 649, 593, 566.

Benzyl-3-nitro-1-((2,2,6,6-tetramethylpiperidin-1-yl)oxy)cyclobutane-1-carboxylate (22)

The reaction was performed according to the **GP1**, with benzyl bicyclo[1.1.0]butane-1-carboxylate **S22** (37.6 mg, 0.200 mmol, 1 equiv.), *tert*-butyl nitrite (48 μL, 0.40 mmol, 2.0 equiv.) and TEMPO (46.8 mg, 0.300 mmol, 1.5 equiv.) in 2 mL $CHCl_3$ for 18 h. Flash column chromatography (pentane/Et_2O, 100/0 to 99/1) afforded benzyl-3-nitro-1-((2,2,6,6-tetramethylpiperidin-1-yl)oxy)cyclobutane-1-carboxylate **22** (68 mg, 87%, *dr* 7:1) as a white solid. **MP**: 91-93 °C.

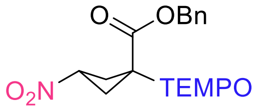

^{1}H NMR (599 MHz, $CDCl_3$, both diastereoisomers) δ 7.42 – 7.31 (m, 5H), 5.23 (s, 1.7H), 5.21 (s, 0.3H), 4.91 – 4.78 (m, 1H), 3.47 – 3.40 (m, 1.7H), 3.33 – 3.26 (m, 0.2H), 3.21 – 3.15 (m, 0.3H), 2.91 – 2.82 (m, 1.8H), 1.61 – 1.52 (m, 1H), 1.50 – 1.38 (m, 4H), 1.35 – 1.27 (m, 1H), 1.11 (s, 6H), 0.96 (s, 5H), 0.94 (s, 1H).
^{13}C NMR (151 MHz, $CDCl_3$, both diastereoisomers) signals corresponding to the two isomers are only partially resolved: δ 173.1, 171.7, 135.1, 135.0, 128.8, 128.7, 128.5, 128.40, 128.35, 79.6, 76.4, 72.0, 69.7, 67.34, 67.26, 59.6, 40.2, 38.2, 36.4, 33.2, 32.7, 20.53, 20.45, 16.9, 16.8.
HRMS (ESI): $[M+Na]^+$ Calcd for $C_{21}H_{30}N_2O_5Na$ 413.2046; Found: 413.2054.
FTIR (neat): ν(cm^{-1}) 2973, 2932, 1725, 1545, 1456, 1363, 1291, 1248, 1195, 1165, 1116, 1082, 1043, 973, 956, 915, 791, 751, 733, 697, 584.

2,2,6,6-tetra Methyl-1-(3-nitro-1-(phenylsulfonyl)cyclobutoxy)piperidine (23)

The reaction was performed according to the **GP1**, with 1-(phenylsulfonyl)bicyclo[1.1.0]butane **S23** (38.8 mg, 0.200 mmol, 1 equiv.), *tert*-butyl nitrite (48 μL, 0.40 mmol, 2.0 equiv.) and TEMPO (46.8 mg, 0.30 mmol, 1.5 equiv.) in 2 mL $CHCl_3$ for 18 h. Flash column chromatography (pentane/EtOAc, 100/0 to 96/4) afforded 2,2,6,6-tetramethyl-1-(3-nitro-1-(phenylsulfonyl)cyclobutoxy)piperidine **23** (55.8 mg, 70%, *dr* >20:1) as a white solid. Decomposed at 160 °C.

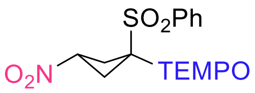

^{1}H NMR (400 MHz, $CDCl_3$) δ 8.03 – 7.95 (m, 2H), 7.73 – 7.66 (m, 1H), 7.63 – 7.55 (m, 2H), 5.08 (p, *J* = 7.8 Hz, 1H), 3.82 – 3.67 (m, 2H), 3.18 – 3.01 (m, 2H), 1.67 – 1.44 (m, 5H), 1.41 – 1.28 (m, 1H), 1.09 (s, 6H), 1.05 (s, 6H).
^{13}C NMR (101 MHz, $CDCl_3$) δ 135.1, 134.3, 130.0, 129.0, 93.8, 69.4, 60.7, 40.5, 37.9, 34.2, 21.5, 16.8.
HRMS (ESI): $[M+Na]^+$ Calcd for $C_{19}H_{28}N_2O_5SNa$ 419.1611; Found: 419.1612.
FTIR (neat): ν(cm^{-1}) 2971, 2939, 1548, 1477, 1447, 1411, 1366, 1308, 1292, 1256, 1227, 1180, 1150, 1132, 1108, 1076, 1045, 977, 893, 869, 787, 760, 735, 717, 688, 646, 567, 552.

N-methoxy-N-methyl-3-nitro-1-((2,2,6,6-tetramethylpiperidin-1-yl)oxy)cyclobutane-1-carboxamide (24)

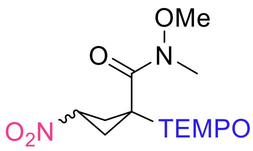

The reaction was performed according to the **GP1**, with *N*-methoxy-*N*-methylbicyclo[1.1.0]butane-1-carboxamide **S24** (28.0 mg, 0.200 mmol, 1.00 equiv.), *tert*-butyl nitrite (48 µL, 0.40 mmol, 2.0 equiv.) and TEMPO (46.8 mg, 0.300 mmol, 1.50 equiv.) in 2.00 mL $CHCl_3$ for 18 h. Flash column chromatography (pentane/EtOAc, 100/0 to 70/30) afforded N-methoxy-N-methyl-3-nitro-1-((2,2,6,6-tetramethylpiperidin-1-yl)oxy)cyclobutane-1-carboxamide **24** (56 mg, 81% yield, *dr* 1:1) as a colourless oil.

^{1}H NMR (300 MHz, $CDCl_3$, both diastereoisomers) δ 4.87 – 4.50 (m, 1H), 3.77-3.15 (m, 9H), 2.99 – 2.92 (m, 1H), 1.53 – 1.43 (m, 5H), 1.33 – 1.29 (m, 1H), 1.09 – 1.00 (m, 12H).

^{13}C NMR (76 MHz, $CDCl_3$ both diastereoisomers) signals corresponding to the two isomers are only partially resolved: δ 172.1, 150.3, 109.8, 80.5, 72.6, 69.4, 61.3, 59.2, 40.3, 40.0, 36.6, 34.5, 33.5, 32.9, 31.7, 29.5, 29.4, 29.1, 22.5, 21.2, 16.9, 16.8, 13.9.

HRMS (ESI): $[M+Na]^+$ Calcd for $C_{16}H_{29}N_3O_5Na$ 366.1995; Found: 366.1999.

N-methyl-3-nitro-N-phenyl-1-((2,2,6,6-tetramethylpiperidin-1-yl)oxy)cyclobutane-1 carboxamide (25)

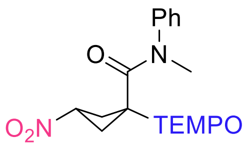

The reaction was performed according to the **GP1**, with *N*-methyl-*N*-phenylbicyclo[1.1.0]butane-1-carboxamide **S25** (562 mg, 3.00 mmol, 1.00 equiv.), *tert*-butyl nitrite (0.71 mL, 6.00 mmol, 2.00 equiv.) and TEMPO (685 mg, 4.50 mmol, 1.50 equiv.) in 30.0 mL $CHCl_3$ for 28 h. Flash column chromatography (5h) (pentane/EtOAc, 100/0 to 95/5) afforded *N*-methyl-3-nitro-*N*-phenyl-1-((2,2,6,6-tetramethylpiperidin-1-yl)oxy)cyclobutane-1 carboxamide **25** (867 mg, 74% yield, *dr* 5:1) as a colorless gel type.

^{1}H NMR (599 MHz, DMSO-d_6, at 90° C, inseparable diastereoisomers) δ 7.43 – 7.40 (m, 2H), 7.31-7.27 (m, 3H), 5.05 – 4.71 (m, 1H), 3.37 – 3.01 (m, 7H), 1.56 – 1.45 (m, 5H), 1.34 – 1.32 (m, 1H), 1.11 – 1.03 (m, 12H).

^{13}C NMR (151 MHz, DMSO-d_6, at 90° C, inseparable diastereoisomers) signals corresponding to the two isomers are only partially resolved: δ 170.8, 144.8, 129.2, 126.9, 72.6, 70.0, 59.6, 40.3, 33.7, 33.3, 21.8, 17.1.

HRMS (ESI): $[M-H]^-$ Calcd for $C_{21}H_{30}N_3O_4$ 388.2230; Found: 388.2240.

1-(3-nitro-1-((2,2,6,6-tetramethylpiperidin-1-yl)oxy)cyclobutyl)pentan-1-one (26)

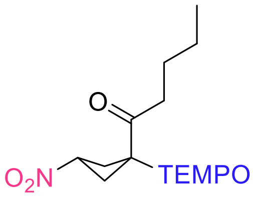

The reaction was performed according to the **GP1**, with 1-(bicyclo[1.1.0]butan-1-yl)pentan-1-one **S26** (138.0 mg, 1.000 mmol, 1.00 equiv.), *tert*-butyl nitrite (0.239 mL, 2.00 mmol, 2.00 equiv.) and TEMPO (228 mg, 1.50 mmol, 1.50 equiv.) in 10.0 mL $CHCl_3$ for 24 h. Flash column chromatography (5h) (pentane/EtOAc, 100/0 to 97/03) afforded 1-(3-nitro-1-((2,2,6,6-tetramethylpiperidin-1-yl)oxy)cyclobutyl)pentan-1-one **26** (184 mg, 57% yield, *dr* 5:1) as a colorless oil.

^{1}H NMR (300 MHz, $CDCl_3$, inseparable diastereoisomers) δ 4.87 – 4.56 (m, 1H), 3.37 – 3.18 (m, 2H), 3.04 – 2.67 (m, 4H), 1.58 – 1.48 (m, 7H), 1.34 – 1.31 (m, 3H), 1.13 – 1.12 (m, 6H), 0.96 – 0. 91 (m, 9H).

^{13}C NMR (76 MHz, $CDCl_3$, inseparable diastereoisomers) signals corresponding to the two isomers are only partially resolved: δ 211.3, 81.6, 69.6, 59.5, 40.2, 37,1 36.8, 33.8, 25.5, 21.0, 17.0, 14.0.
HRMS (ESI): $[M+Na]^+$ Calcd for $C_{18}H_{32}N_2O_4Na$ 363.2265; Found: 363.2249.

4-(3-(2-Naphthoyl)-1-nitro-3-((2,2,6,6-tetramethylpiperidin-1-yl)oxy)cyclobutyl)butan-2-one (27)

A flame dried Schlenk-tube containing **2** (61.5 mg, 0.150 mmol, 1 equiv.) and tetramethylguanidine (9.3 μL, 0.08 mmol, 0.5 equiv.) in 0.9 mL THF was added dropwise into another flame dried Schlenk-tube containing methyl vinyl ketone (15 μL, 0.12 mmol, 1.5 equiv.) at room temperature and the reaction mixture was stirred for 18 h. Water was added into the reaction mixture and the product was extracted with EtOAc. The combined layers were washed with brine, dried over Na_2SO_4, filtrated and evaporated. Flash column chromatography (pentane/EtOAc, 100/0 to 94/6) afforded 4-(3-(2-naphthoyl)-1-nitro-3-((2,2,6,6-tetramethylpiperidin-1-yl)oxy)cyclobutyl)butan-2-one **27** (72 mg, 99%, *dr* 7:1) as a semi-solid.

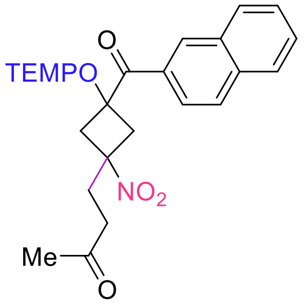

^{1}**H NMR** (300 MHz, $CDCl_3$, both diastereoisomers) δ 8.60 (s, 1H), 8.09 – 8.01 (m, 1H), 7.99 – 7.84 (m, 3H), 7.65 – 7.50 (m, 2H), 3.91 – 3.68 (m, 1.8H), 3.56 – 3.44 (m, 0.3H), 3.37 – 3.26 (m, 0.3H), 3.13 – 2.88 (m, 1.8H), 2.47 – 2.35 (m, 2H), 2.33 – 2.20 (m, 2H), 2.13 (s, 0.4H), 2.10 (s, 2.6H), 1.52 – 1.37 (m, 5H), 1.35 – 1.21 (m, 1H), 1.04 (s, 1H), 0.91 (s, 5H), 0.87 (s, 1H), 0.84 (s, 5H).
^{13}**C NMR** (101 MHz, $CDCl_3$, both diastereoisomers) δ 206.3, 205.8, 198.4, 197.8, 135.6, 135.5, 132.4, 132.33, 132.29, 130.4, 130.3, 129.8, 129.7, 128.7, 128.5, 128.0, 127.9, 127.73, 127.69, 126.7, 126.5, 125.6, 125.5, 83.9, 83.0, 80.9, 80.1, 59.43, 59.36, 40.2, 40.00, 39.95, 39.4, 37.8, 37.5, 33.7, 33.4, 33.0, 31.6, 30.1, 29.9, 20.9, 20.8, 16.8.
HRMS (ESI): $[M+Na]^+$ Calcd for $C_{28}H_{36}N_2O_5Na$ 503.2516; Found: 503.2525.
FTIR (neat): ν(cm^{-1}) 2972, 2932, 1718, 1676, 1626, 1596, 1536, 1466, 1437, 1417, 1375, 1361, 1294, 1271, 1240, 1210, 1166, 1121, 1096, 1061, 1043, 1020, 1003, 973, 910, 866, 821, 798, 774, 763, 730, 649, 565, 541, 506, 475.

3-(3-(2-Naphthoyl)-1-nitro-3-((2,2,6,6-tetramethylpiperidin-1-yl)oxy)cyclobutyl)propanenitrile (28)

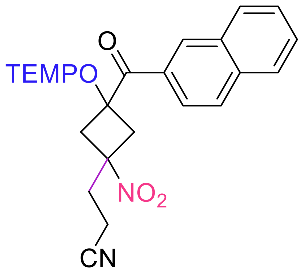

A flame dried Schlenk-tube containing **2** (61.5 mg, 0.150 mmol, 1 equiv.) and tetramethylguanidine (9.3 μL, 0.08 mmol, 0.5 equiv.) in 0.9 mL THF was added dropwise into another flame dried Schlenk-tube containing acrylonitrile (12 μL, 0.12 mmol, 1.5 equiv.) at room temperature and the reaction mixture was stirred for 18 h. Water was added into the reaction mixture and the product was extracted with EtOAc. The combined layers were washed with brine, dried over Na_2SO_4, filtrated and evaporated. Flash column chromatography (pentane/EtOAc, 100/0 to 94/6) afforded 3-(3-(2-naphthoyl)-1-nitro-3-((2,2,6,6-tetramethylpiperidin-1-yl)oxy)cyclobutyl)propanenitrile **28** (53 mg, 76%, *dr* >20:1) as a semi-solid.

^{1}**H NMR** (500 MHz, $CDCl_3$) δ 8.62 (s, 1H), 8.05 (dd, *J* = 8.6, 1.7 Hz, 1H), 7.98 (dd, *J* = 8.2, 1.2 Hz, 1H), 7.94 – 7.88 (m, 2H), 7.66 – 7.61 (m, 1H), 7.60 – 7.55 (m, 1H), 3.91 – 3.79 (m, 2H), 3.13 – 3.04 (m, 2H), 2.46 – 2.38 (m, 2H), 2.36 – 2.26 (m, 2H), 1.52 – 1.41 (m, 5H), 1.35 – 1.27 (m, 1H), 0.92 (s, 6H), 0.86 (s, 6H).
^{13}**C NMR** (126 MHz, $CDCl_3$) δ 199.0, 135.7, 132.5, 132.4, 130.3, 129.8, 128.9, 128.2, 127.8, 126.8, 125.4, 117.6, 80.5, 80.09, 59.5, 40.0, 39.3, 33.0, 32.8, 20.9, 16.9, 12.6.
HRMS (ESI): $[M+Na]^+$ Calcd for $C_{27}H_{33}N_3O_4Na$ 486.2363; Found: 486.2368.
FTIR (neat): ν(cm^{-1}) 2973, 2930, 2871, 1716, 1674, 1626, 1596, 1575, 1540, 1507, 1465, 1417, 1375, 1362, 1299, 1274, 1240, 1213, 1167, 1121, 1062, 1044, 1020, 974, 957, 912, 866, 850, 817, 795, 774, 761, 732, 564, 537, 506.

(3-Allyl-3-nitro-1-((2,2,6,6-tetramethylpiperidin-1-yl)oxy)cyclobutyl)(naphthalen-2-yl)methanone (29a and 29b)

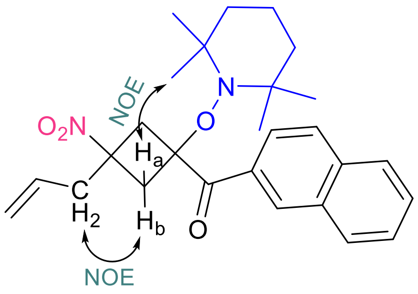

29a

To a flame dried Schlenk-tube containing **2** (82 mg, 0.20 mmol, 1 equiv.) and allylic alcohol (21 μL, 0.30 mmol, 1.5 equiv.) and $Pd(PPh_3)_4$ (23 mg, 0.02 mmol, 0.1 equiv.) and 1.2 mL DMSO was added and then the reaction mixture was bubbled with argon for 6 minutes. After 10 minutes, a CO_2 balloon was installed and another 21 μL allylic alcohol was added and reaction mixture was heated at 80 °C for 18 h. EtOAc was added to the reaction mixture that was then washed with 3x20mL water. Organic layer was dried over $MgSO_4$, filtered and evaporated *in vacuo*. Flash column chromatography (pentane/Et_2O, 100/0 to 98.5/1.5) afforded two separable diastereomeric products **29a** (51 mg, 57%) and **29b** (9.8 mg, 11%) both as colourless oils. The relative configuration was assigned by NOE experiments.

For **29a**

^{1}**H NMR** (300 MHz, $CDCl_3$) δ 8.63 (s, 1H), 8.07 (dd, *J* = 8.7, 1.7 Hz, 1H), 8.02 – 7.86 (m, 3H), 7.75 – 7.45 (m, 2H), 5.84 – 5.36 (m, 1H), 5.28 – 4.97 (m, 2H), 3.95 – 3.61 (m, 2H), 3.19 – 2.92 (m, 2H), 2.75 (d, *J* = 7.1 Hz, 2H), 1.55 – 1.36 (m, 5H), 1.34 – 1.24 (m, 1H), 0.93 (s, 6H), 0.85 (s, 6H).
^{13}**C NMR** (76 MHz, $CDCl_3$) δ 199.0, 135.6, 132.4, 132.3, 130.6, 129.9, 129.8, 128.7, 128.0, 127.7, 126.7, 125.5, 120.3, 81.1, 80.3, 59.4, 42.1, 39.9, 39.2, 33.0, 20.9, 16.9.
HRMS (ESI): $[M+Na]^+$ Calcd for $C_{27}H_{34}N_2O_4Na$ 473.2410; Found: 473.2410.
FTIR (neat): ν(cm^{-1}) 2973, 2933, 1676, 1627, 1597, 1538, 1466, 1437, 1415, 1375, 1362, 1296, 1276, 1260, 1238, 1162, 1131, 1020, 913, 865, 793, 773, 762, 734, 565, 475.

For **29b**

^{1}**H NMR** (300 MHz, $CDCl_3$) δ 8.61 (s, 1H), 8.06 (dd, *J* = 8.6, 1.7 Hz, 1H), 8.00 – 7.83 (m, 3H), 7.67 – 7.50 (m, 2H), 5.73 – 5.55 (m, 1H), 5.21 – 5.05 (m, 2H), 3.58 – 3.46 (m, 2H), 3.39 – 3.25 (m, 2H), 2.90 – 2.79 (m, 2H), 1.64 – 1.41 (m, 5H), 1.37 – 1.27 (m, 1H), 1.04 (s, 6H), 0.88 (s, 6H).
^{13}**C NMR** (76 MHz, $CDCl_3$) δ 197.7, 135.5, 132.4, 132.3, 130.3, 129.8, 129.6, 128.5, 127.9, 127.7, 126.5, 125.7, 120.2, 83.8, 83.1, 59.5, 44.5, 40.1, 39.8, 33.8, 20.9, 16.9.

HRMS (ESI): $[M+Na]^+$ Calcd for $C_{27}H_{34}N_2O_4Na$ 473.2410; Found: 473.2408.
FTIR (neat): ν(cm^{-1}) 2973, 2929, 1680, 1627, 1596, 1542, 1466, 1409, 1375, 1353, 1276, 1179, 1158, 1119, 1010, 989, 973, 916, 864, 790, 763, 748, 628, 563.

(3-((4-(*tert*-Butyl)-3,5-dihydroxyphenyl)(2-hydroxyphenyl)methyl)-3-nitro-1-((2,2,6,6-tetramethylpiperidin-1-yl)oxy)cyclobutyl)(naphthalen-2-yl)methanone (30a and 30b)

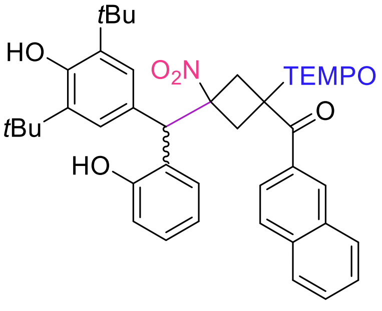

A flame dried Schlenk-tube containing **2** (41 mg, 0.10 mmol, 1 equiv.), 2,6-di-*tert*-butyl-4-(2-hydroxybenzylidene)cyclohexa-2,5-dien-1-one (31 mg, 0.10 mmol, 1 equiv.), Cs_2CO_3 (36 mg, 0.11 mmol, 1.1 equiv.) and $Bi(OTf)_3$ (13 mg, 0.02 mmol, 0.2 equiv.) was added 1.0 mL DCE under argon. The reaction mixture was then stirred at room temperature for 18 h. Solvent was evaporated and flash column chromatography (pentane/EtOAc, 100/0 to 94/6) afforded two separable diastereomeric products **30a** (24.8 mg, 34%) and **30b** (31.9 mg, 44%) both as yellowish oils.

For 30a

^{1}H NMR (300 MHz, $CDCl_3$) δ 8.54 (s, 1H), 7.97 (dd, *J* = 8.7, 1.7 Hz, 1H), 7.89 (d, *J* = 8.0 Hz, 1H), 7.85 – 7.77 (m, 2H), 7.61 – 7.42 (m, 2H), 7.23 – 7.11 (m, 1H), 7.10 – 6.97 (m, 3H), 6.84 – 6.67 (m, 2H), 5.98 (s, 1H), 5.03 (s, 1H), 4.94 (s, 1H), 3.88 – 3.70 (m, 2H), 3.35 – 3.21 (m, 2H), 1.35 – 1.31 (m, 3H), 1.27 (s, 18H), 1.20 – 1.14 (m, 3H), 0.86 (s, 3H), 0.80 (s, 6H), 0.71 (s, 3H).
^{13}C NMR (76 MHz, $CDCl_3$) δ 199.3, 153.9, 153.0, 135.6, 135.4, 132.6, 132.4, 130.5, 130.3, 129.9, 128.7, 128.4, 128.0, 127.7, 126.7, 126.4, 125.6, 125.2, 120.5, 116.6, 84.4, 80.6, 59.5, 49.4, 40.9, 40.0, 34.3, 32.9, 30.2, 21.0, 20.9, 16.9.
HRMS (ESI): $[M+Na]^+$ Calcd for $C_{45}H_{56}N_2O_6Na$ 743.4030; Found: 743.4026.
FTIR (neat): ν(cm^{-1}) 2925, 2855, 1690, 1627, 1597, 1541, 1458, 1377, 1363, 1233, 1155, 1119, 1020, 973, 915, 866, 822, 794, 757, 475.

For 30b

^{1}H NMR (300 MHz, $CDCl_3$) δ 8.64 (s, 1H), 8.09 (dd, *J* = 8.6, 1.7 Hz, 1H), 7.97 (d, *J* = 7.4 Hz, 1H), 7.89 (dd, *J* = 8.5, 3.2 Hz, 2H), 7.69 – 7.46 (m, 2H), 7.20 – 7.12 (m, 3H), 7.09 – 6.98 (m, 1H), 6.80 (t, *J* = 7.5 Hz, 1H), 6.68 (d, *J* = 7.9 Hz, 1H), 5.23 (s, 1H), 5.18 (s, 1H), 4.93 (s, 1H), 3.73 – 3.59 (m, 2H), 3.57 – 3.40 (m, 2H), 1.42 (s, 18H), 1.37 – 1.31 (m, 3H), 1.29 – 1.21 (m, 3H), 0.89 (s, 3H), 0.76 (s, 3H), 0.74 (s, 3H), 0.60 (s, 3H).
^{13}C NMR (76 MHz, $CDCl_3$) δ 195.9, 153.2, 135.8, 135.6, 132.43, 132.38, 130.4, 129.8, 129.6, 128.44, 128.36, 128.3, 127.9, 127.7, 127.2, 126.5, 126.1, 125.9, 120.7, 116.4, 89.1, 84.2, 59.5, 50.7, 39.9, 39.8, 39.7, 34.4, 30.4, 21.1, 20.8, 16.8.
HRMS (ESI): $[M+Na]^+$ Calcd for $C_{45}H_{56}N_2O_6Na$ 743.4030; Found: 743.4023.
FTIR (neat): ν(cm^{-1}) 2926, 2870, 1685, 1626, 1597, 1543, 1457, 1363, 1233, 1151, 1119, 974, 913, 865, 795, 753, 475.

Naphthalen-2-yl(3-nitrocyclobutyl)methanone (31)

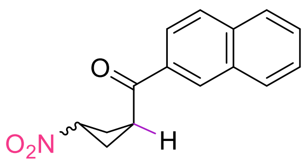

A flame dried Schlenk-tube containing **2** (41 mg, 0.10 mmol, 1 equiv.) and γ-terpinene (48 μL, 0.30 mmol, 3.0 equiv.) was added 5 mL *tert*-butanol under argon. The reaction mixture was then degassed by three freeze-pump-thaw cycles. The reaction mixture then heated at 130 °C for 72 h. Solvent was evaporated and flash column chromatography (pentane/Et_2O, 100/0 to 90/10) afforded naphthalen-2-yl(3-nitrocyclobutyl)methanone **31** (11.6 mg, 44%, *dr* 1.7:1) as a white solid and 20 mg (50%) of starting material could be **2** recovered. **MP**: 89-91 °C.

^{1}H NMR (300 MHz, $CDCl_3$) δ 8.36 (s, 1H), 8.10 – 7.80 (m, 4H), 7.74 – 7.47 (m, 2H), 5.04 (p, *J* = 8.4 Hz, 1H), 4.48 – 4.30 (m, 0.37H), 4.01 – 3.80 (m, 0.63H), 3.29 – 2.80 (m, 4H).

^{13}C NMR (76 MHz, $CDCl_3$) δ 199.0, 197.6, 135.8, 132.4, 132.1, 132.0, 130.2, 130.1, 129.6, 129.5, 128.9, 128.8, 127.84, 127.82, 127.03, 127.01, 123.82, 123.77, 75.7, 72.4, 36.0, 33.4, 31.4, 30.6.

HRMS (ESI): $[M+Na]^+$ Calcd for $C_{15}H_{13}NO_3Na$ 278.0787; Found: 278.0787.

FTIR (neat): ν(cm^{-1}) 3060, 2925, 1671, 1626, 1596, 1540, 1465, 1435, 1361, 1277, 1256, 1219, 1188, 1124, 1095, 1020, 993, 952, 911, 866, 827, 757, 727, 573, 474.

di-*tert*-Butyl 1-(3-(2-naphthoyl)-3-((2,2,6,6-tetramethylpiperidin-1-yl)oxy)cyclobut-1-en-1-yl)hydrazine-1,2-dicarboxylate (32)

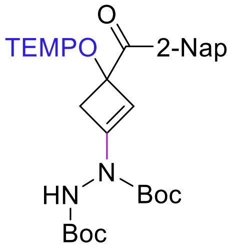

A flame dried Schlenk-tube containing **2** (41 mg, 0.10 mmol, 1 equiv.), di-*tert*-butyl (E)-diazene-1,2-dicarboxylate (37 mg, 0.15 mmol, 1.5 equiv.) and Cs_2CO_3 (35 mg, 0.11 mmol, 1.1 equiv.) was added 1 mL DCE under argon. The reaction mixture was then stirred for 18 h at rt. Solvent was evaporated and flash column chromatography (pentane/EtOAc, 100/0 to 80/20) afforded di-*tert*-butyl 1-(3-(2-naphthoyl)-3-((2,2,6,6-tetramethylpiperidin-1-yl)oxy)cyclobut-1-en-1-yl)hydrazine-1,2-dicarboxylate **32** (29 mg, 48%,) as a white solid. **MP**: 159-161 °C.

^{1}H NMR (300 MHz, $CDCl_3$) δ 8.20 – 8.05 (m, 1H), 7.90 – 7.81 (m, 1H), 7.80 – 7.60 (m, 2H), 7.49 – 7.34 (m, 2H), 6.79 (s, 1H), 6.62 (s, 1H), 5.37 (s, 1H), 4.57 (s, 1H), 1.59 (s, 7H), 1.57 – 1.44 (m, 16H), 1.32 (s, 3H), 1.30 (s, 2H), 1.26 (s, 3H), 0.60 (s, 2H).

^{13}C NMR (76 MHz, $CDCl_3$) δ 204.6, 168.3, 155.3, 151.7, 139.3, 133.3, 132.6, 128.3, 127.9, 127.3, 125.6, 125.4, 123.4, 122.7, 120.8, 85.8, 83.4, 75.5, 68.3, 57.8, 57.1, 45.8, 42.7, 34.5, 33.7, 28.1, 28.0, 25.9, 25.6, 22.3, 17.9, 14.0.

HRMS (ESI): $[M+Na]^+$ Calcd for $C_{34}H_{47}N_3O_6Na$ 616.3357; Found: 616.3348.

FTIR (neat): ν(cm^{-1}) 3191, 2979, 2933, 1740, 1697, 1653, 1635, 1577, 1558, 1541, 1521,1507, 1473, 1457, 1393, 1370, 1291, 1253, 1146,1130, 1113, 1033, 908, 851, 822, 732, 478.

Bicyclo[1.1.0]butan-1-yl(4-methoxy-2-methylphenyl)methanone (S18)

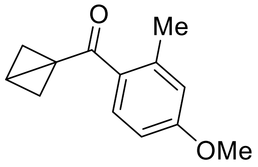

Applying the general procedure 2 (**GP 2**), using 3-(methoxy(methyl)carbamoyl)cyclobutyl methanesulfonate (712 mg, 3.00 mmol, 1.0 equiv.) and 1-bromo-4-methoxy-2-methylbenzene (663 mg, 3.30 mmol, 1.1 equiv.). Flash column chromatography (pentane/EtOAc, 100/0 to 96.5/3.5) afforded bicyclo[1.1.0]butan-1-yl(4-methoxy-2-methylphenyl)methanone **S18** (327 mg, 49%) as a colourless oil.

^{1}**H NMR** (400 MHz, $CDCl_3$) δ 7.56 – 7.49 (m, 1H), 6.76 – 6.67 (m, 2H), 3.81 (s, 3H), 2.46 – 2.42 (m, 2H), 2.41 (s, 3H), 2.15 (p, *J* = 3.3 Hz, 1H), 1.45 – 1.33 (m, 2H).
^{13}**C NMR** (101 MHz, $CDCl_3$) δ 201.7, 161.0, 139.3, 131.3, 130.6, 116.4, 110.1, 55.2, 37.5, 21.0, 20.6, 19.1. **HRMS** (ESI): $[M+Na]^+$ Calcd for $C_{13}H_{14}O_2N$ 225.0886; Found: 225.0883.

5. X-ray crystal data of 2

X-Ray diffraction: Data sets for compound **2** were collected with a Bruker D8 Venture Photon III Diffractometer. Programs used: data collection: *APEX4* Version 2021.4-0 [8] (Bruker AXS Inc., **2021**); cell refinement: *SAINT* Version 8.40B (Bruker AXS Inc., **2021**); data reduction: *SAINT* Version 8.40B (Bruker AXS Inc., **2021**); absorption correction, *SADABS* Version 2016/2 (Bruker AXS Inc., **2021**); structure solution *SHELXT*-Version 2018-3 [9] (Sheldrick, G. M. *Acta Cryst.*, **2015**, *A71*, 3-8); structure refinement *SHELXL*- Version 2018-3 [10] (Sheldrick, G. M. *Acta Cryst.*, **2015**, *C71* (1), 3-8) and graphics, *XP* [11] (Version 5.1, Bruker AXS Inc., Madison, Wisconsin, USA, **1998**). *R*-values are given for observed reflections, and wR^2 values are given for all reflections.
Exceptions and special features: For compound **2** the NO_2 group was found disordered over two positions in the asymmetric unit. Several restraints (SADI, SAME, ISOR and SIMU) were used in order to improve refinement stability.

X-ray crystal structure analysis of 2: A colorless, prism-like specimen of $C_{24}H_{30}N_2O_4$, approximate dimensions 0.063 mm x 0.156 mm x 0.173 mm, was used for the X-ray crystallographic analysis. The X-ray intensity data were measured on a single crystal diffractometer Bruker D8 Venture Photon III system equipped with a micro focus tube Cu ImS (CuKα, λ = 1.54178 Å) and a MX mirror monochromator. A total of 748 frames were collected. The total exposure time was 5.97 hours. The frames were integrated with the Bruker SAINT software package using a wide-frame algorithm. The integration of the data using an orthorhombic unit cell yielded a total of 22885 reflections to a maximum θ angle of 66.65° (0.84 Å resolution), of which 3797 were independent (average redundancy 6.027, completeness = 98.5%, R_{int} = 4.39%, R_{sig} = 2.74%) and 3598 (94.76%) were greater than $2\sigma(F^2)$. The final cell constants of a = 8.4366(2) Å, b = 11.2441(2) Å, c = 22.9481(5) Å, volume = 2176.90(8) $Å^3$, are based upon the refinement of the XYZ-centroids of 9889 reflections above 20 σ(I) with 7.705° < 2θ < 133.1°. Data were corrected for absorption effects using the multi-scan method (SADABS). The ratio of minimum to maximum apparent transmission was 0.881. The calculated minimum and maximum transmission coefficients (based on crystal size) are 0.8900 and 0.9580. The structure was solved and refined using the Bruker SHELXTL Software Package, using the space group $P2_12_12_1$, with Z = 4 for the formula unit, $C_{24}H_{30}N_2O_4$. The final anisotropic full-matrix least-squares refinement on F^2 with 297 variables converged at R1 = 2.91%, for the observed data and wR2 = 6.99% for all data. The goodness-of-fit was 1.053. The largest peak in the final difference electron density synthesis was 0.158 $e^-/Å^3$ and the largest hole was -0.139 $e^-/Å^3$ with an RMS deviation of 0.029 $e^-/Å^3$. On the basis of the final model, the calculated density was 1.253 g/cm^3 and F(000), 880 e^-. Flack parameter was refined to 0.01(8). CCDC Nr.: 2411255.

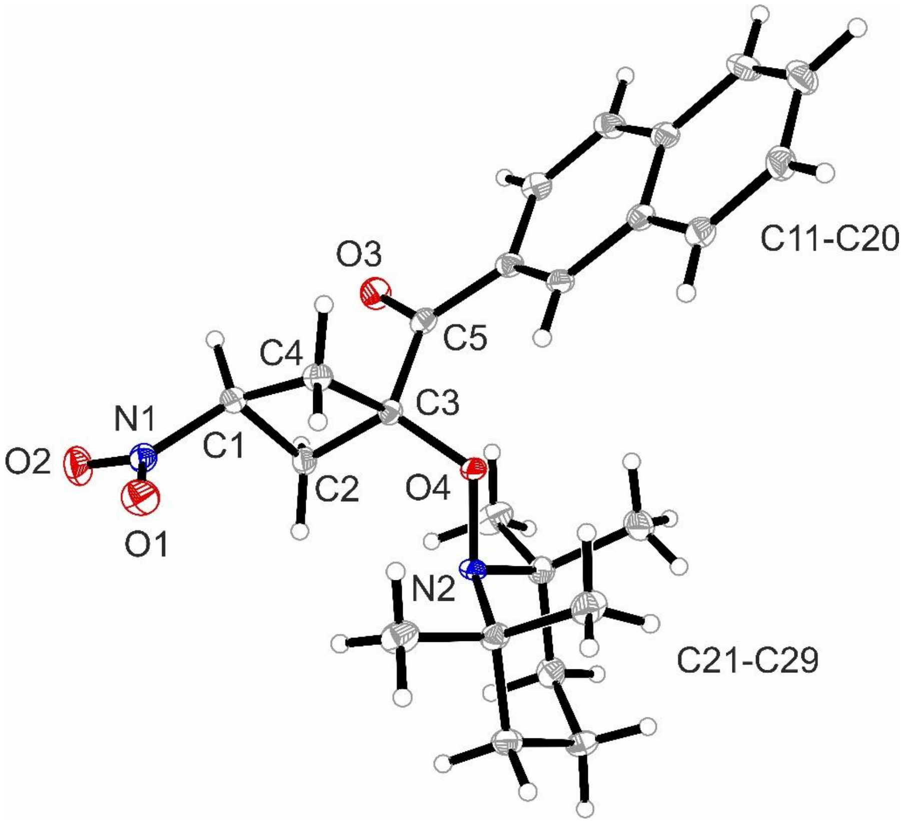

Figure S1: Crystal structure of compound **2**.
Thermal ellipsoids are shown at 50% probability.

6. References

1. R. Guo, Y.-C. Chang, L. Herter, C. Salome, S. E. Braley, T. C. Fessard and M. K. Brown, *J. Am. Chem. Soc.*, 2022, **144**, 7988–7994.

2. L. Yang, H. Wang, M. Lang, J. Wang and S. Peng, *Org. Lett.*, 2024, **26**, 4104–4110.

3. S. Dutta, C. G. Daniliuc, C. Mück-Lichtenfeld and A. Studer, *J. Am. Chem. Soc.*, 2024, **146**, 27204–27212.

4. D. Sarkar, S. Deswal, R. Chandra Das and A. T. Biju, *Chem. Sci.*, 2024, **15**, 16243–16249.

5. R. M. Bychek, V. Hutskalova, Y. P. Bas, O. A. Zaporozhets, S. Zozulya, V. V. Levterov and P. K. Mykhailiuk, *J. Org. Chem.*, 2019, **84**, 15106–15117.

6. B. D. Schwartz, A. P. Smyth, P. E. Nashar, M. G. Gardiner and L. R. Malins, *Org. Lett.*, 2022, **24**, 1268–1273.

7. X. Wu, W. Hao, K.-Y. Ye, B. Jiang, G. Pombar, Z. Song and S. Lin, *J. Am. Chem. Soc.*, 2018, **140**, 14836–14843.

8. Bruker AXS (2021) APEX4 Version 2021.4-0, SAINT Version 8.40B and SADABS Bruker AXS area detector scaling and absorption correction Version 2016/2, Bruker AXS Inc., Madison, Wisconsin, USA.

9. Sheldrick, G. M., SHELXT – Integrated space-group and crystal-structure determination, *Acta Cryst*., 2015, A71, 3-8.

10. Sheldrick, G.M., Crystal structure refinement with SHELXL, *Acta Cryst.,* 2015, C71 (1), 3-8.

11. Bruker AXS (1998) XP – Interactive molecular graphics, Version 5.1, Bruker AXS Inc., Madison, Wisconsin, USA.

7. NMR Spectra

^{1}H NMR of 2 ($CDCl_3$, 300 MHz)

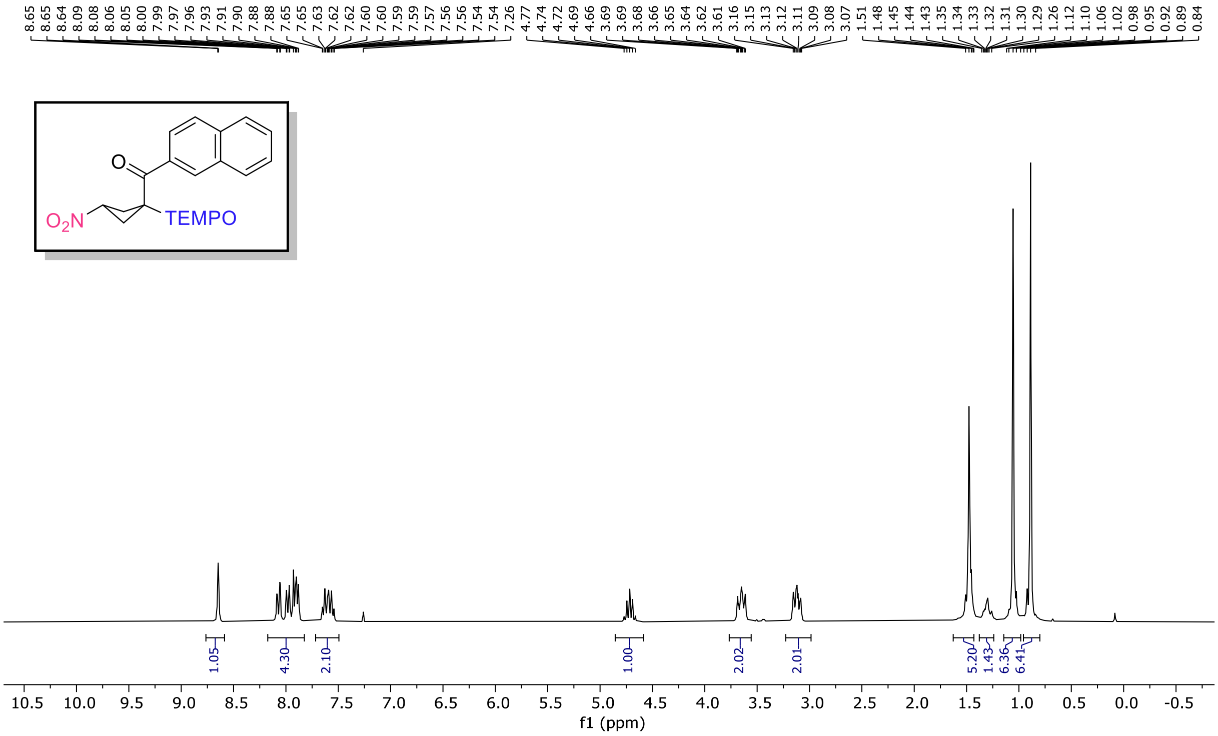

^{13}C NMR of 2 ($CDCl_3$, 76 MHz)

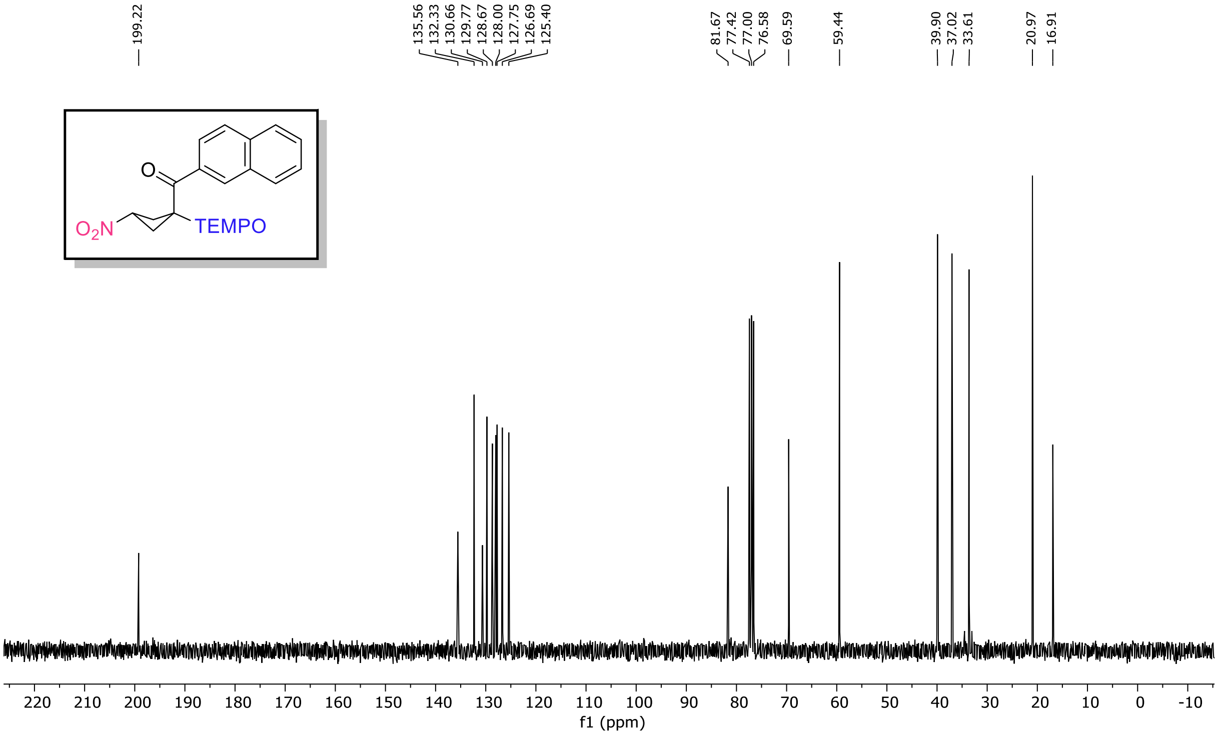

^{1}H NMR of 3 (CDCl$_3$, 300 MHz)

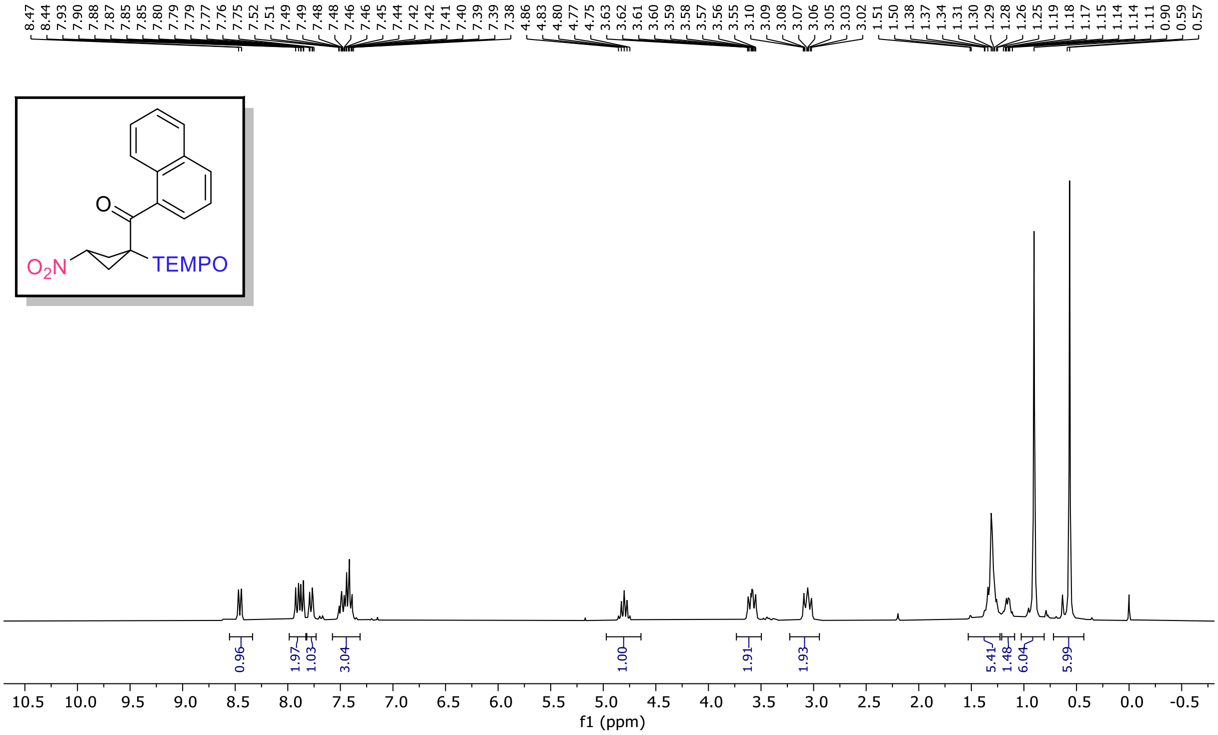

^{13}C NMR of 3 (CDCl$_3$, 76 MHz)

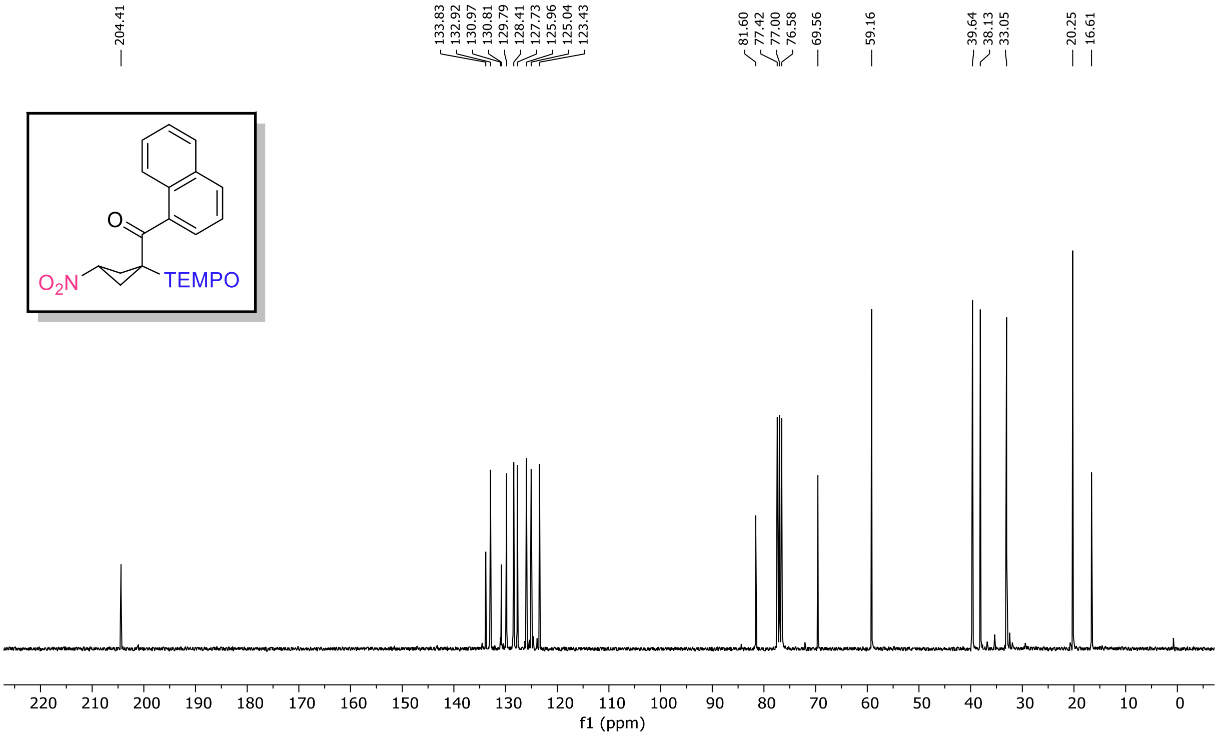

^{1}H NMR of 4 (CDCl$_3$, 300 MHz)

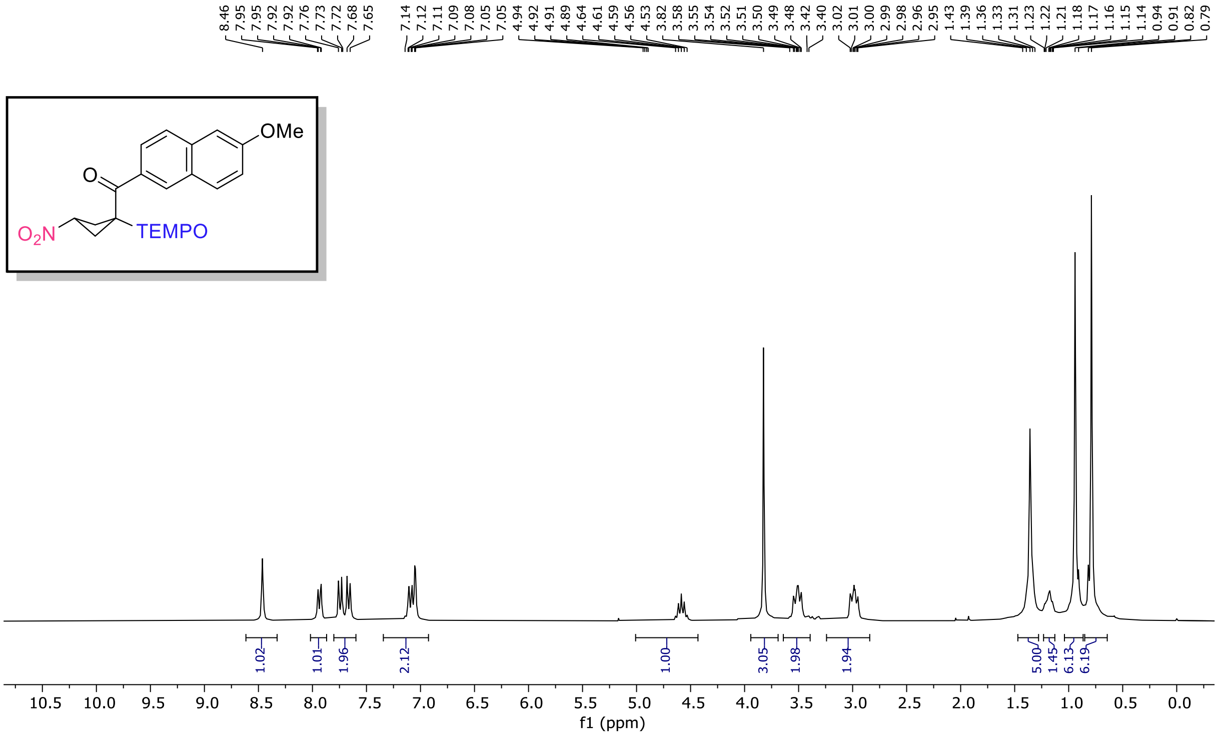

^{13}C NMR of 4 (CDCl$_3$, 76 MHz)

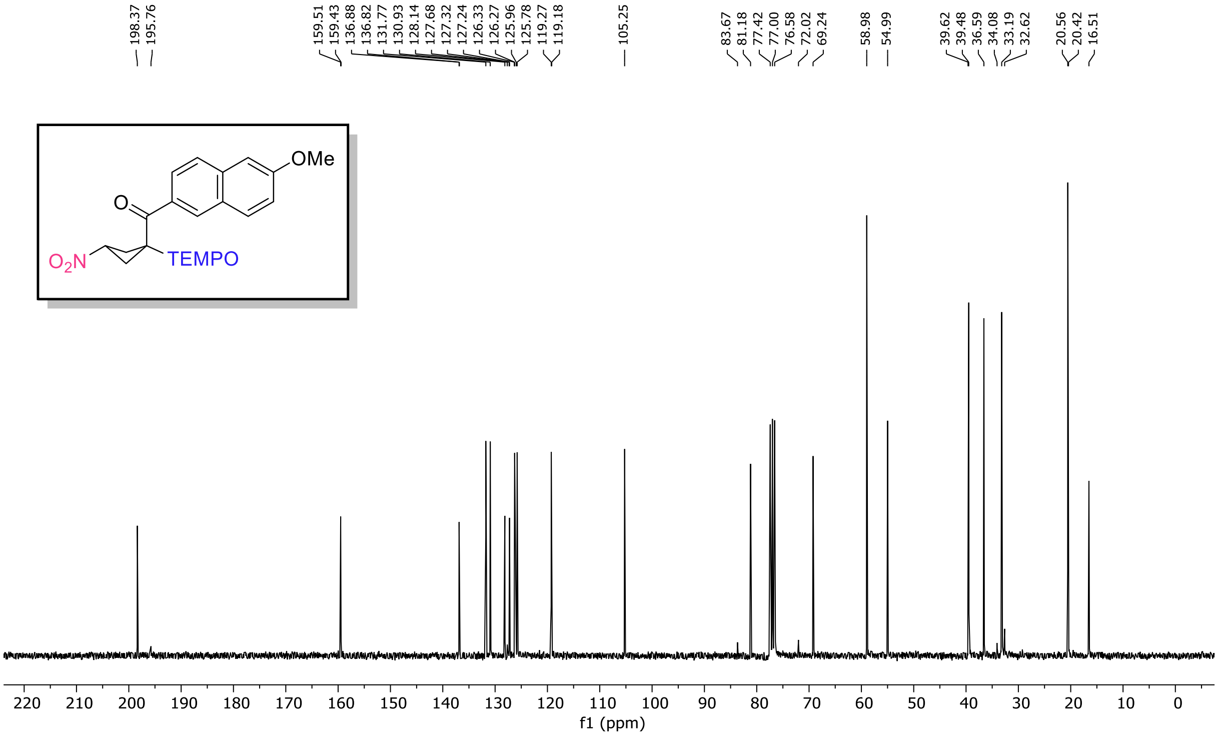

^{1}H NMR of 5 ($CDCl_3$, 300 MHz)

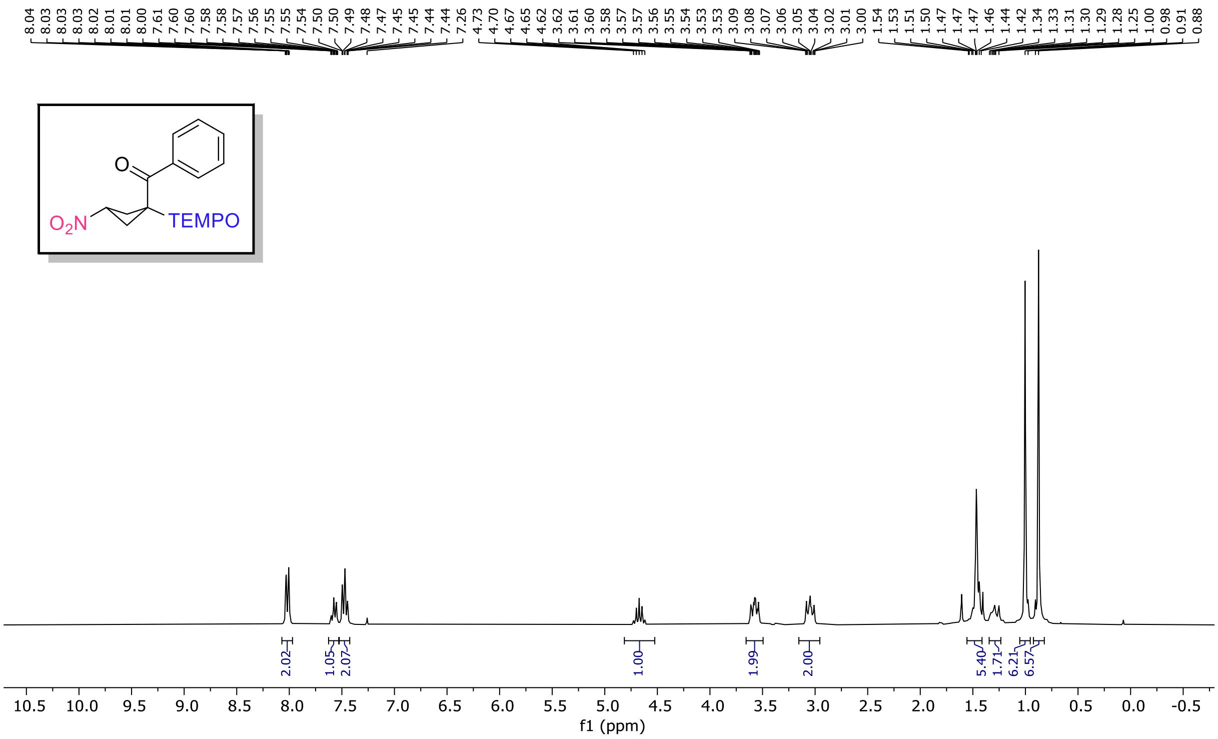

^{13}C NMR of 5 ($CDCl_3$, 76 MHz)

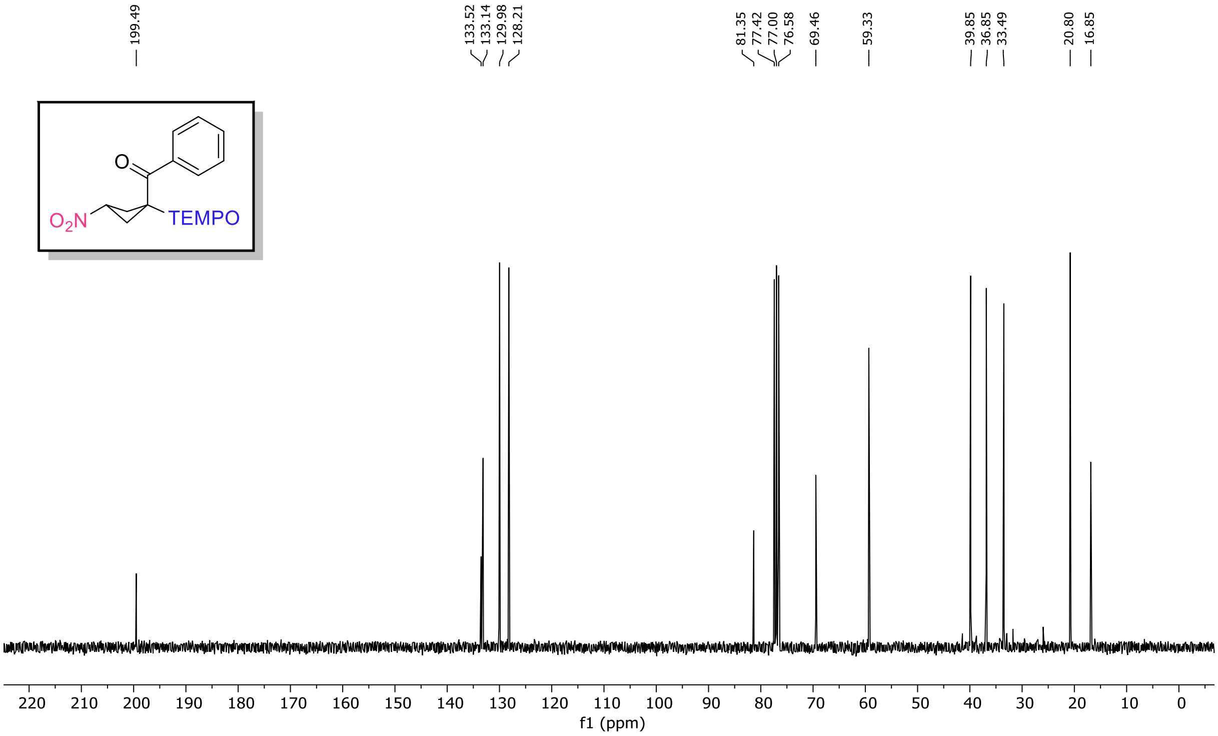

^{1}H NMR of 6 ($CDCl_3$, 300 MHz)

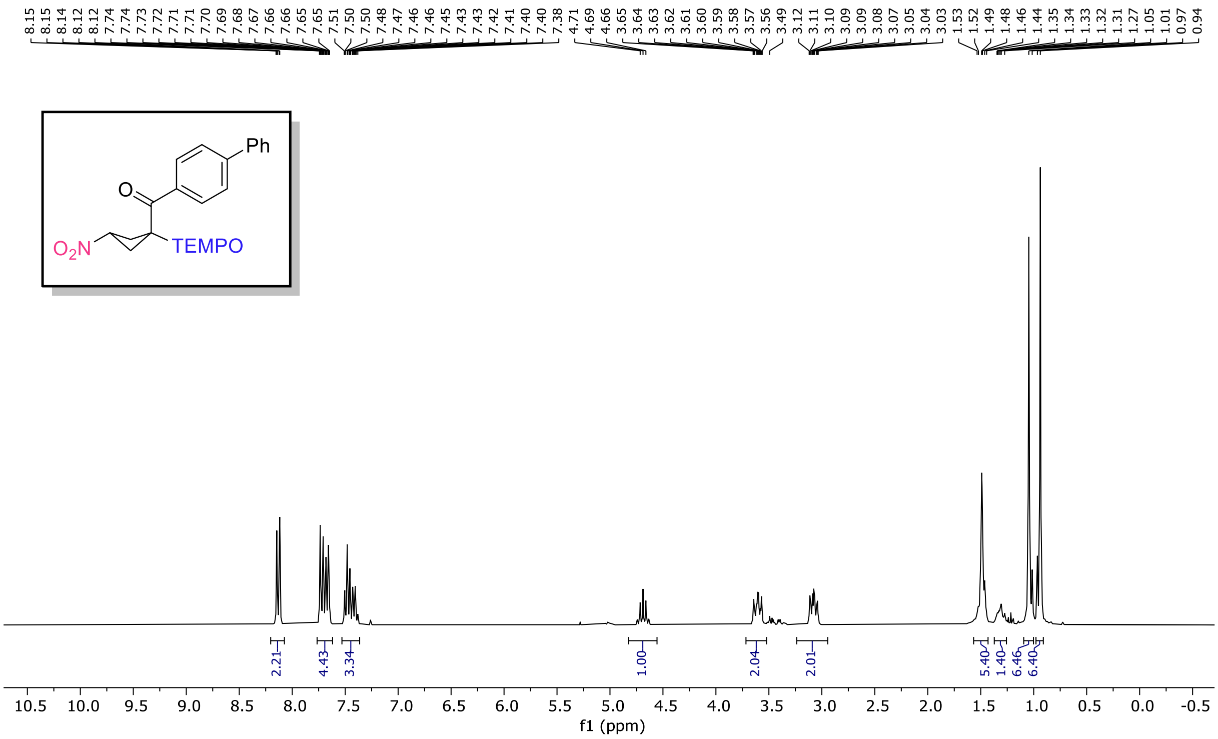

^{13}C NMR of 6 ($CDCl_3$, 76 MHz)

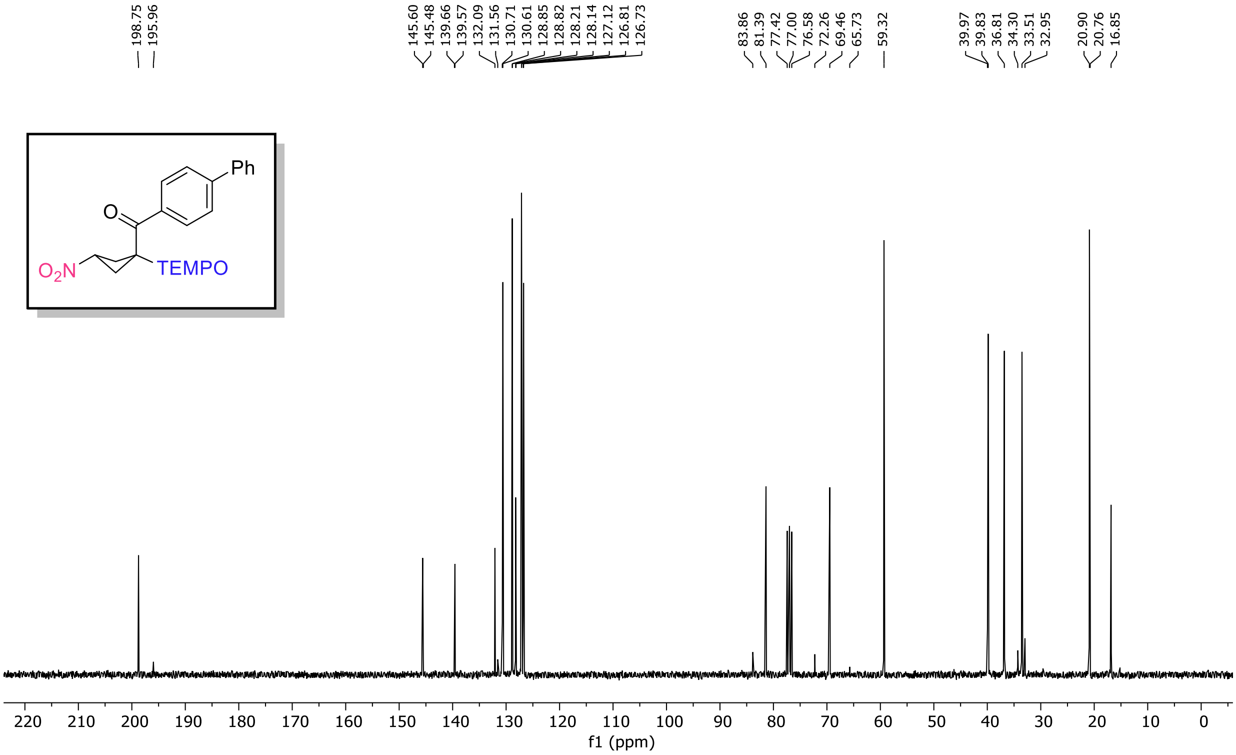

¹H NMR of 7 (CDCl₃, 300 MHz)

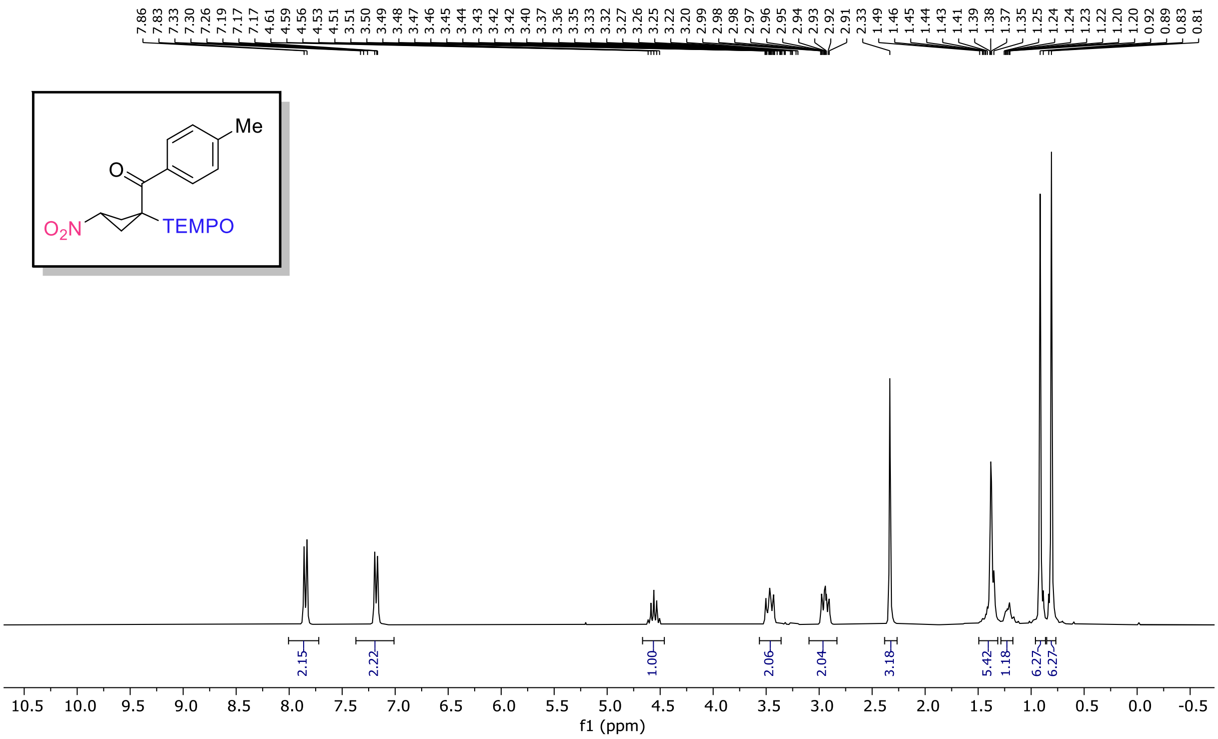

¹³C NMR of 7 (CDCl₃, 76 MHz)

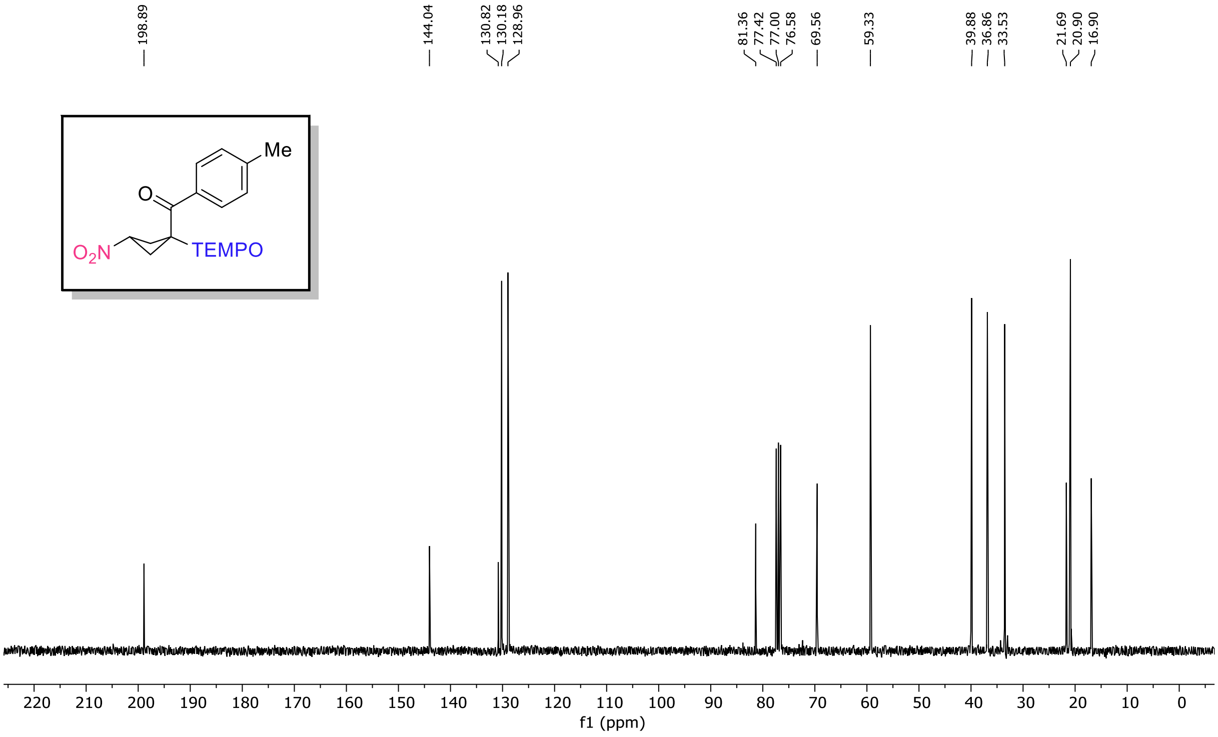

^{1}H NMR of 8 (CDCl$_3$, 300 MHz)

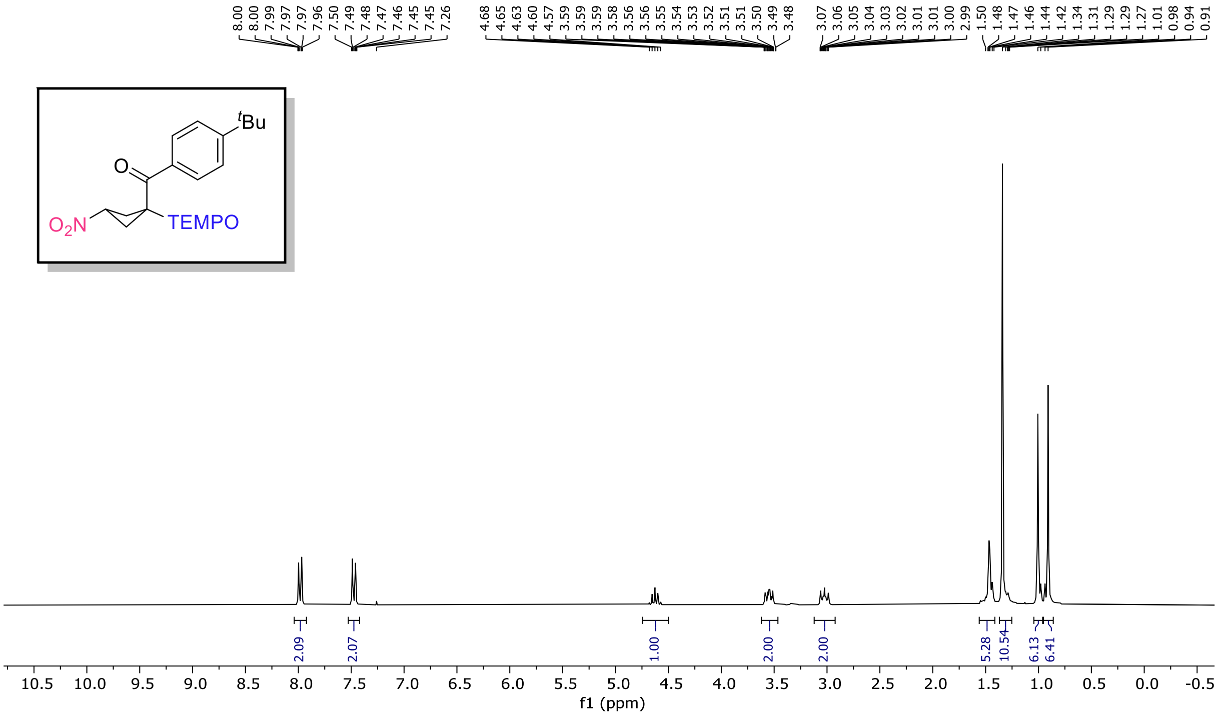

^{13}C NMR of 8 (CDCl$_3$, 76 MHz)

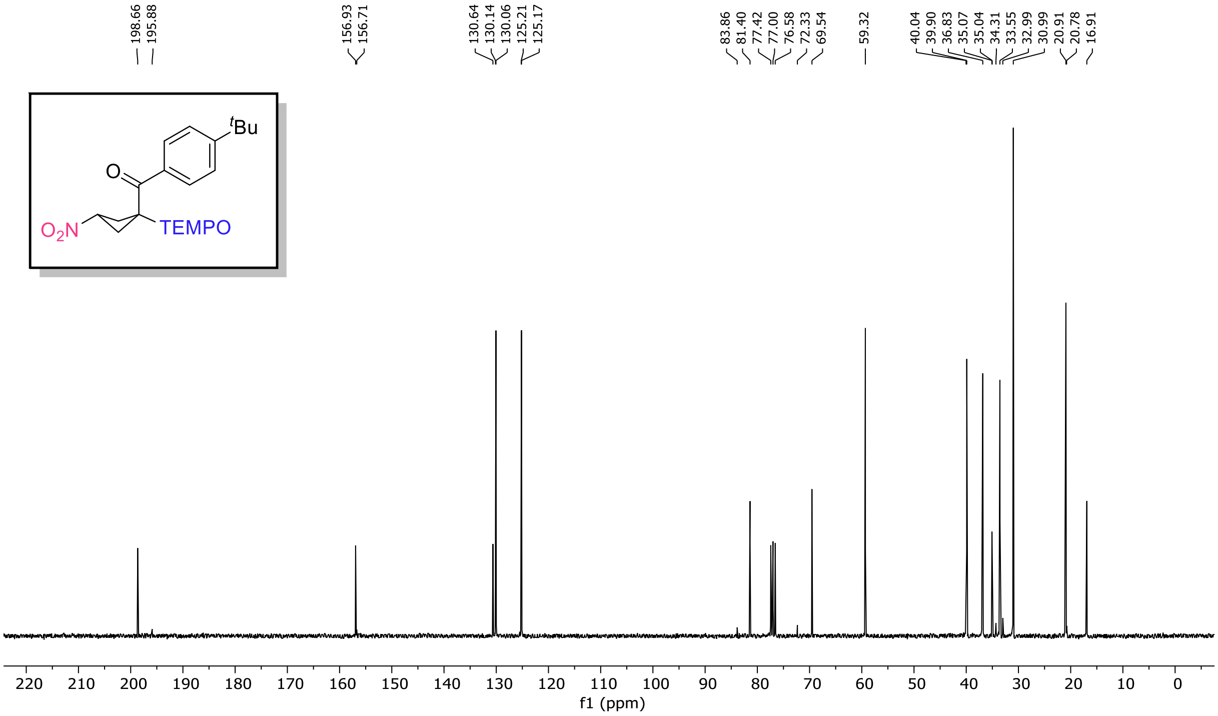

^{1}H NMR of 9 (CDCl3, 300 MHz)

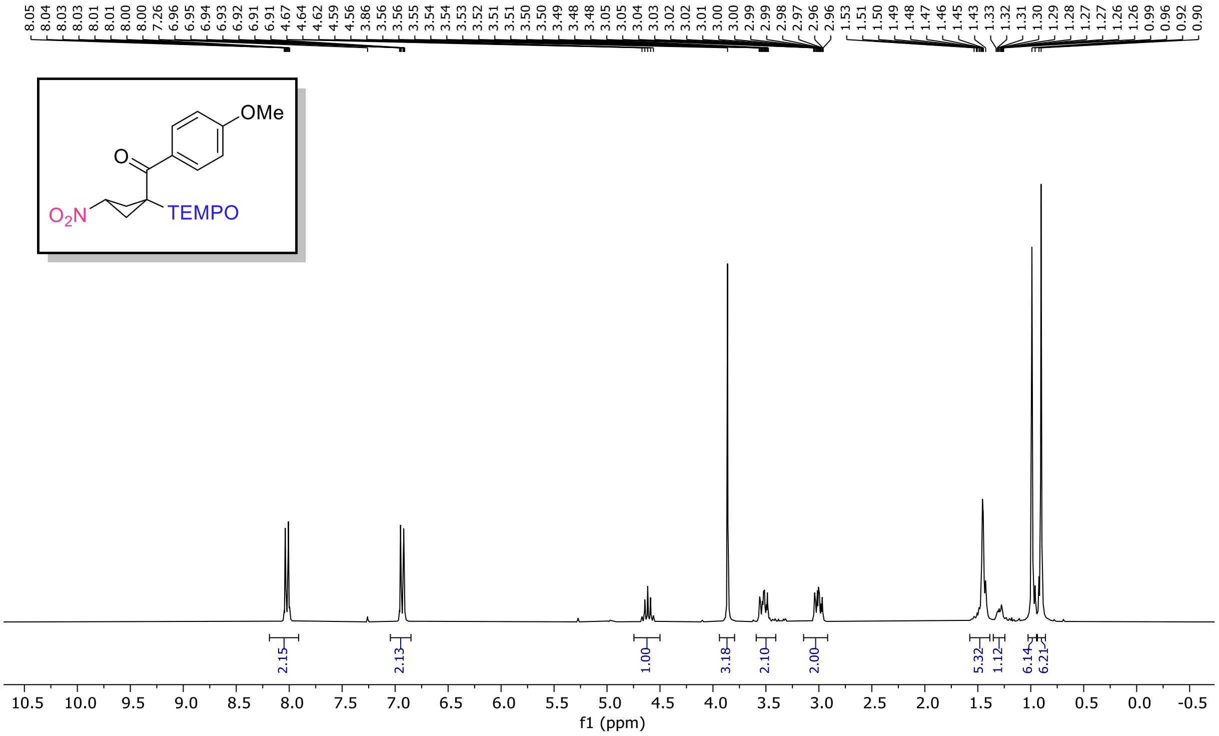

^{13}C NMR of 9 (CDCl3, 76 MHz)

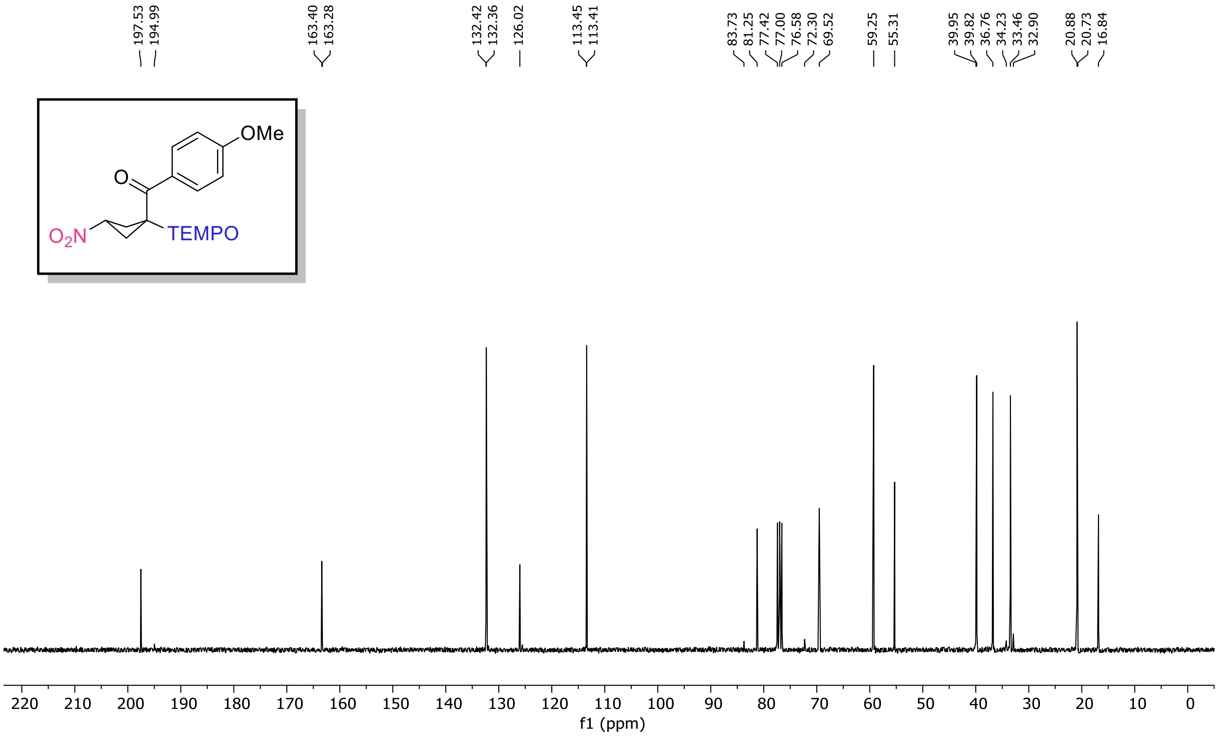

^{1}H NMR of 10 (CDCl3, 300 MHz)

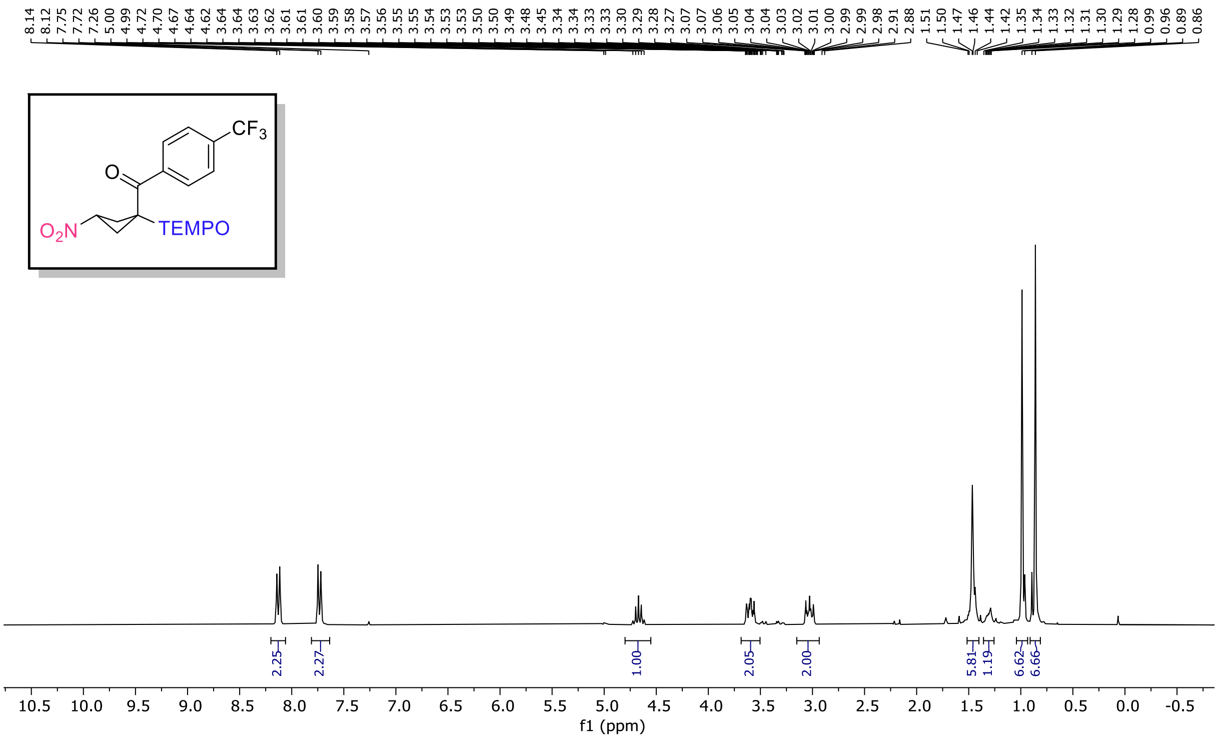

^{13}C NMR of 10 (CDCl3, 126 MHz)

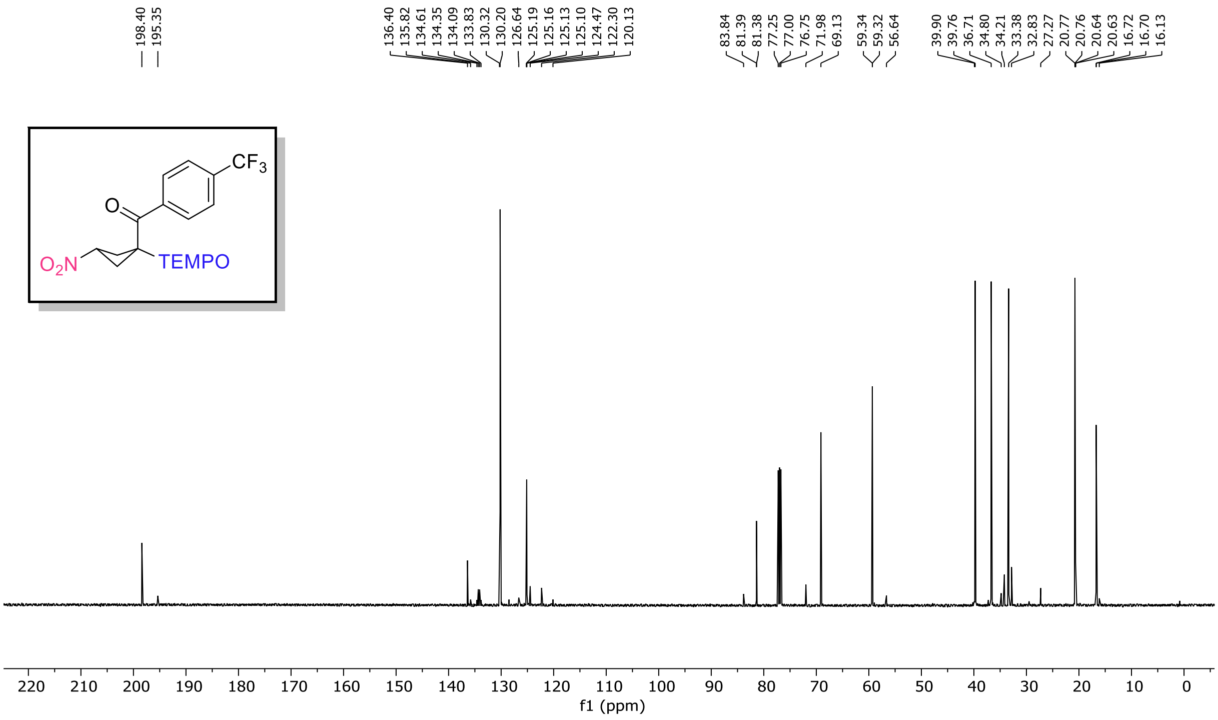

^{19}F NMR of 10 (CDCl3, 282 MHz)

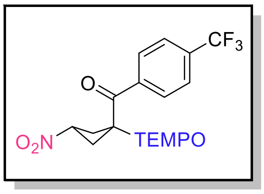

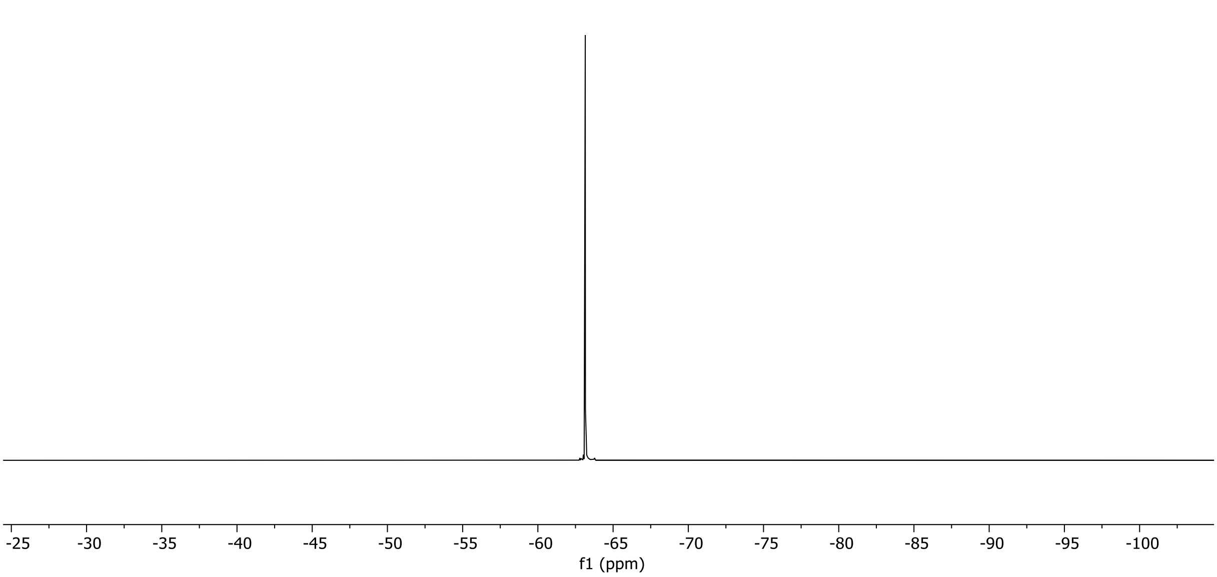

^{1}H NMR of 11 (CDCl3, 300 MHz)

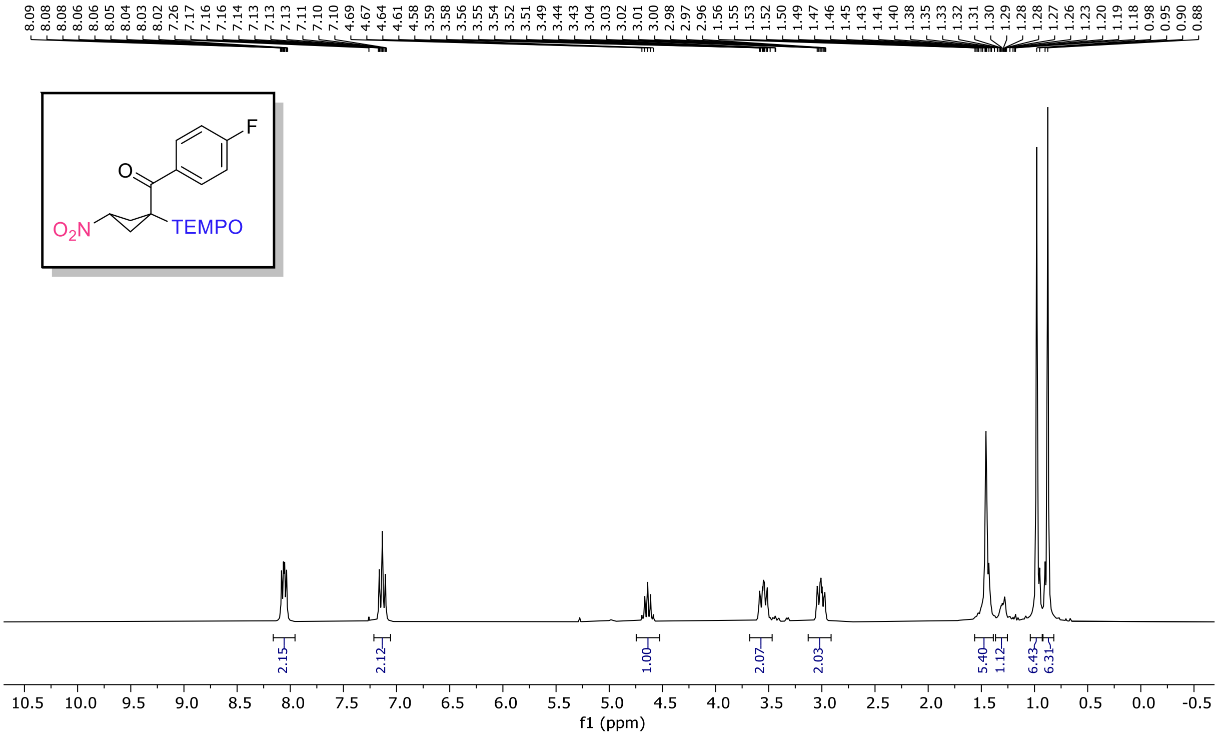

^{13}C NMR of 11 (CDCl3, 76 MHz)

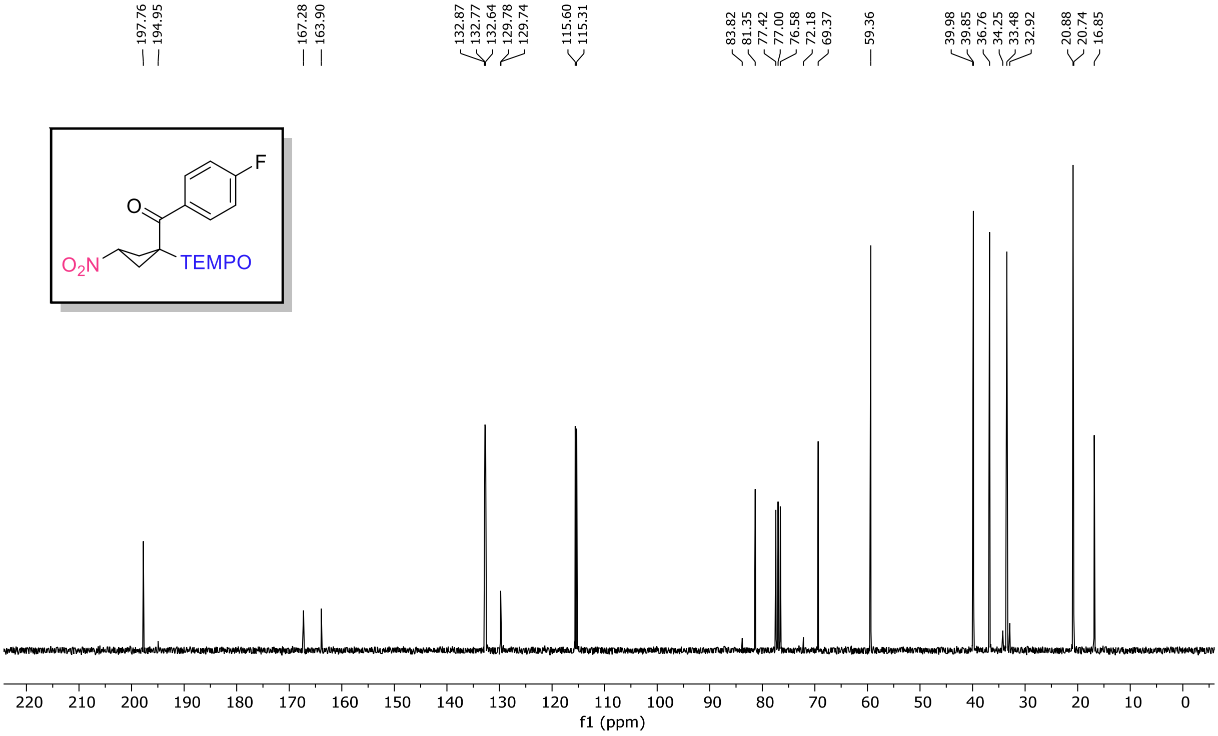

^{19}F NMR of 11 ($CDCl_3$, 282 MHz)

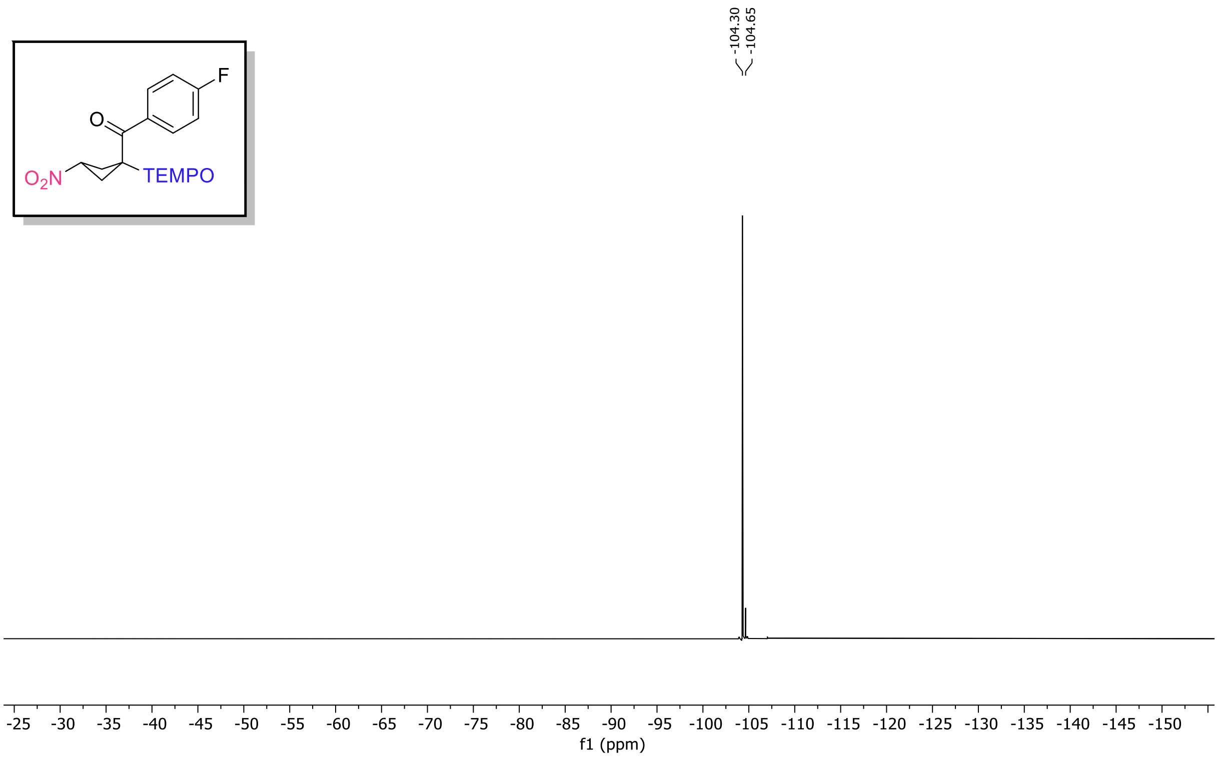

^{1}H NMR of 12 (CDCl$_3$, 300 MHz)

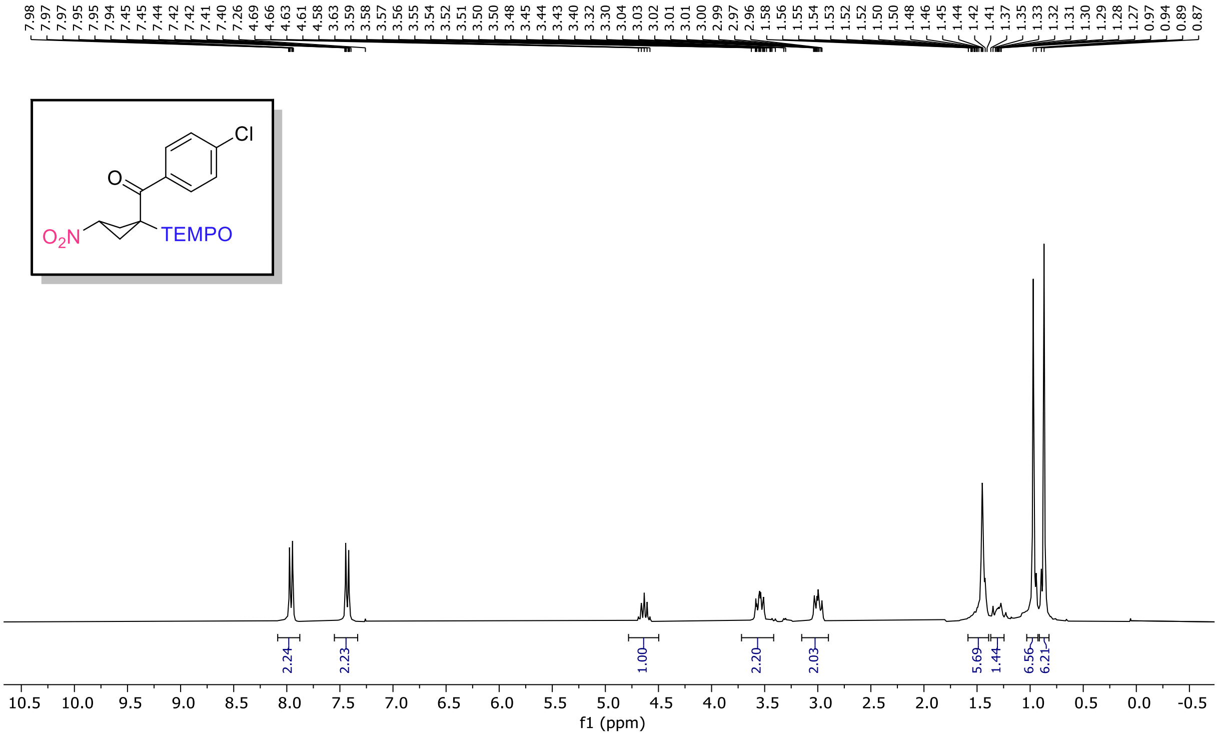

^{13}C NMR of 12 (CDCl$_3$, 76 MHz)

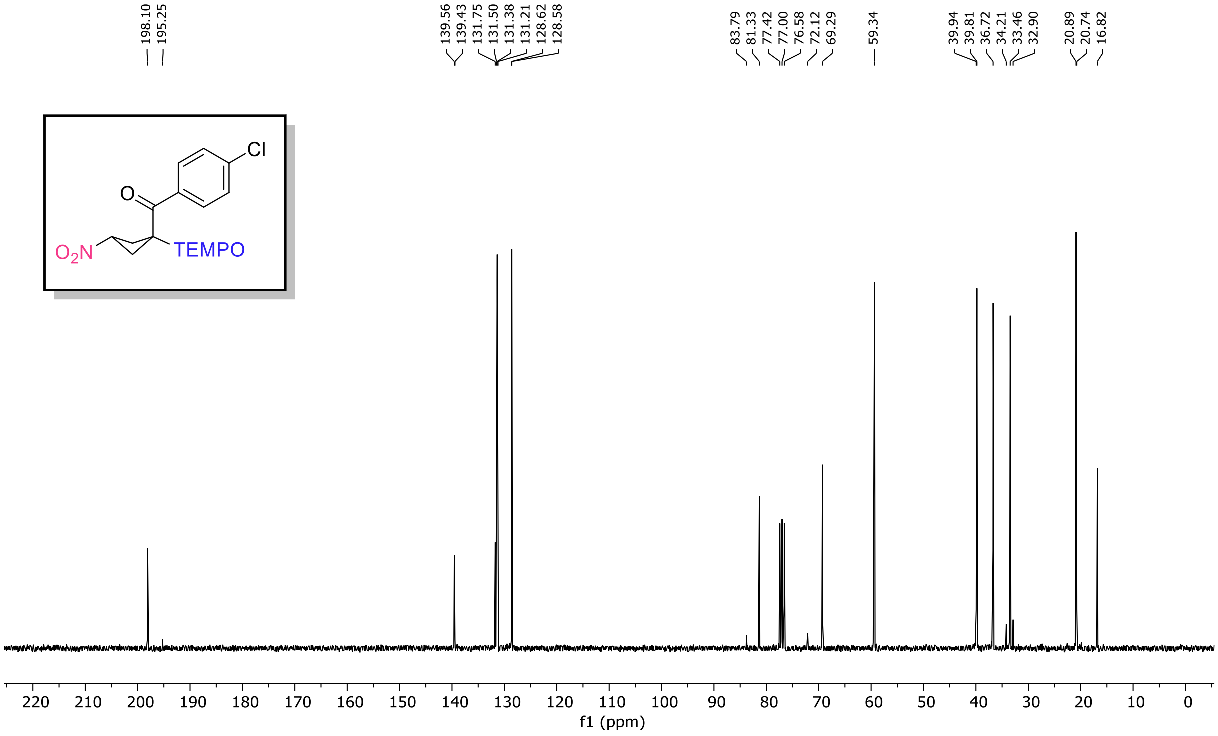

^{1}H NMR of 13 (CDCl$_3$, 300 MHz)

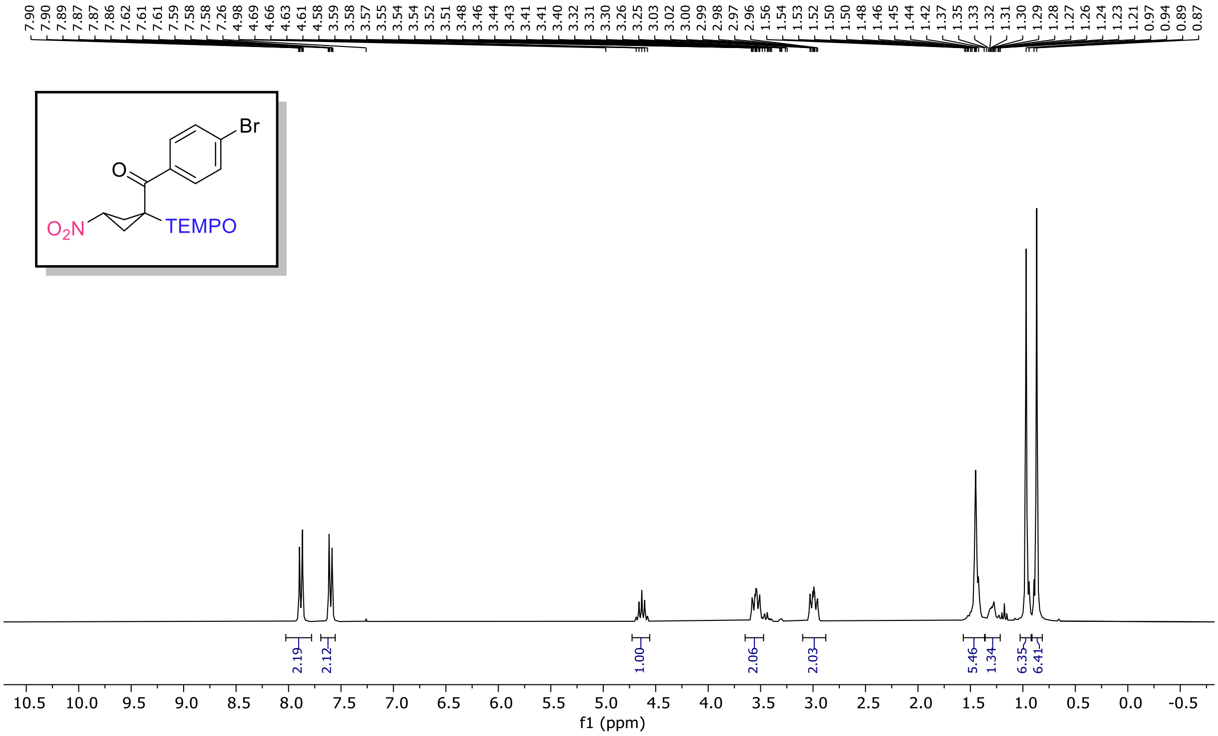

^{13}C NMR of 13 (CDCl$_3$, 76 MHz)

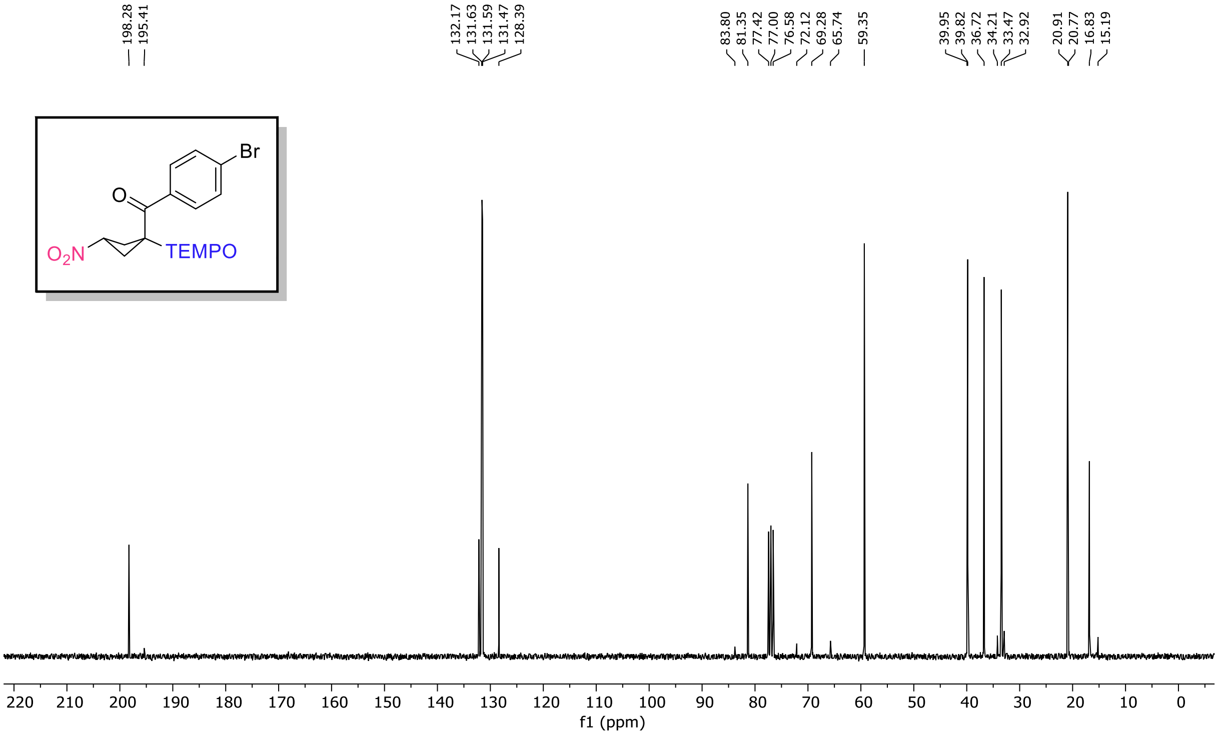

^{1}H NMR of 14 (CDCl$_3$, 300 MHz)

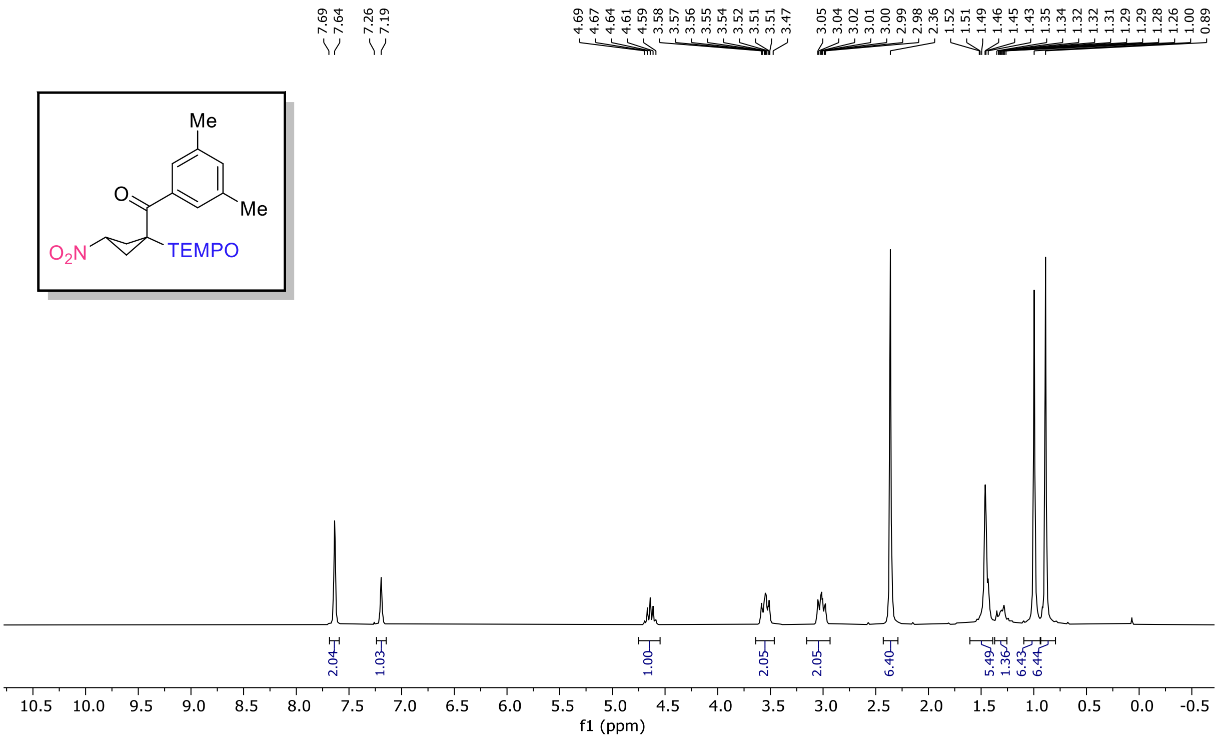

^{13}C NMR of 14 (CDCl$_3$, 76 MHz)

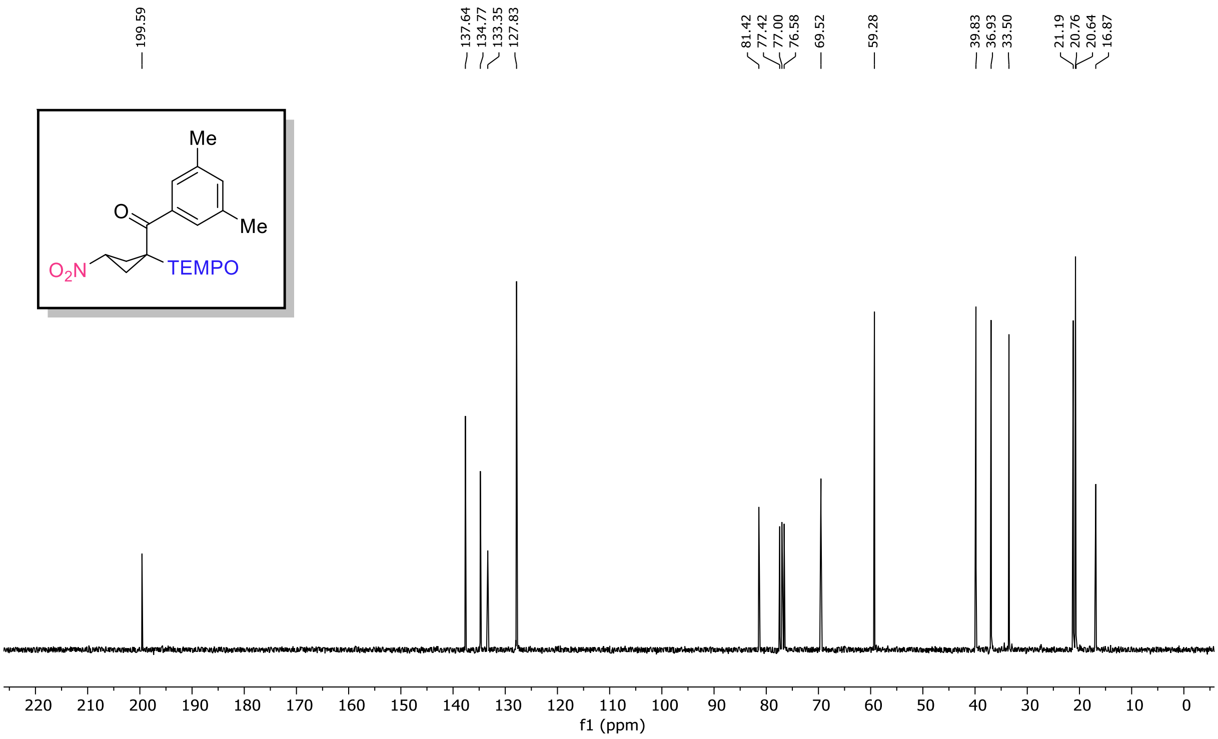

^{1}H NMR of 15 (CDCl$_3$, 300 MHz)

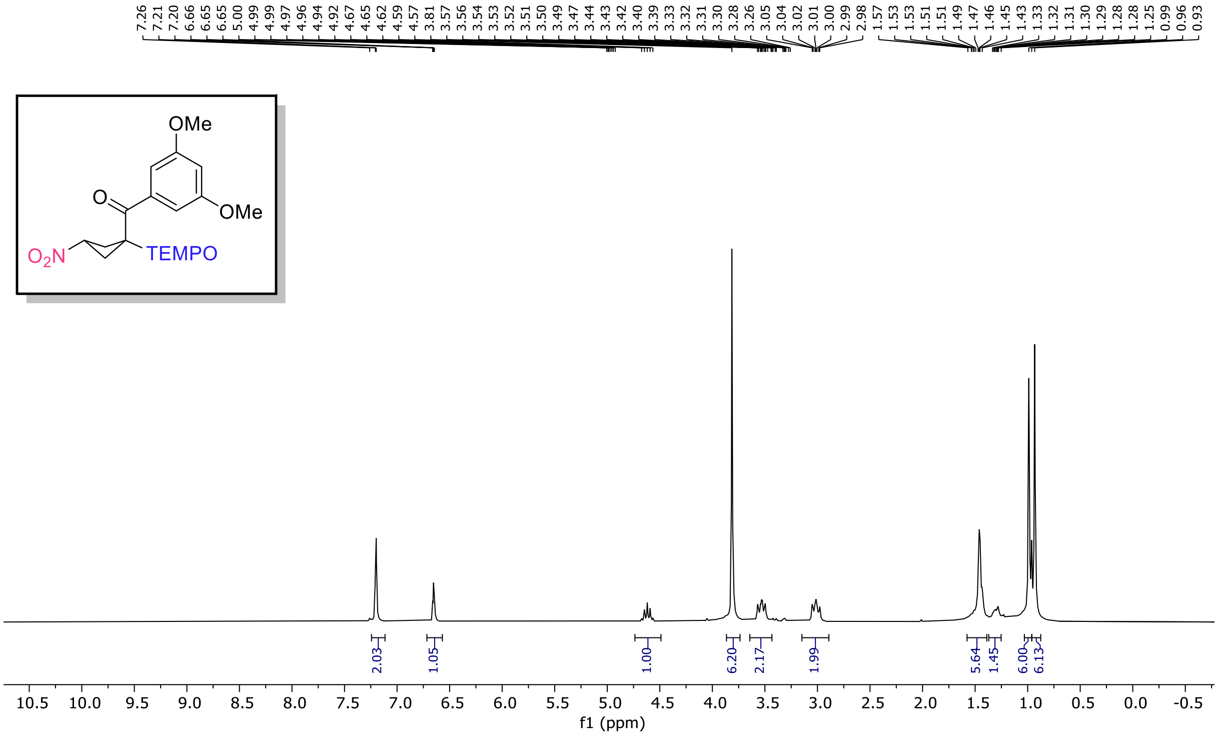

^{13}C NMR of 15 (CDCl$_3$, 76 MHz)

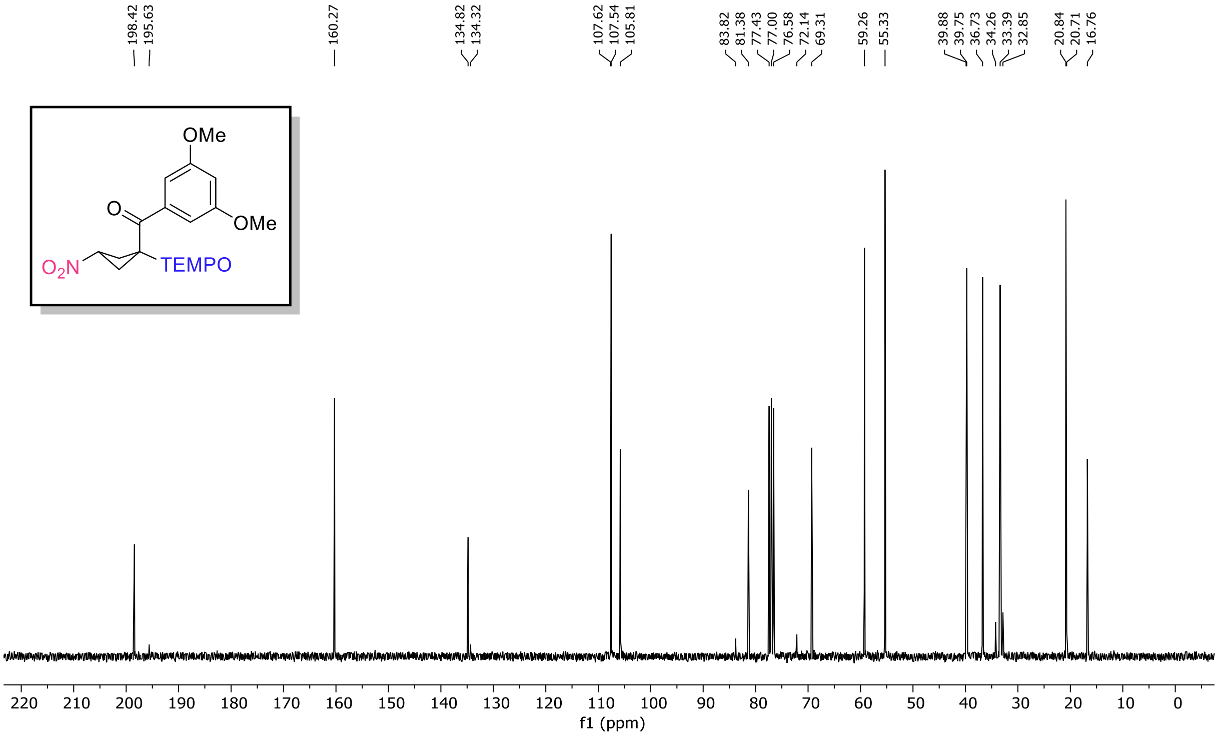

^{1}H NMR of 16 ($CDCl_3$, 300 MHz)

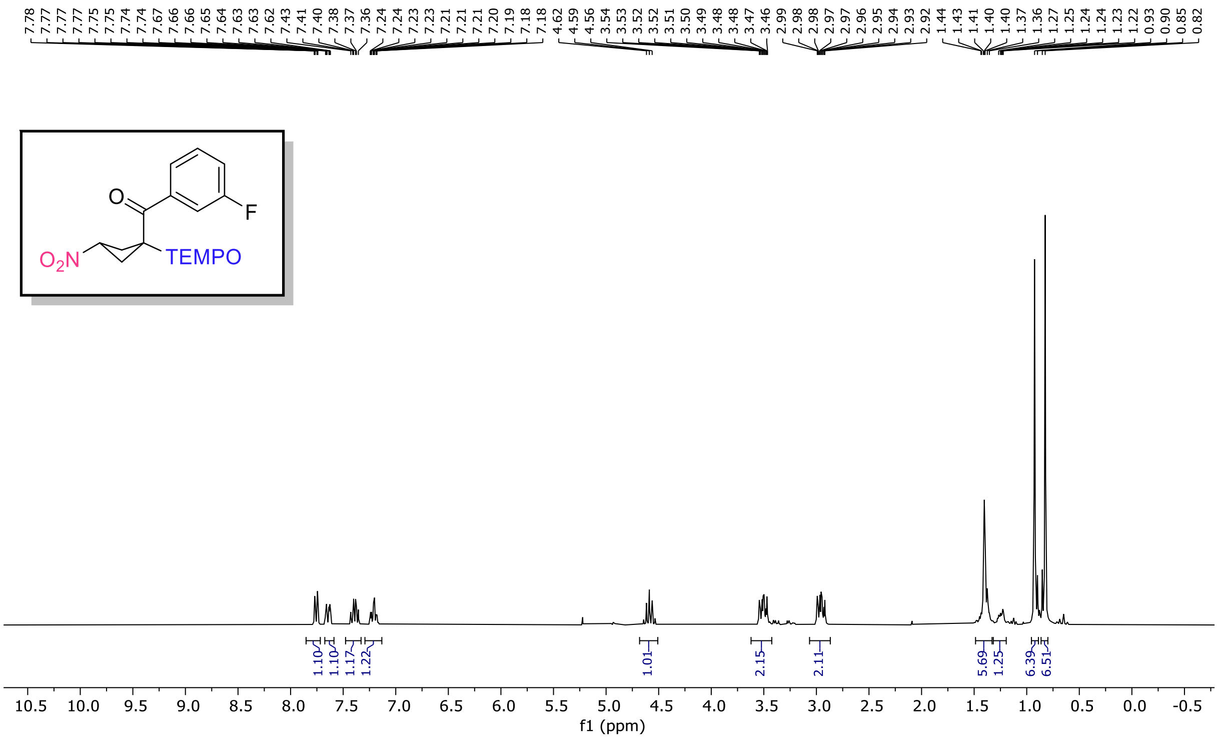

^{13}C NMR of 16 ($CDCl_3$, 76 MHz)

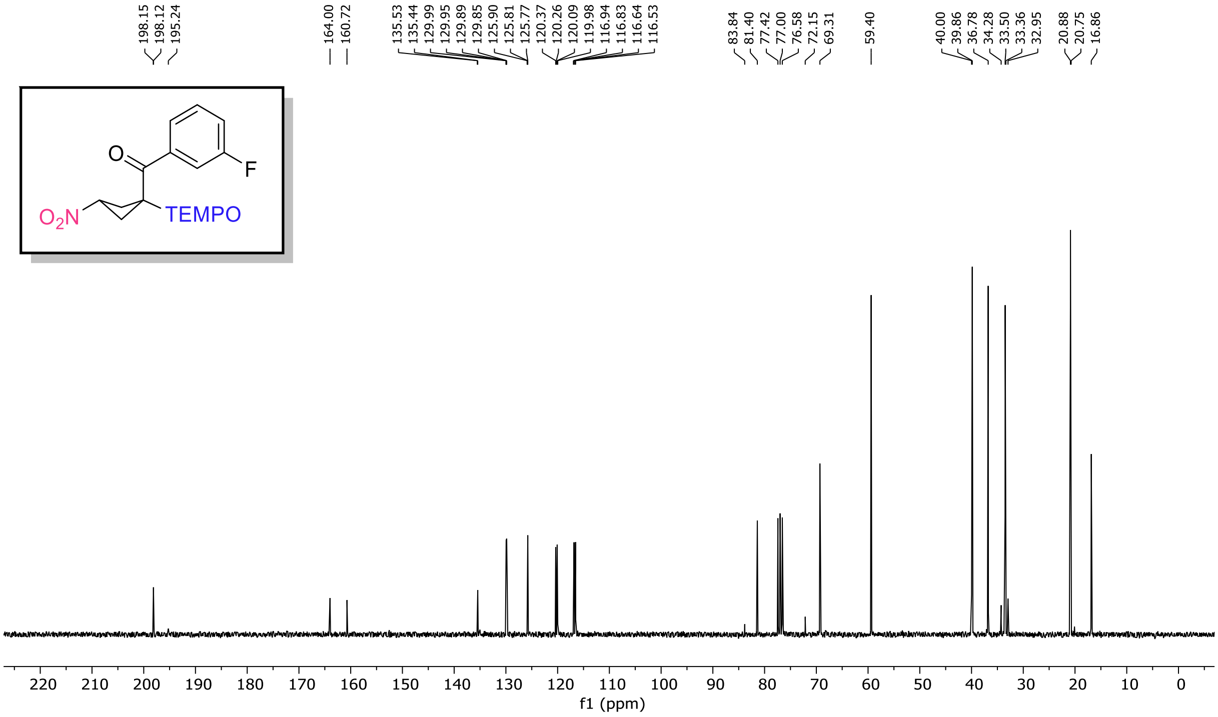

^{19}F NMR of 16 ($CDCl_3$, 282 MHz)

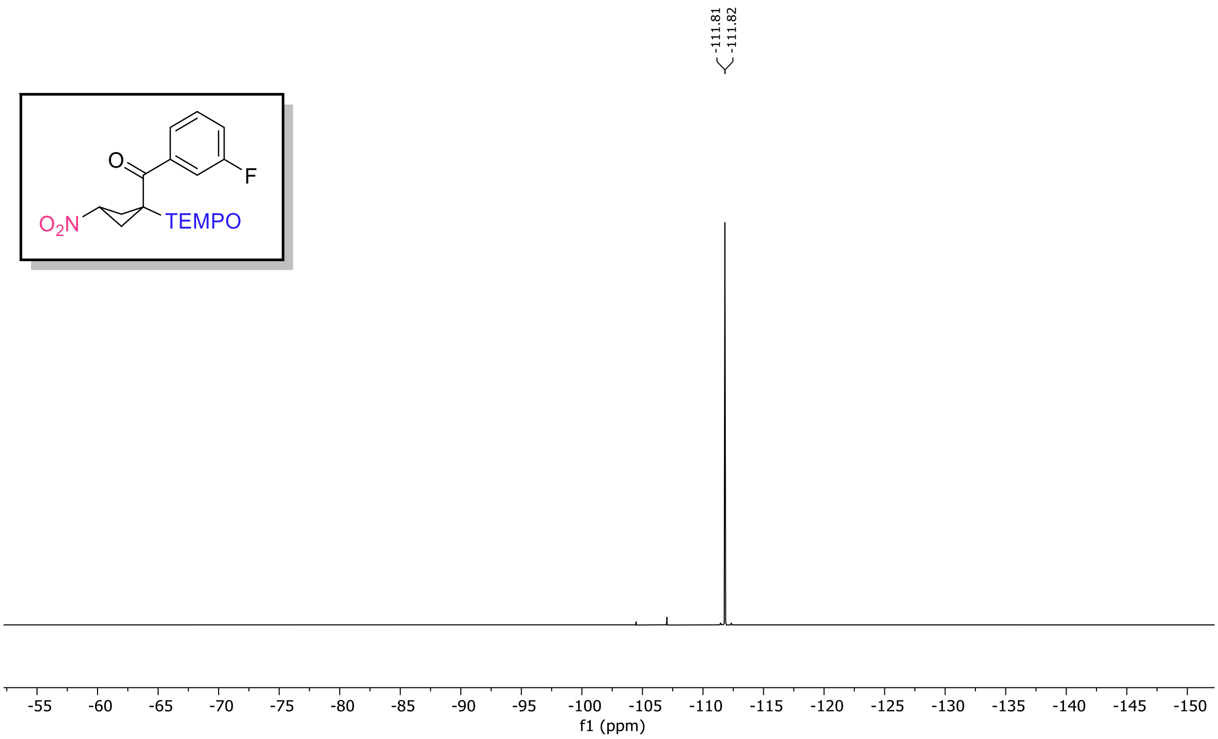

¹H NMR of 17 (CDCl₃, 300 MHz)

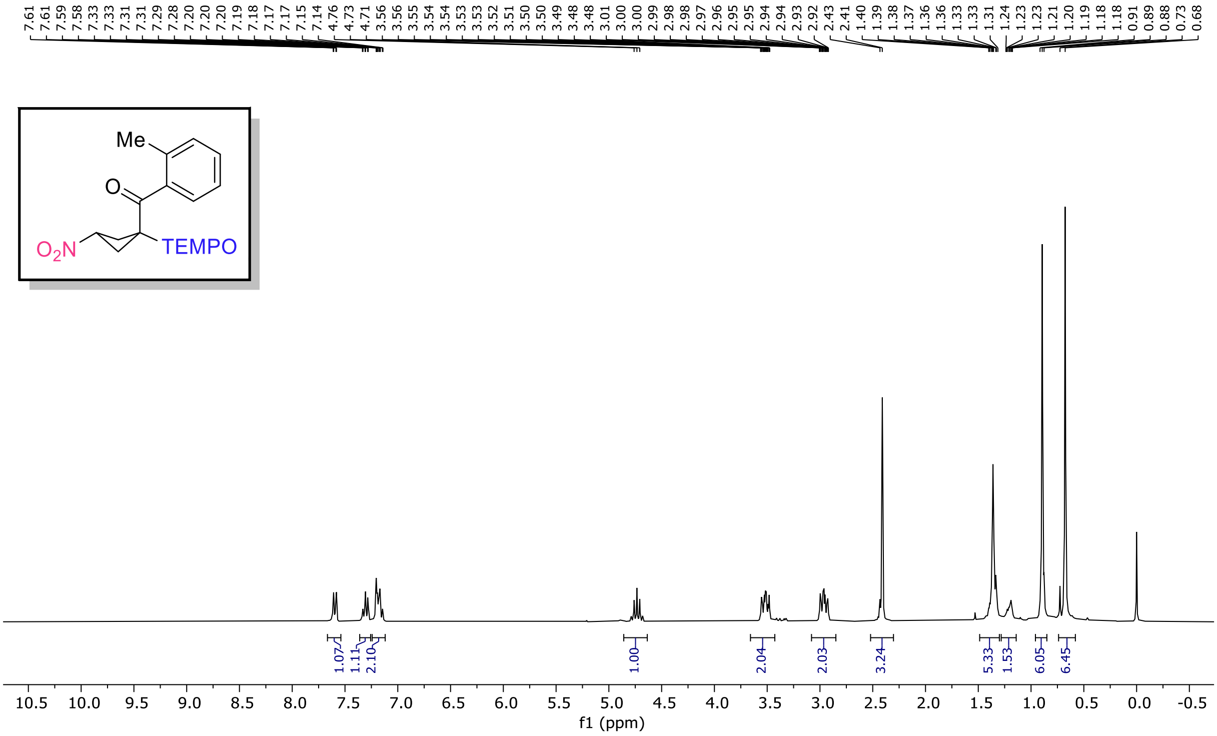

¹³C NMR of 17 (CDCl₃, 76 MHz)

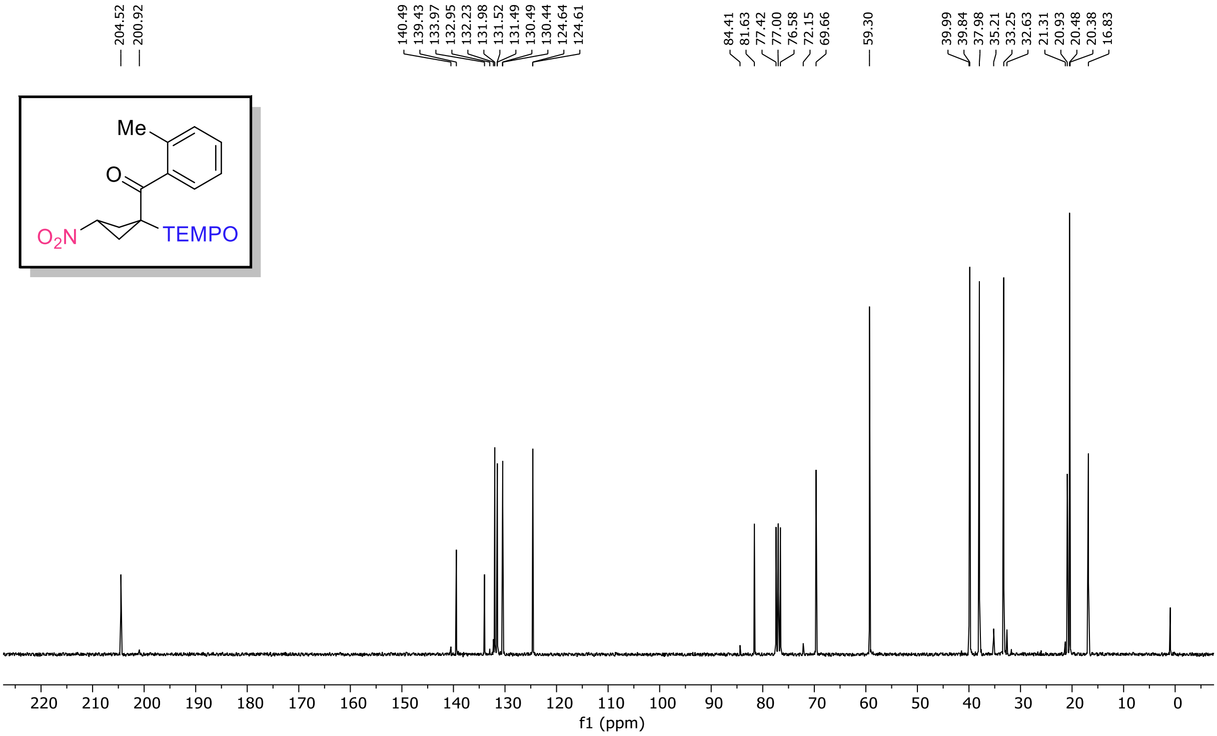

^{1}H NMR of 18 (CDCl3, 300 MHz)

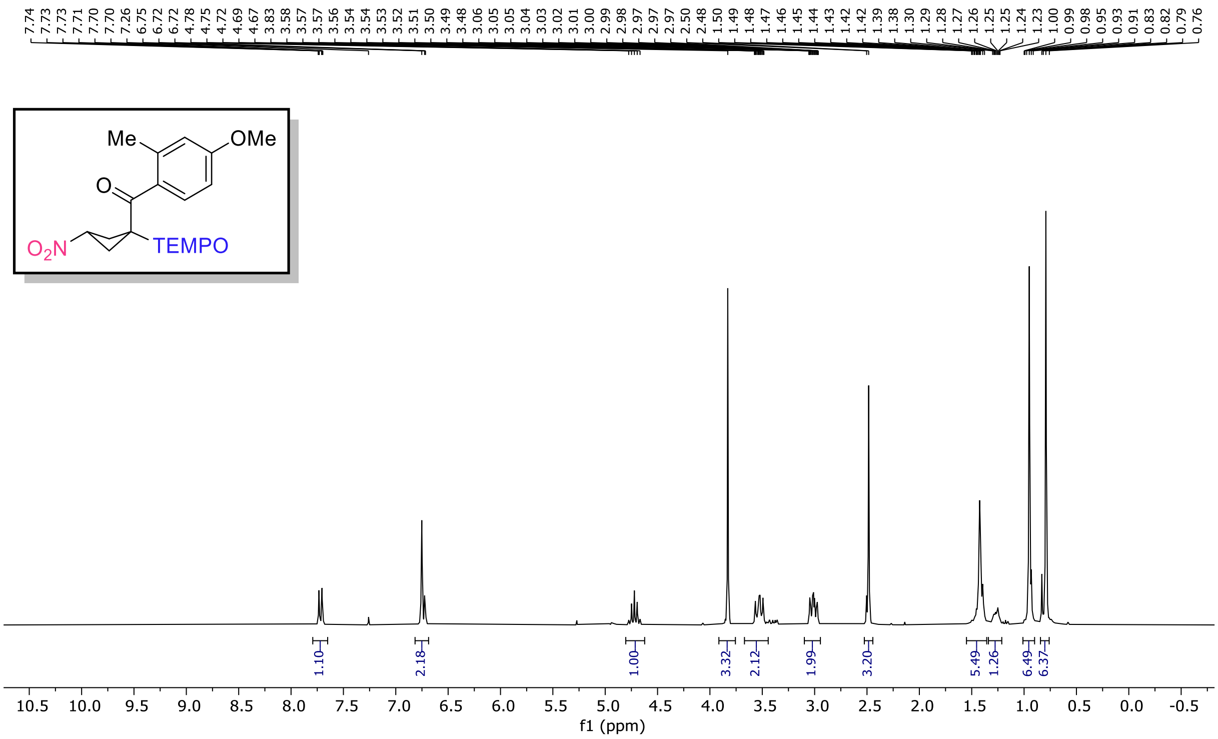

^{13}C NMR of 18 (CDCl3, 76 MHz)

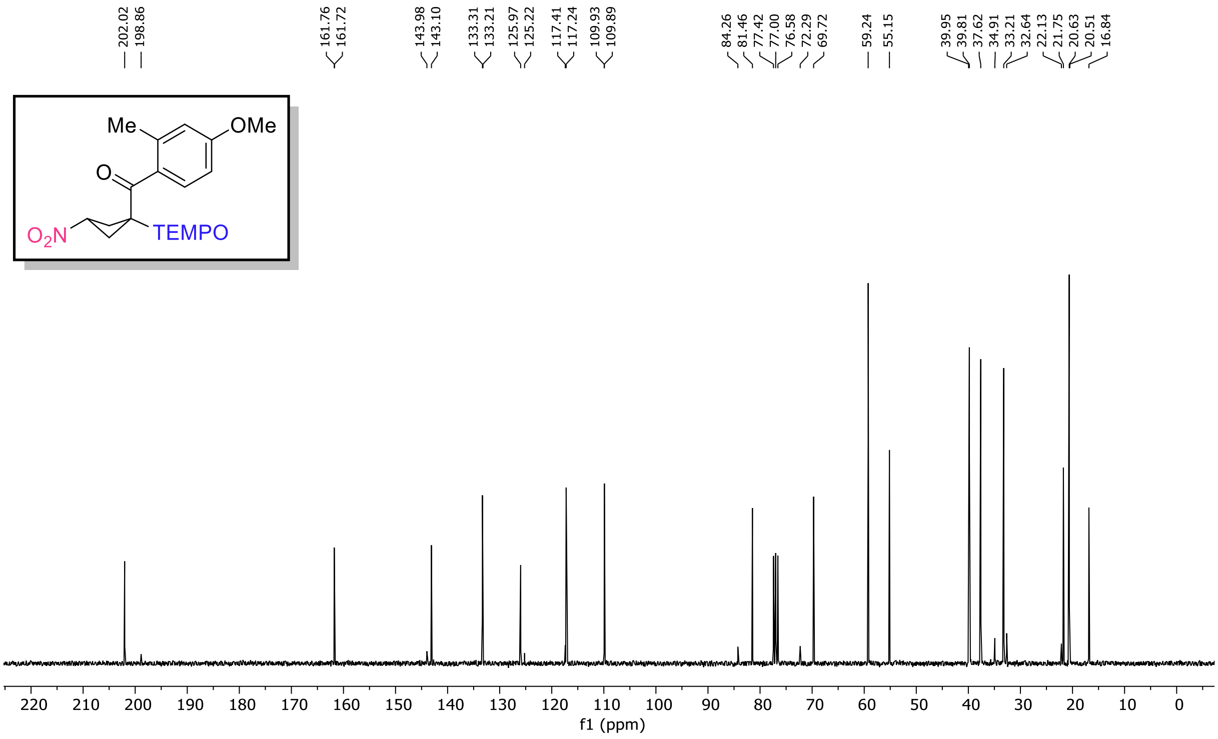

^{1}H NMR of 19 (CDCl$_3$, 300 MHz)

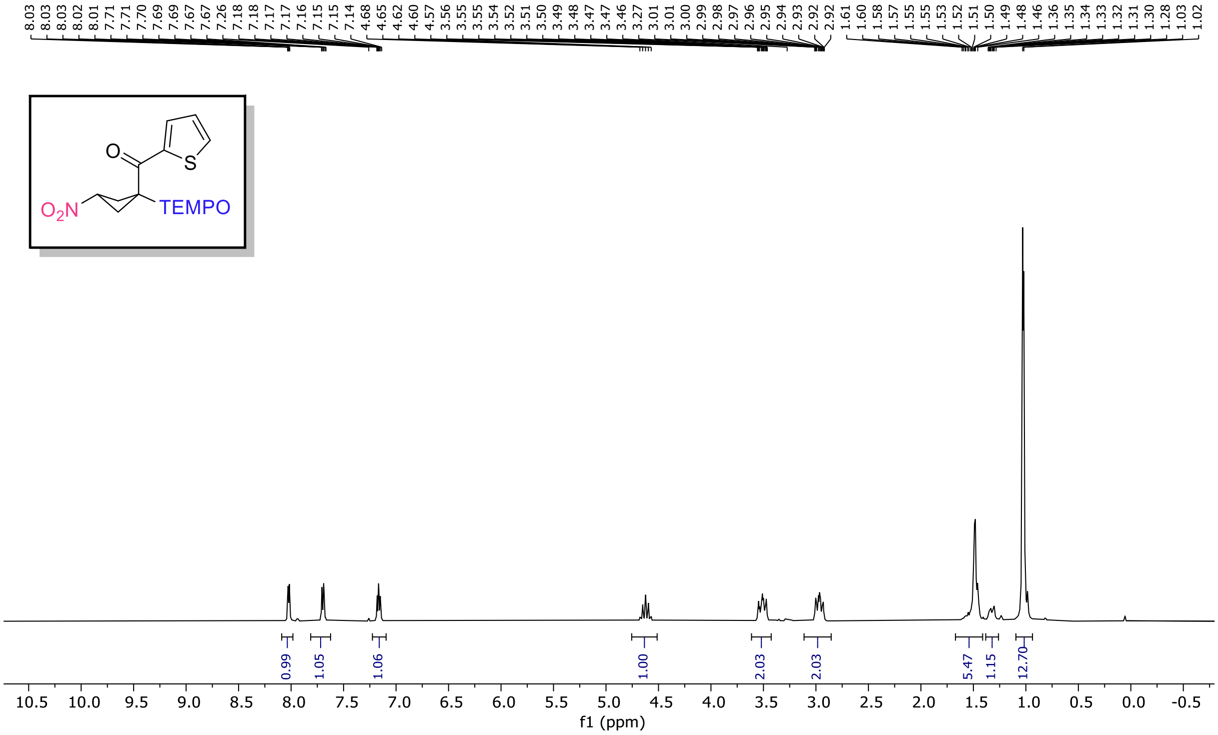

^{13}C NMR of 19 (CDCl$_3$, 76 MHz)

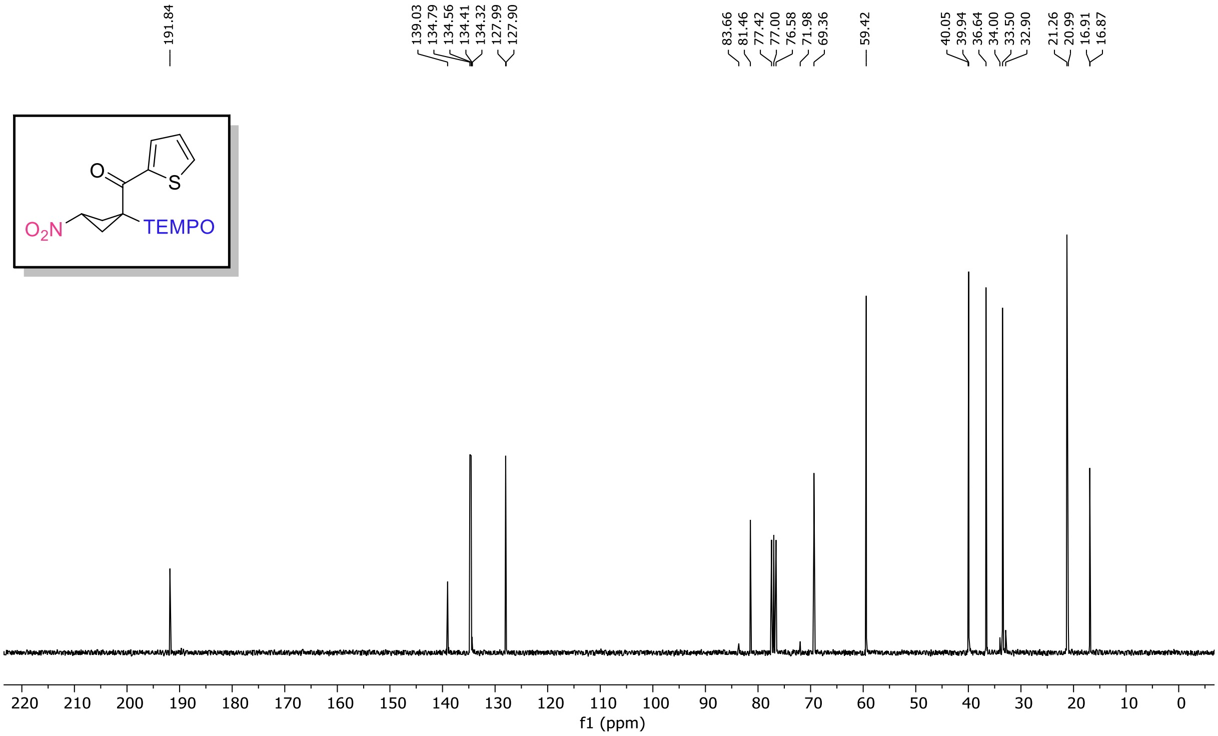

^{1}H NMR of 20 (CDCl$_3$, 300 MHz)

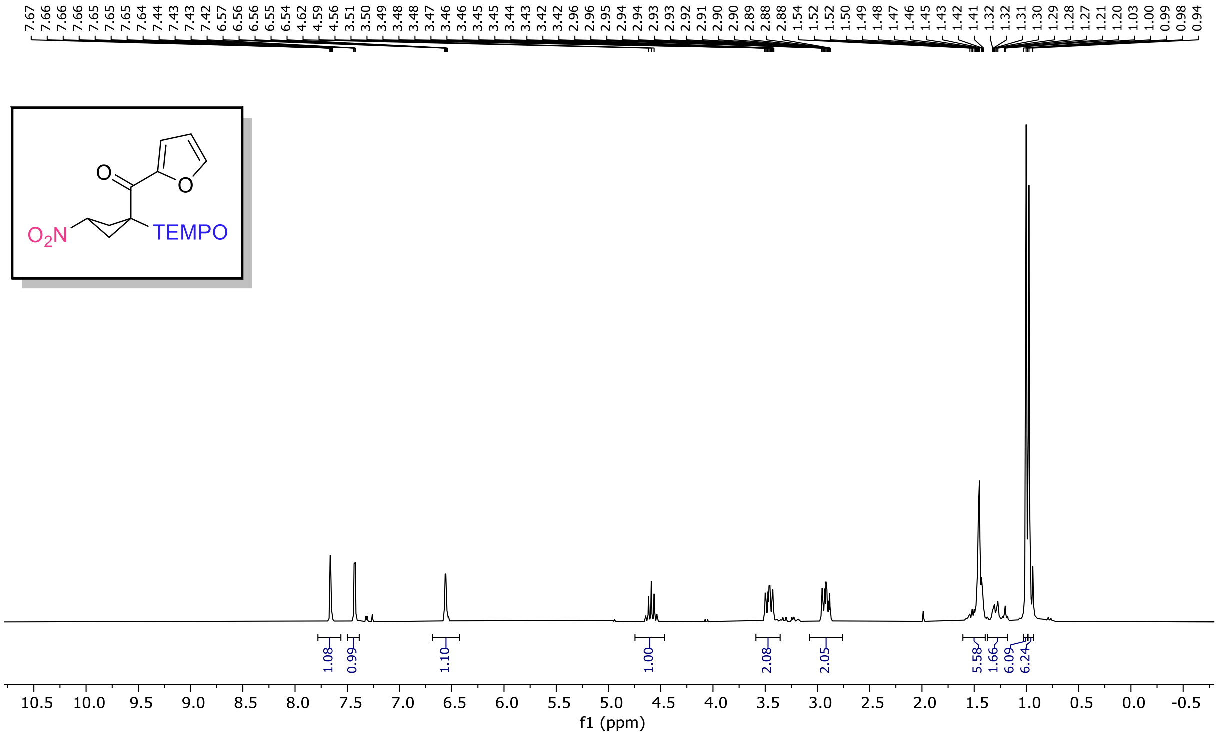

^{13}C NMR of 20 (CDCl$_3$, 76 MHz)

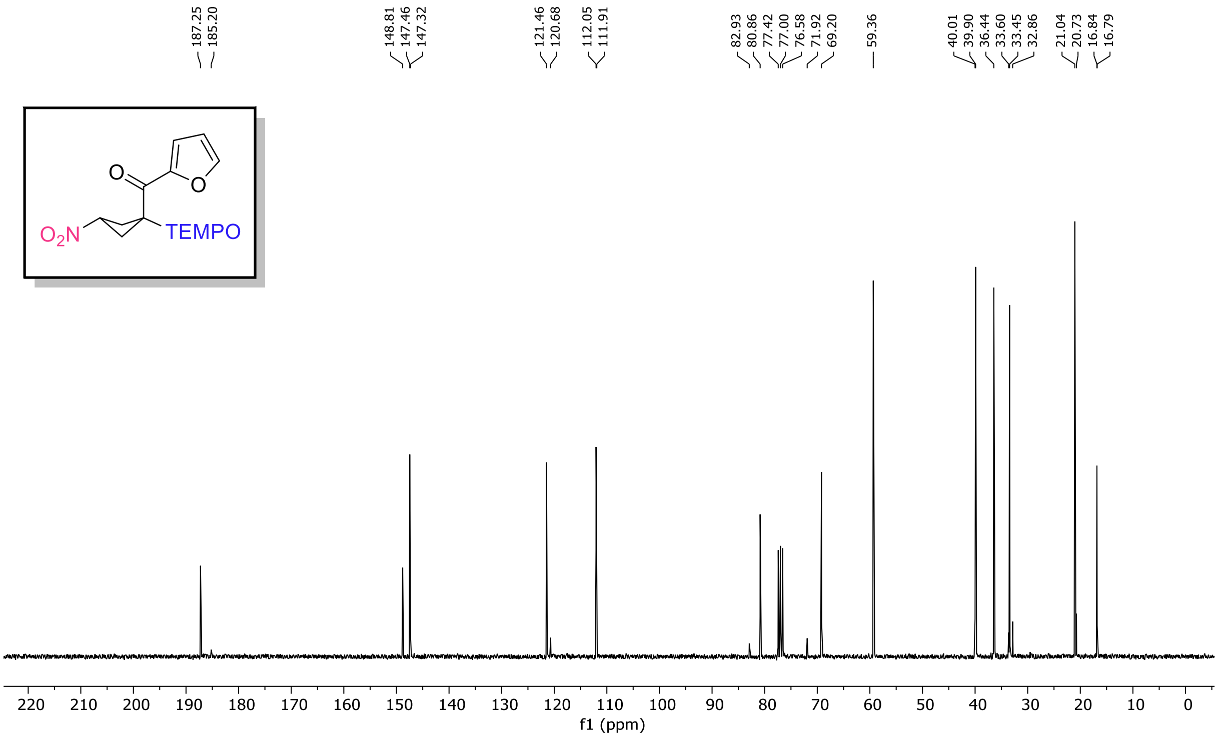

^{1}H NMR of 22 ($CDCl_3$, 599 MHz)

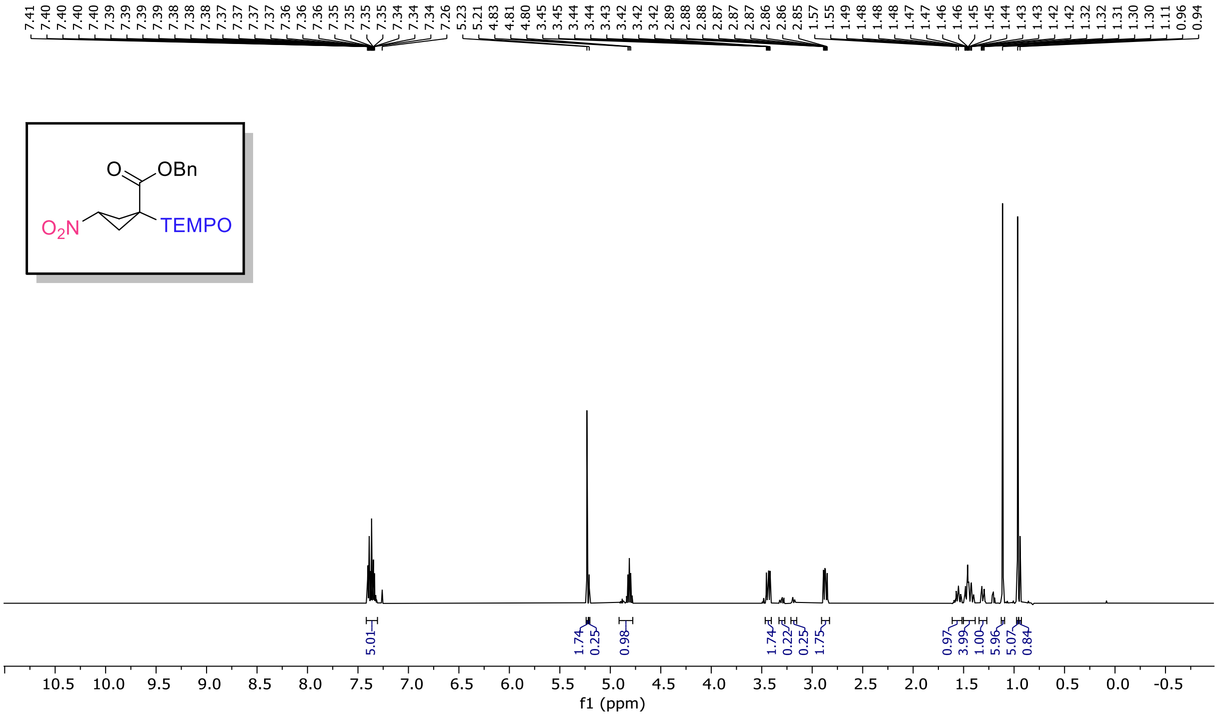

^{13}C NMR of 22 ($CDCl_3$, 151 MHz)

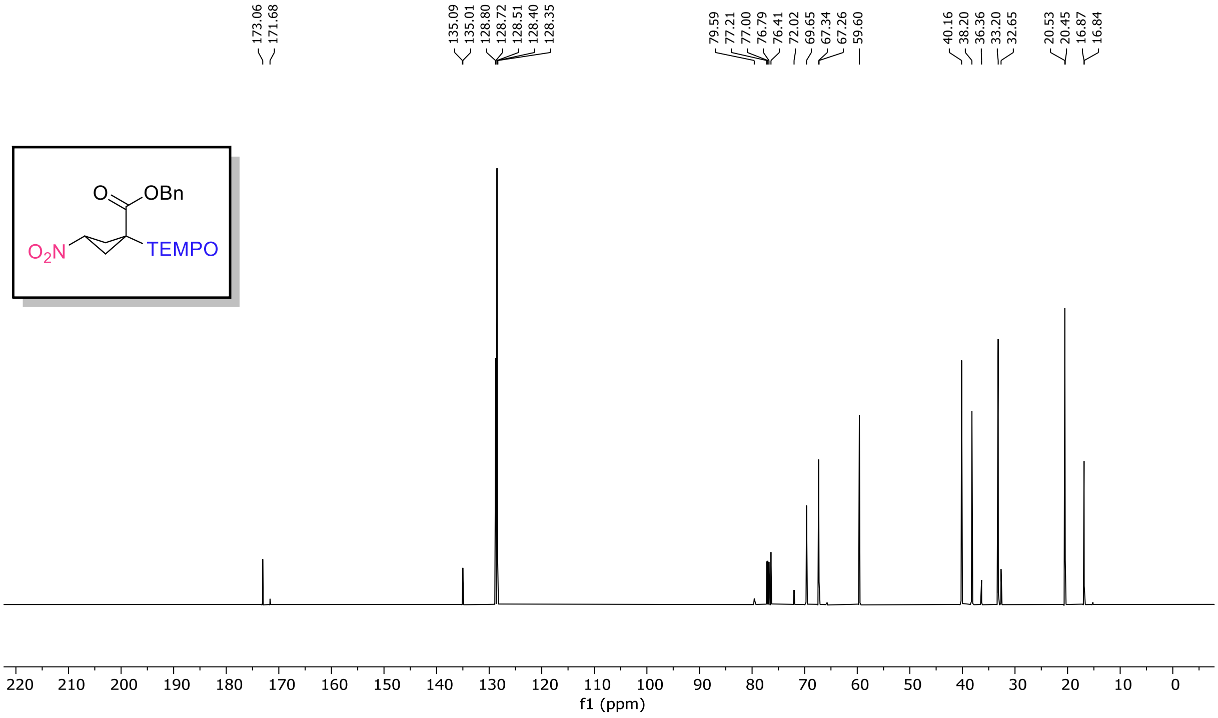

^{1}H NMR of 23 ($CDCl_3$, 400 MHz)

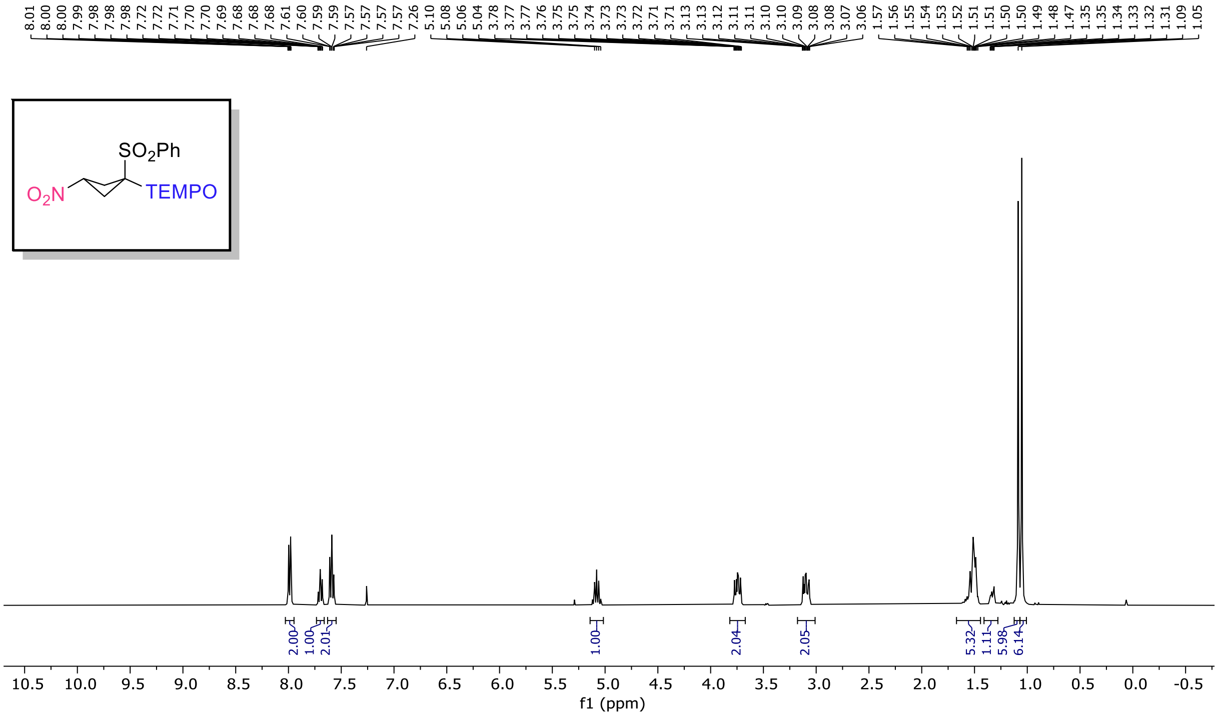

^{13}C NMR of 23 ($CDCl_3$, 101 MHz)

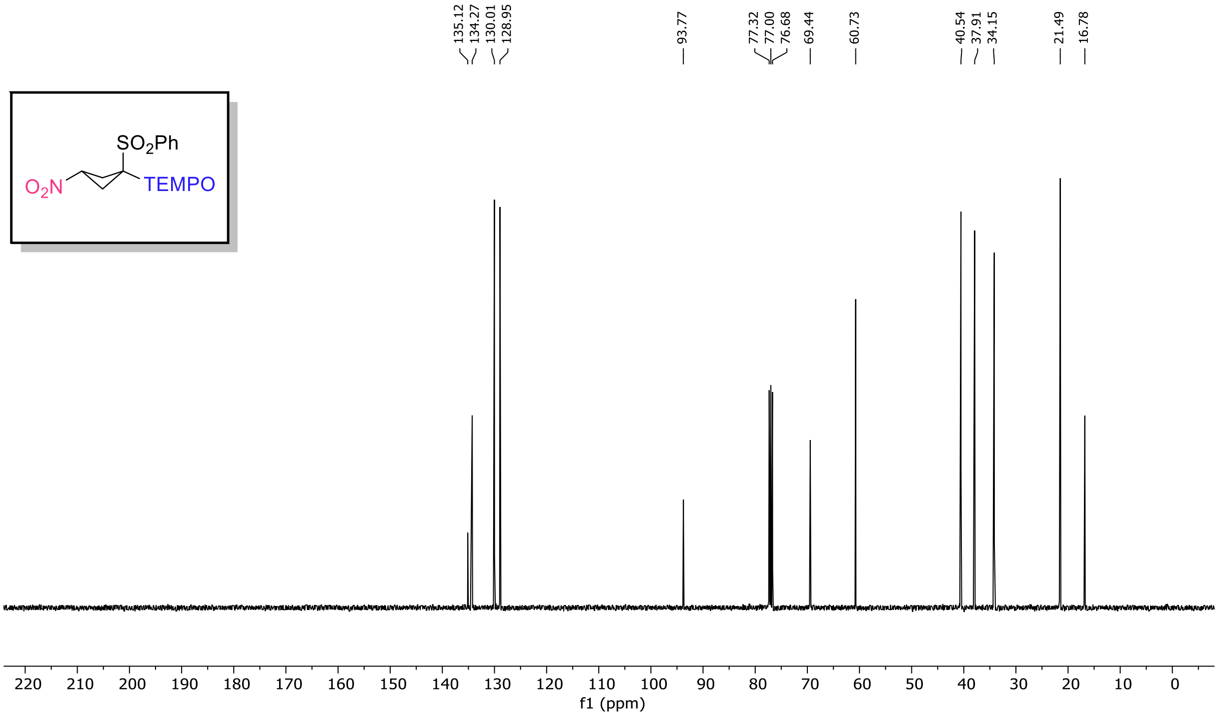

^{1}H NMR of 24

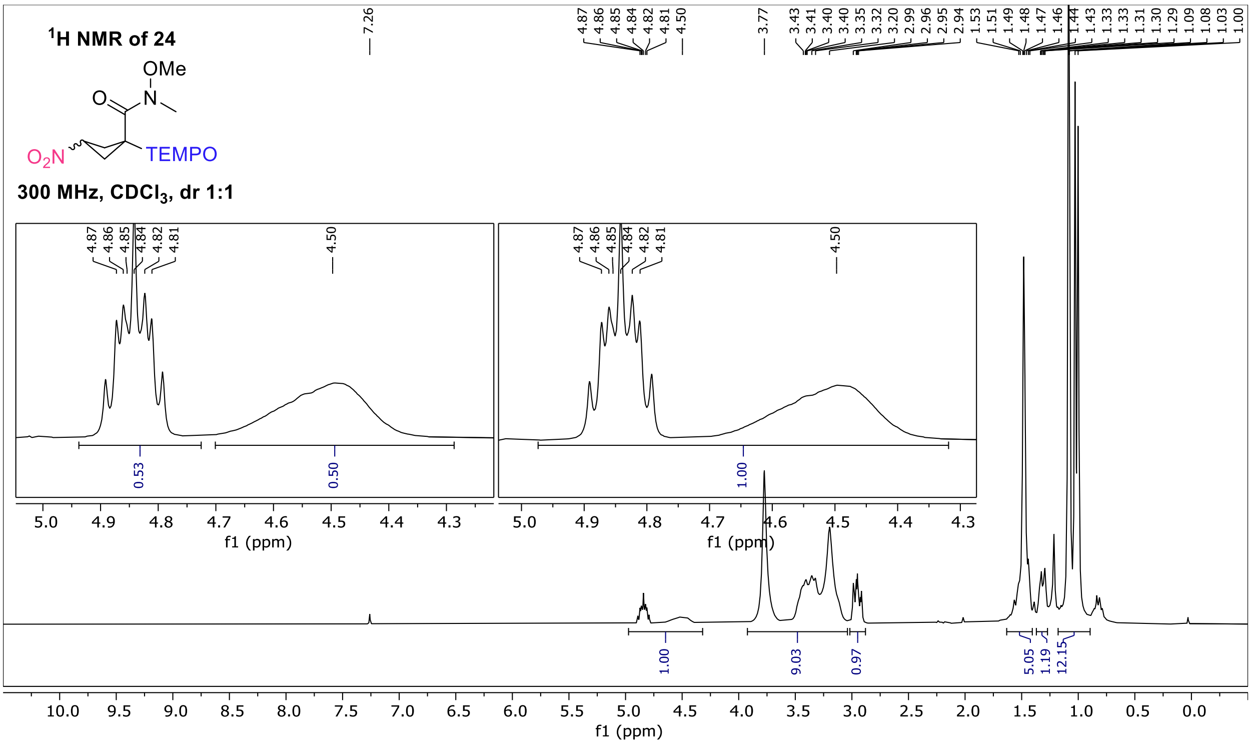

^{13}C NMR of 24

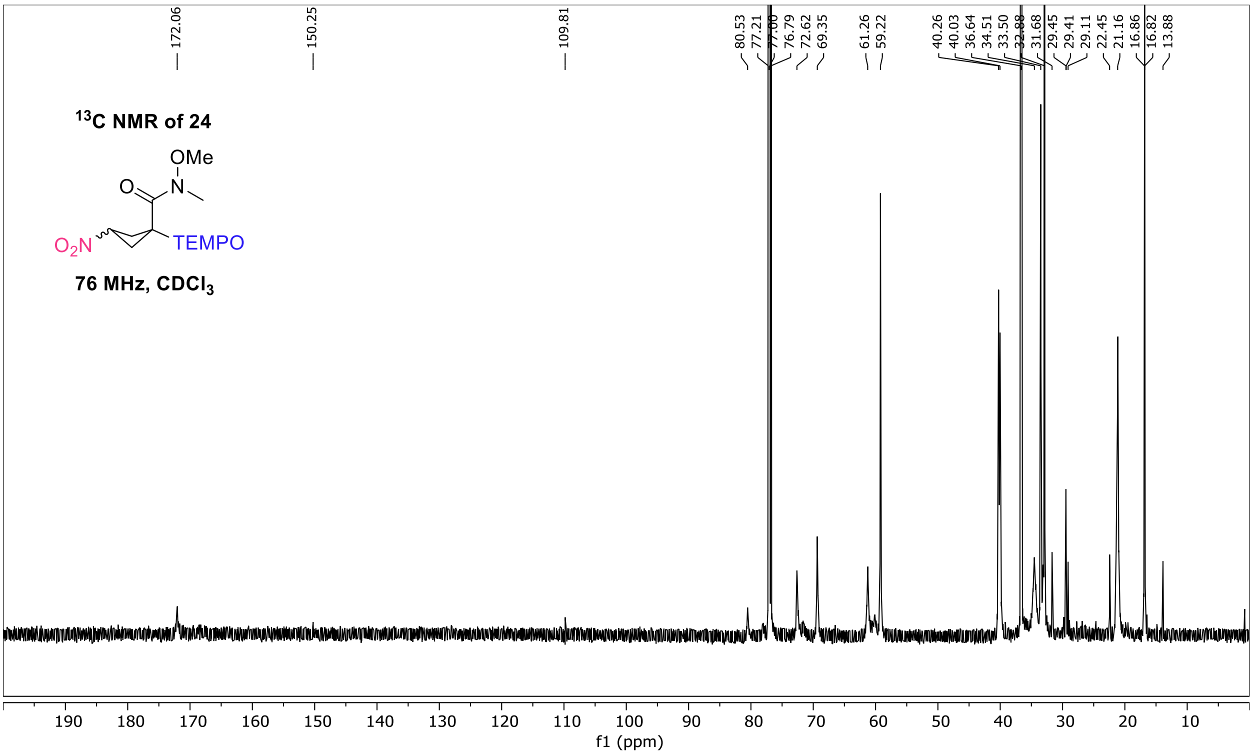

^{1}H NMR of 25 ($CDCl_3$)

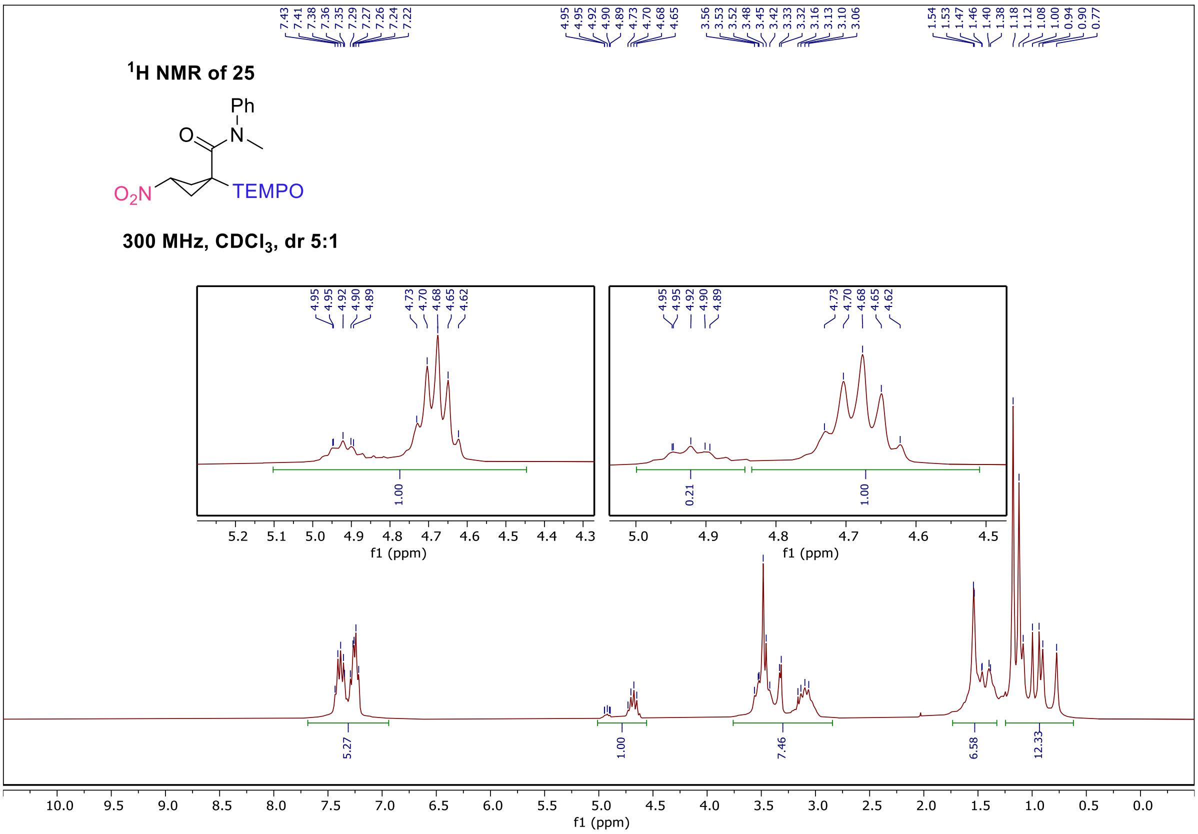

^{13}C NMR of 25 ($CDCl_3$)

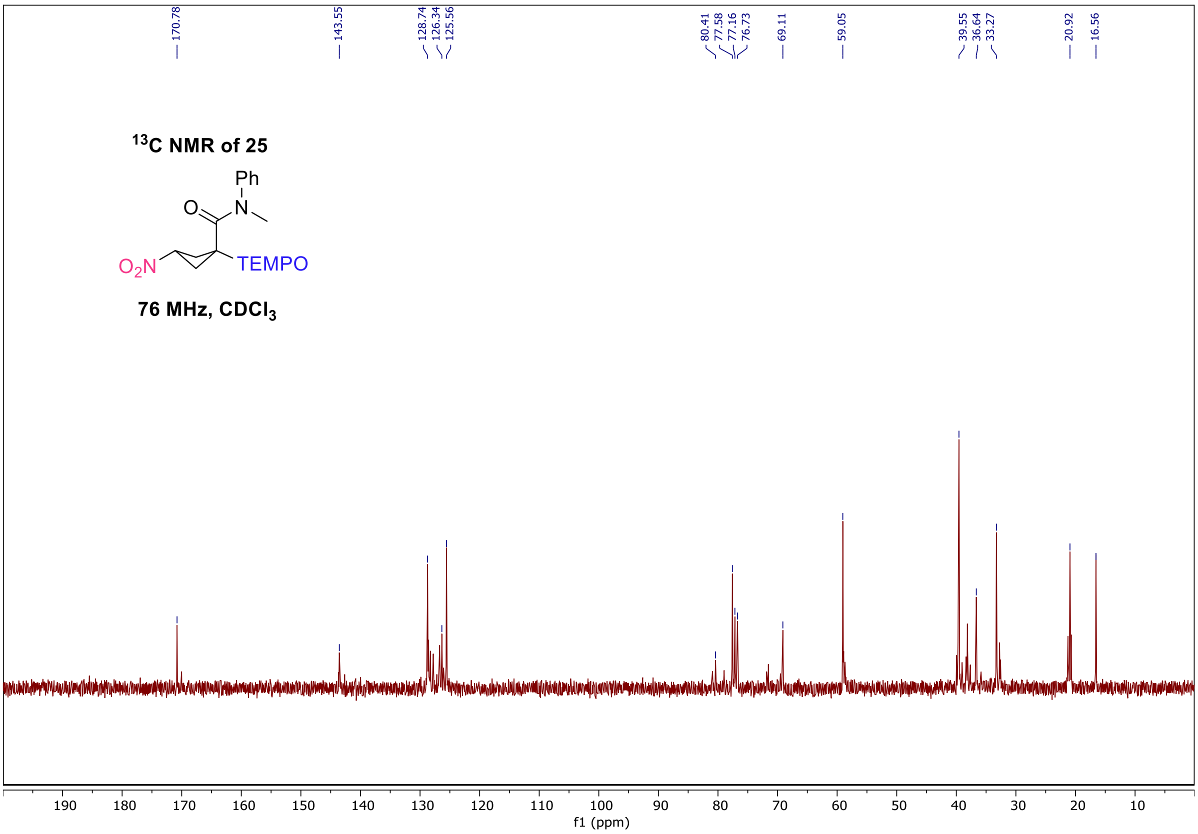

^{1}H NMR of 25 (DMSO-d_6)

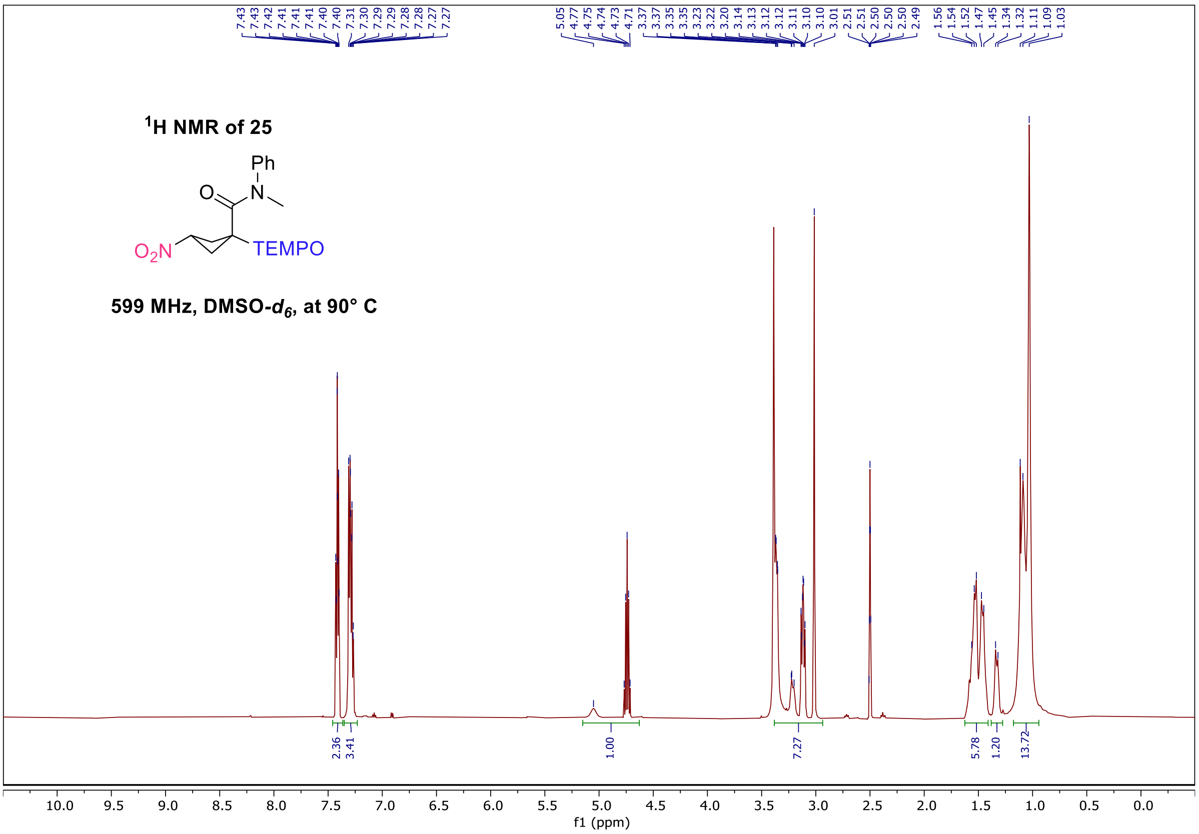

^{13}C NMR of 25 (DMSO-d_6)

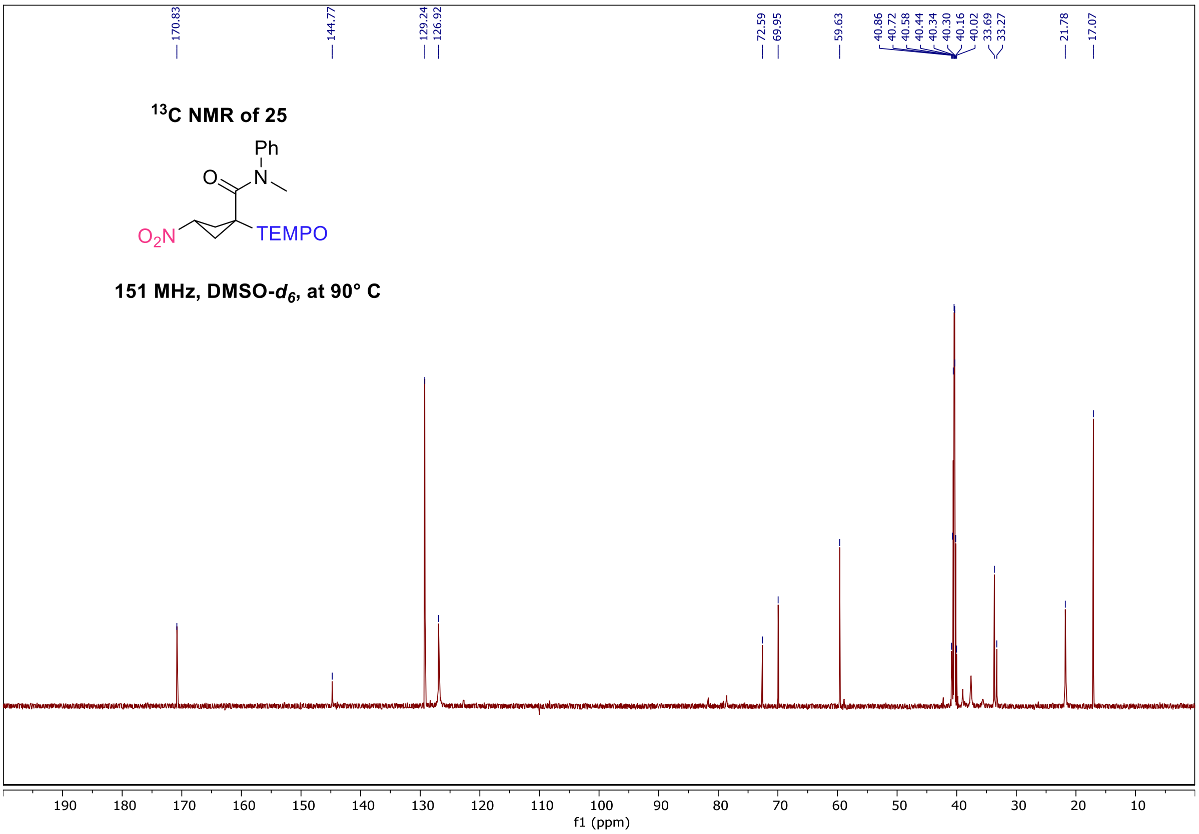

^{1}H NMR of 26

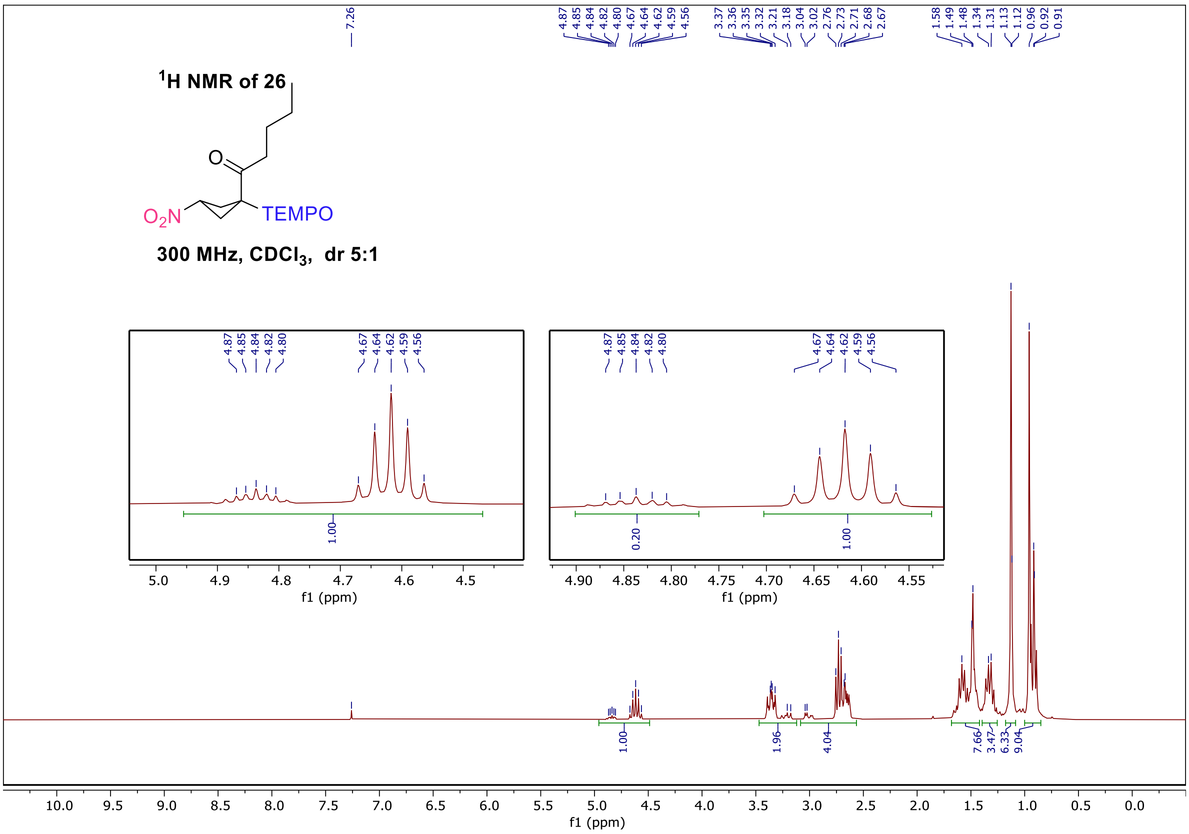

^{13}C NMR of 26

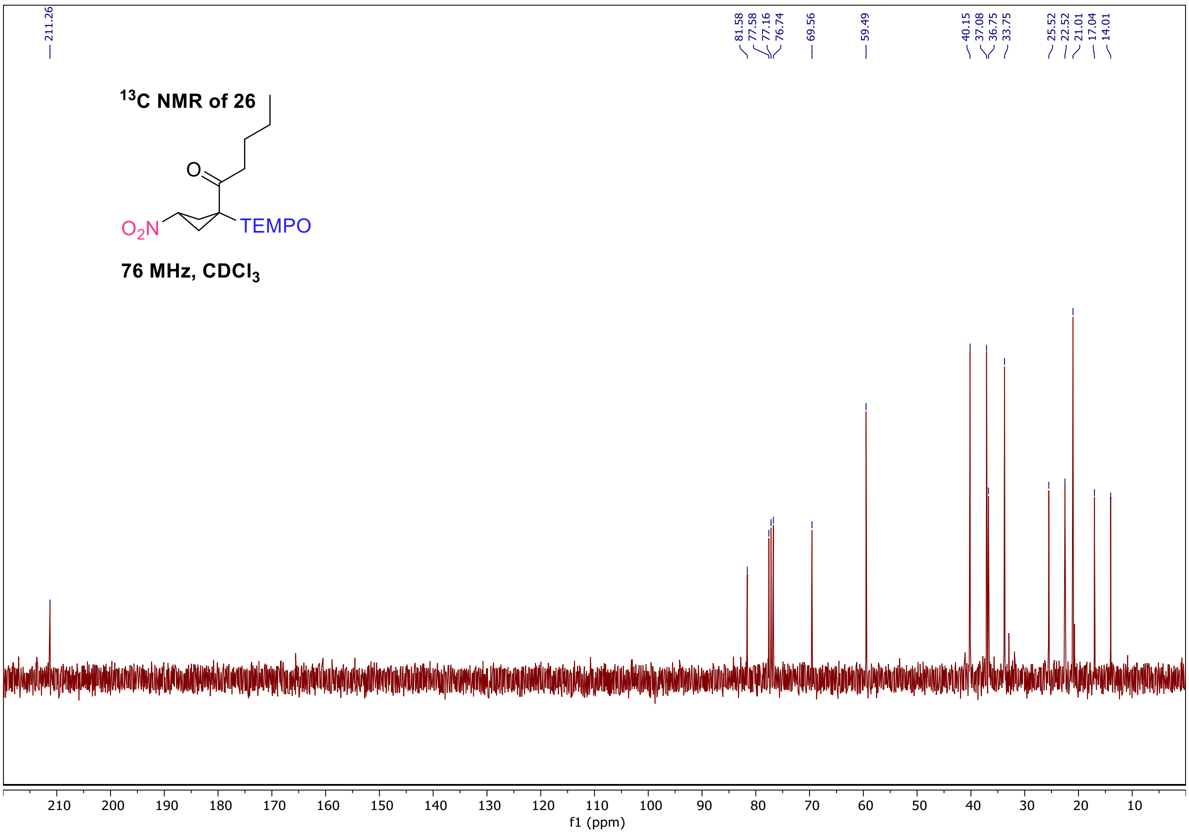

^{1}H NMR of 27 (CDCl$_3$, 300 MHz)

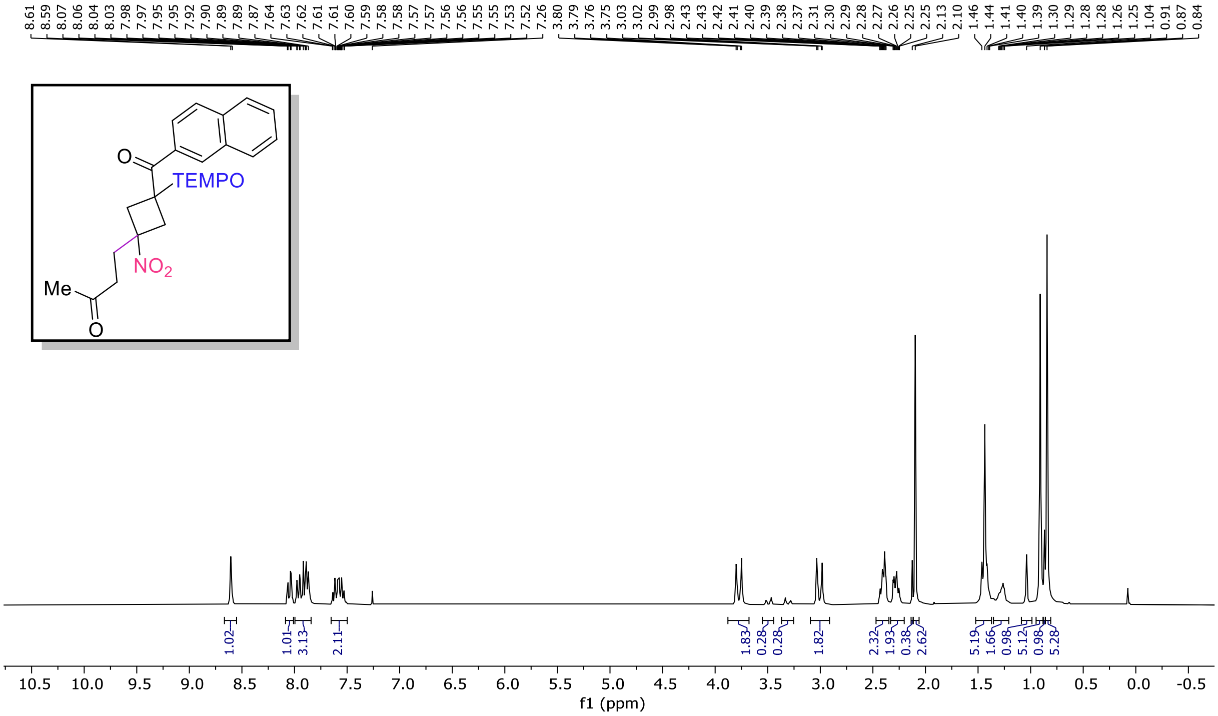

^{13}C NMR of 27 (CDCl$_3$, 101 MHz)

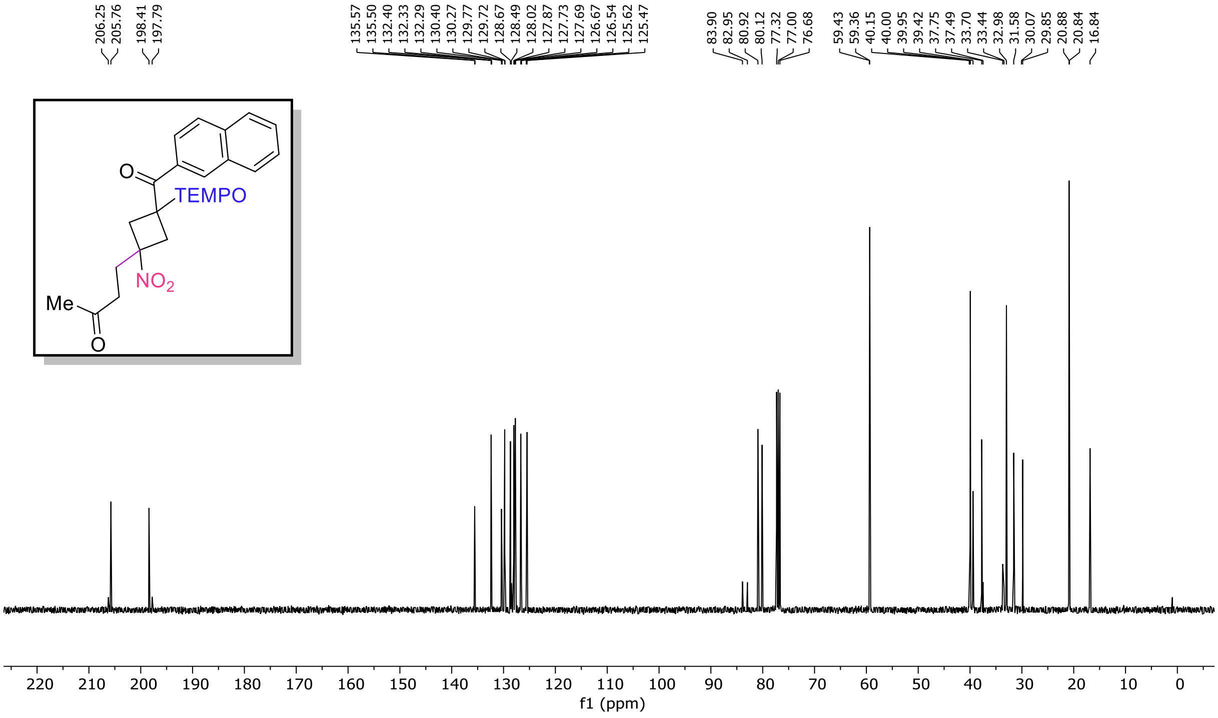

^{1}H NMR of 28 (CDCl3, 500 MHz)

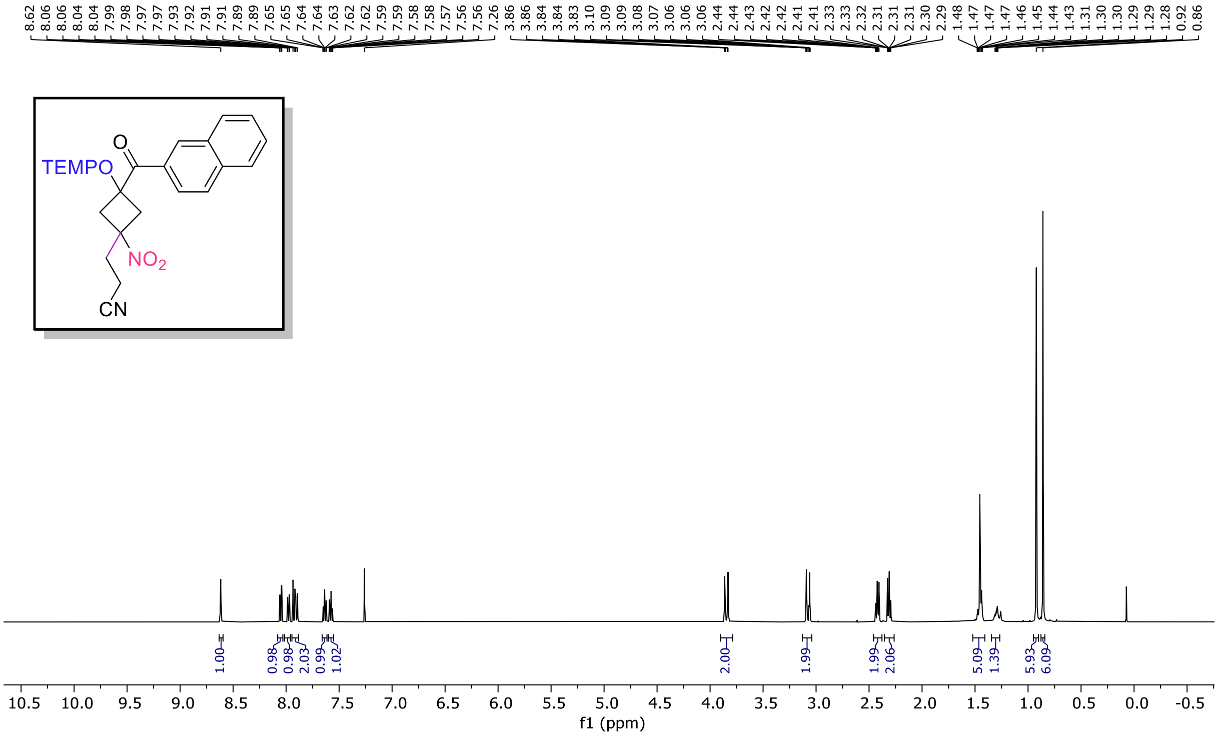

^{13}C NMR of 28 (CDCl3, 126 MHz)

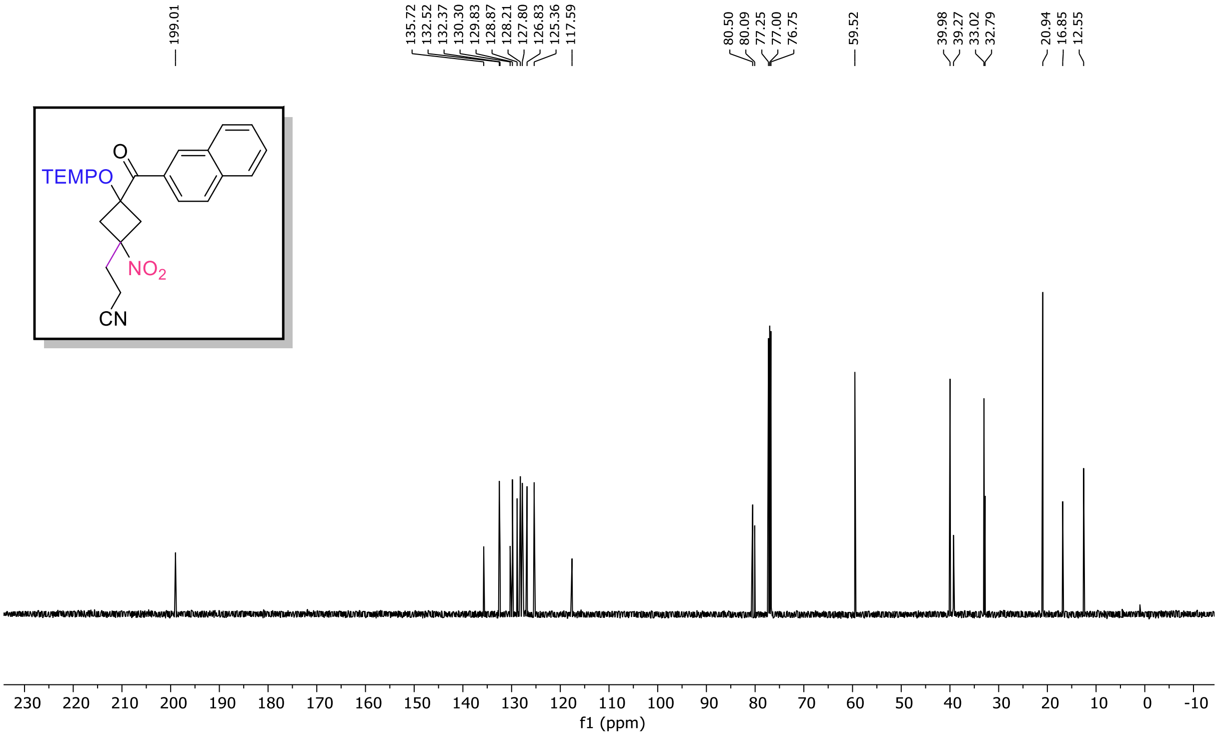

^{1}H NMR of 29a (CDCl3, 300 MHz)

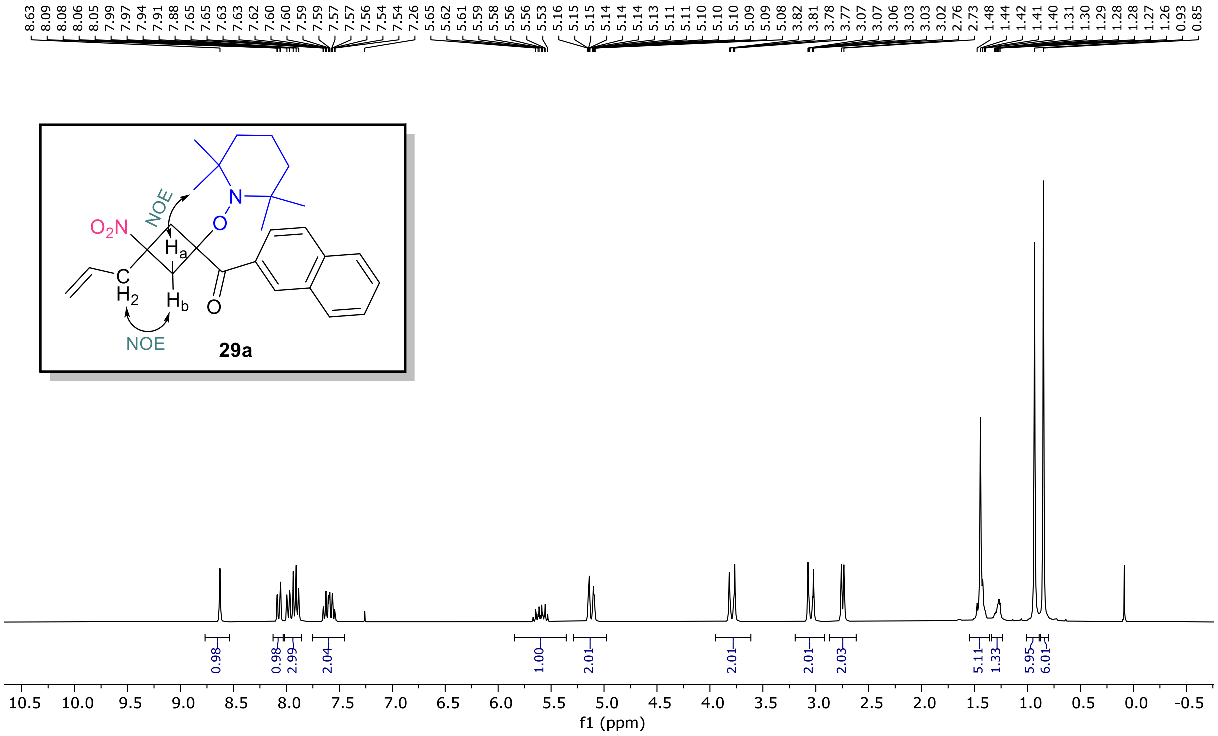

^{13}C NMR of 29a (CDCl3, 76 MHz)

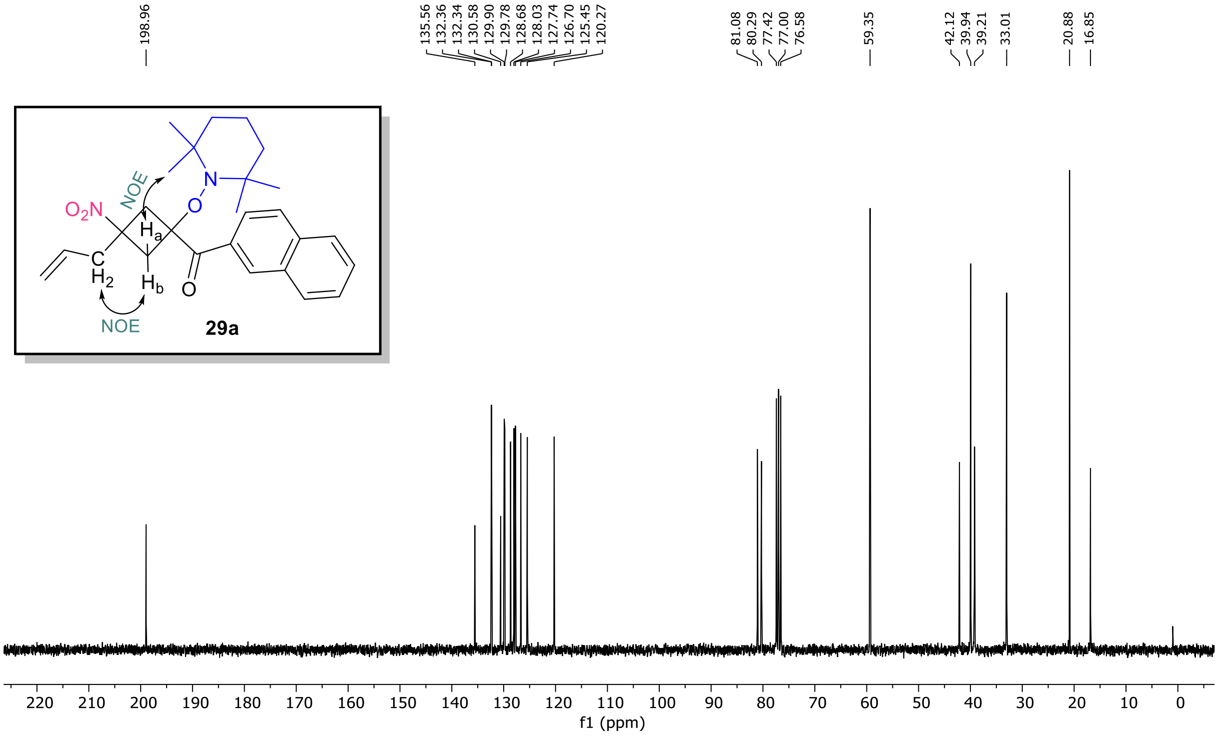

^{1}H NMR of 29b ($CDCl_3$, 300 MHz)

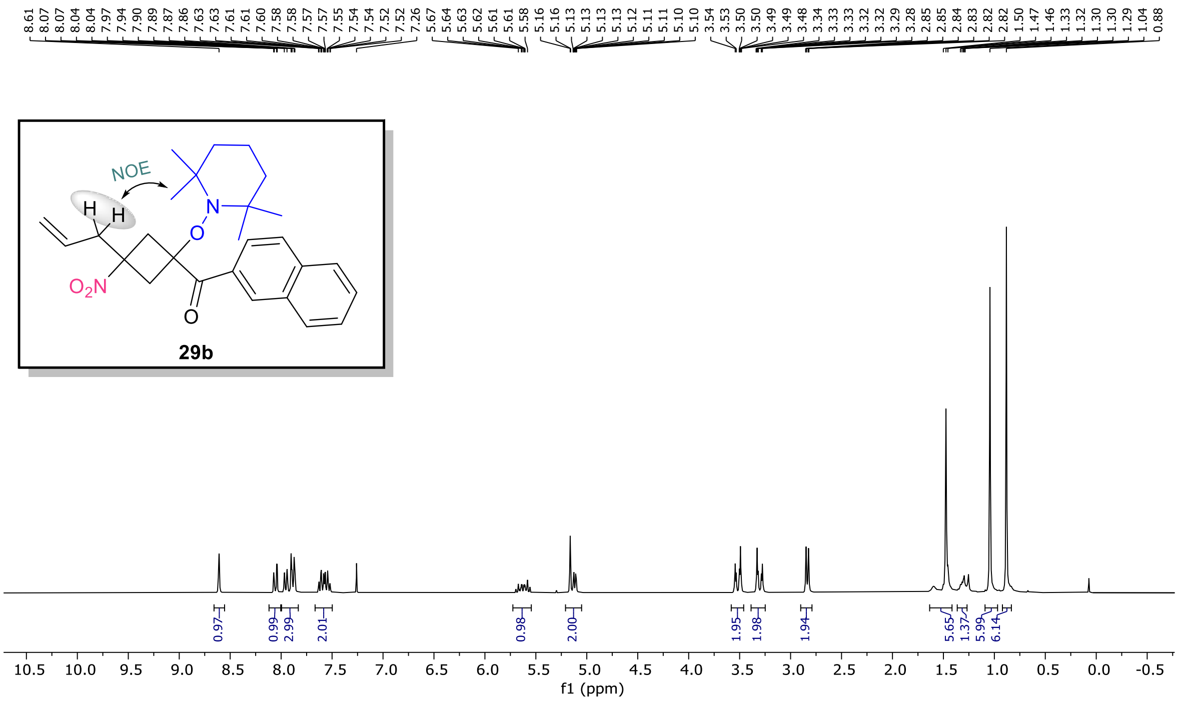

^{13}C NMR of 29b ($CDCl_3$, 76 MHz)

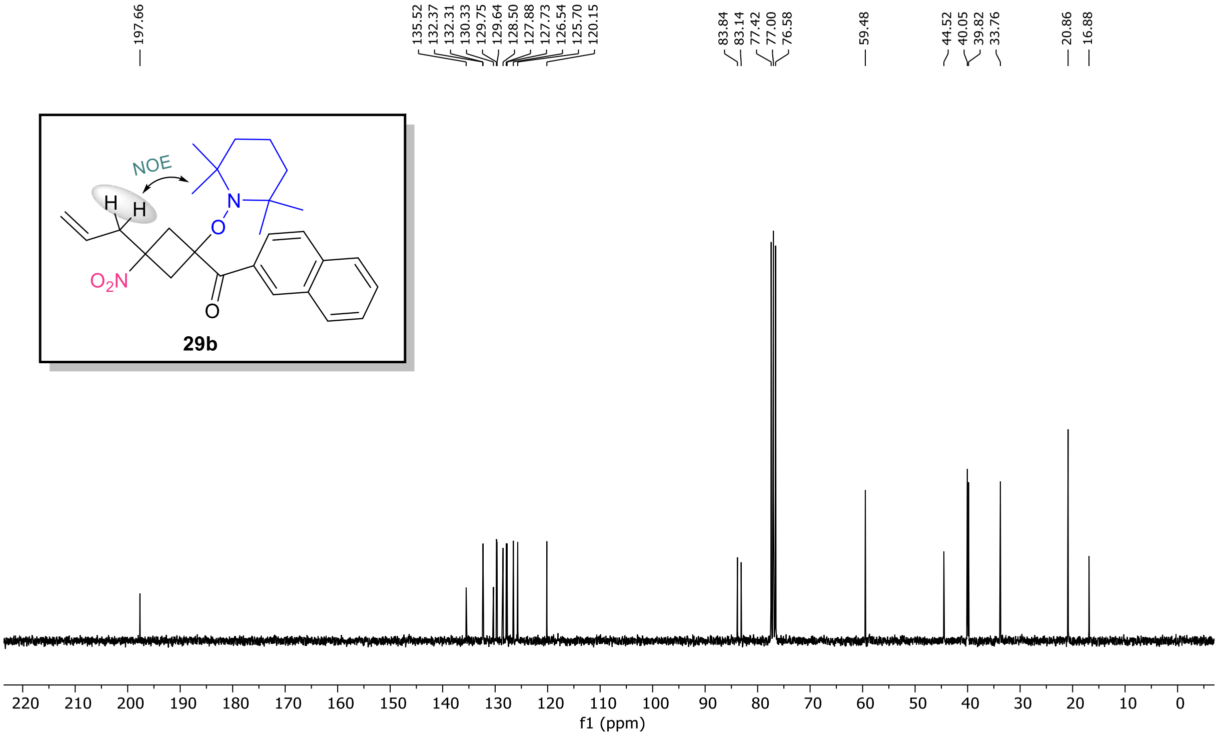

^{1}H NMR of 30a (CDCl$_3$, 300 MHz)

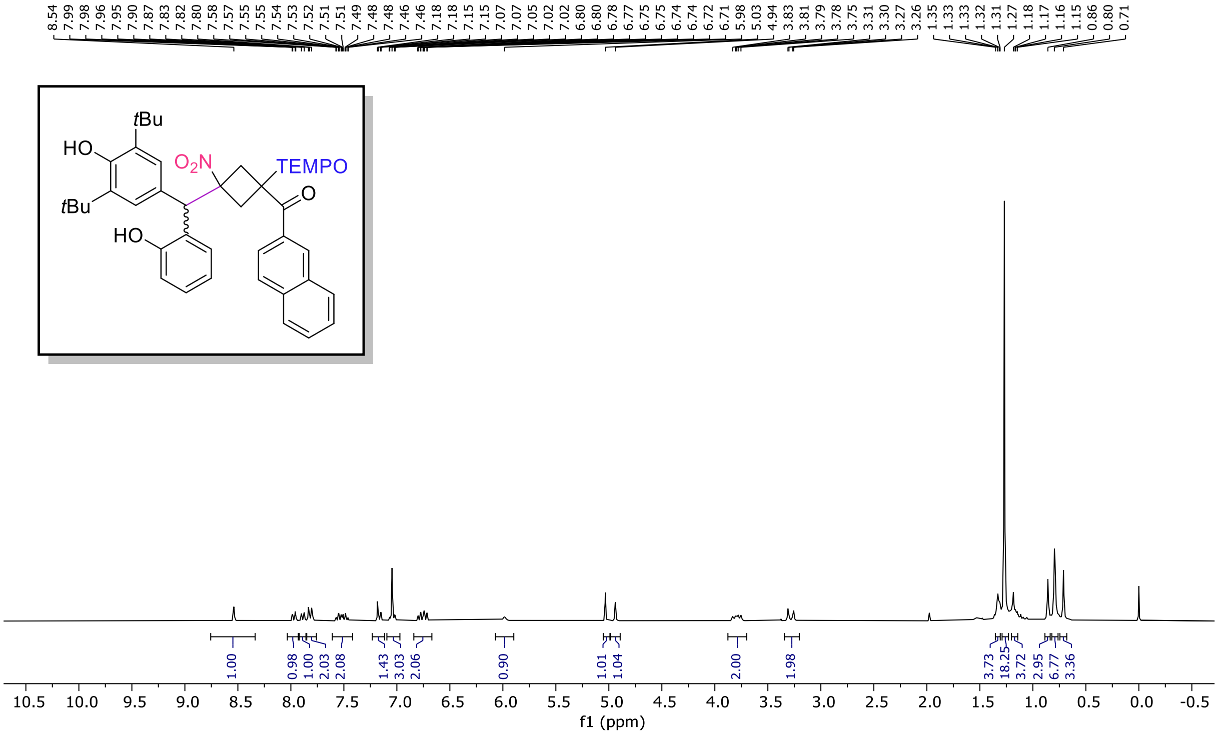

^{13}C NMR of 30a (CDCl$_3$, 76 MHz)

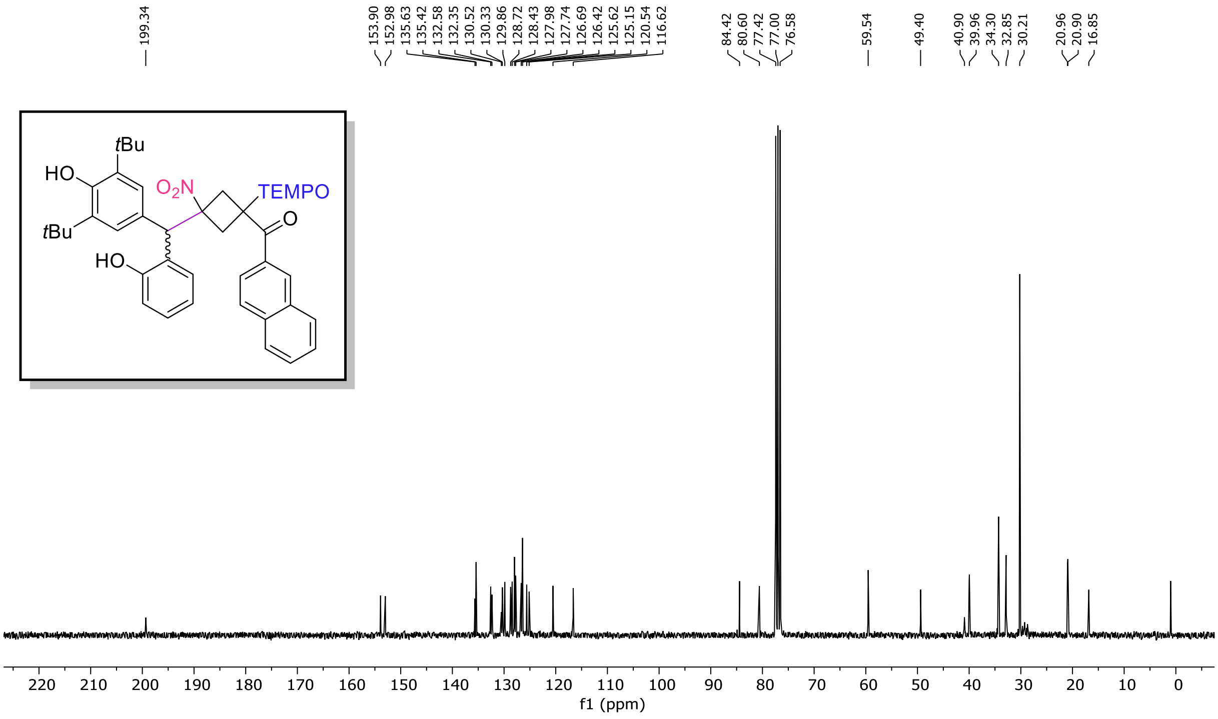

¹H NMR of 30b (CDCl3, 300 MHz)

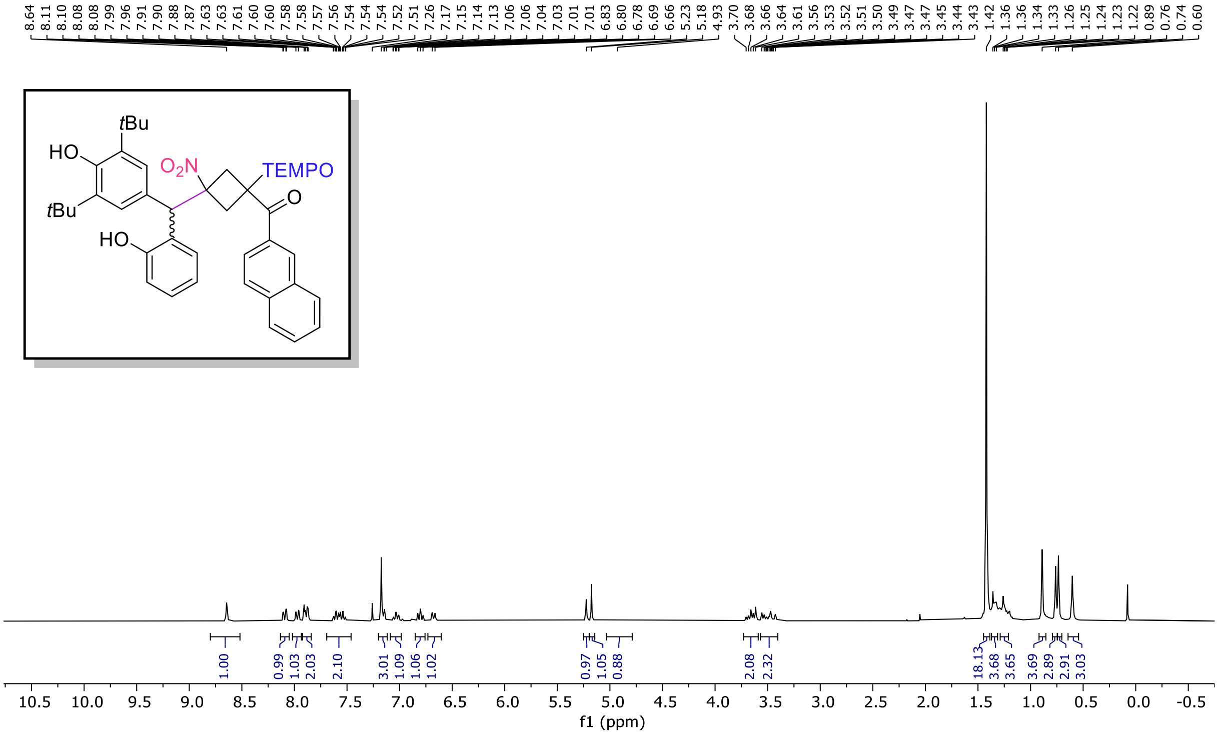

¹³C NMR of 30b (CDCl3, 76 MHz)

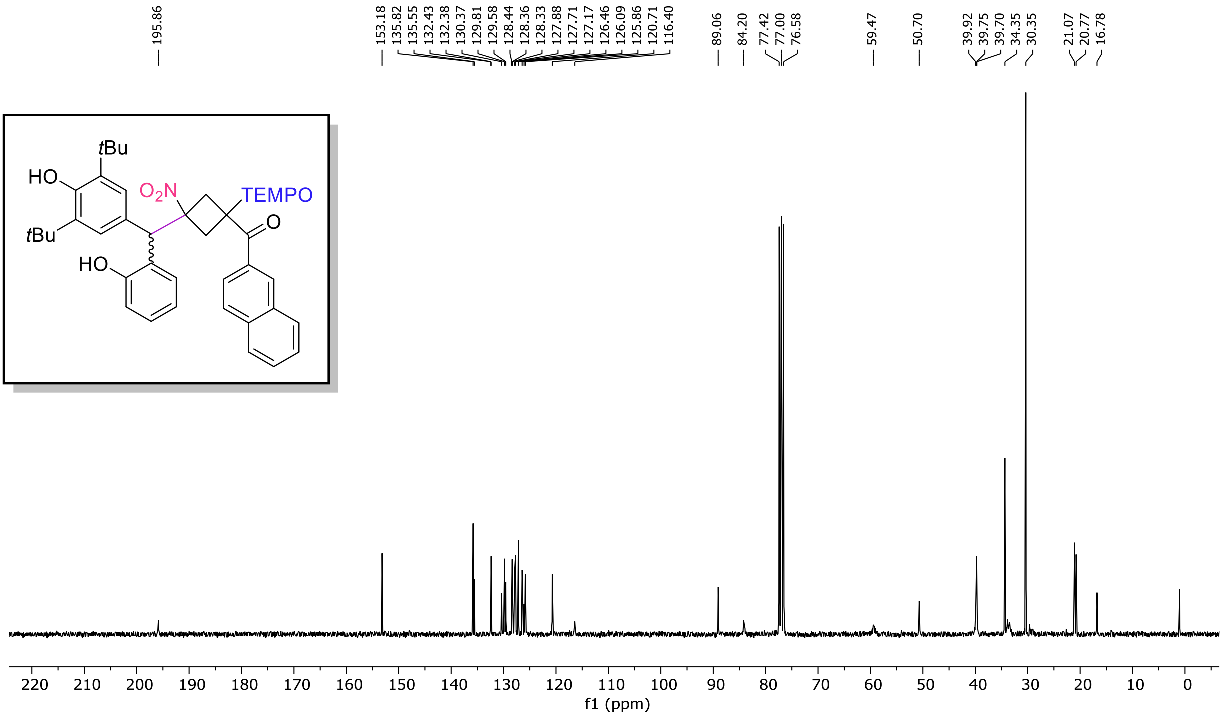

^{1}H NMR of 31 (CDCl$_3$, 300 MHz)

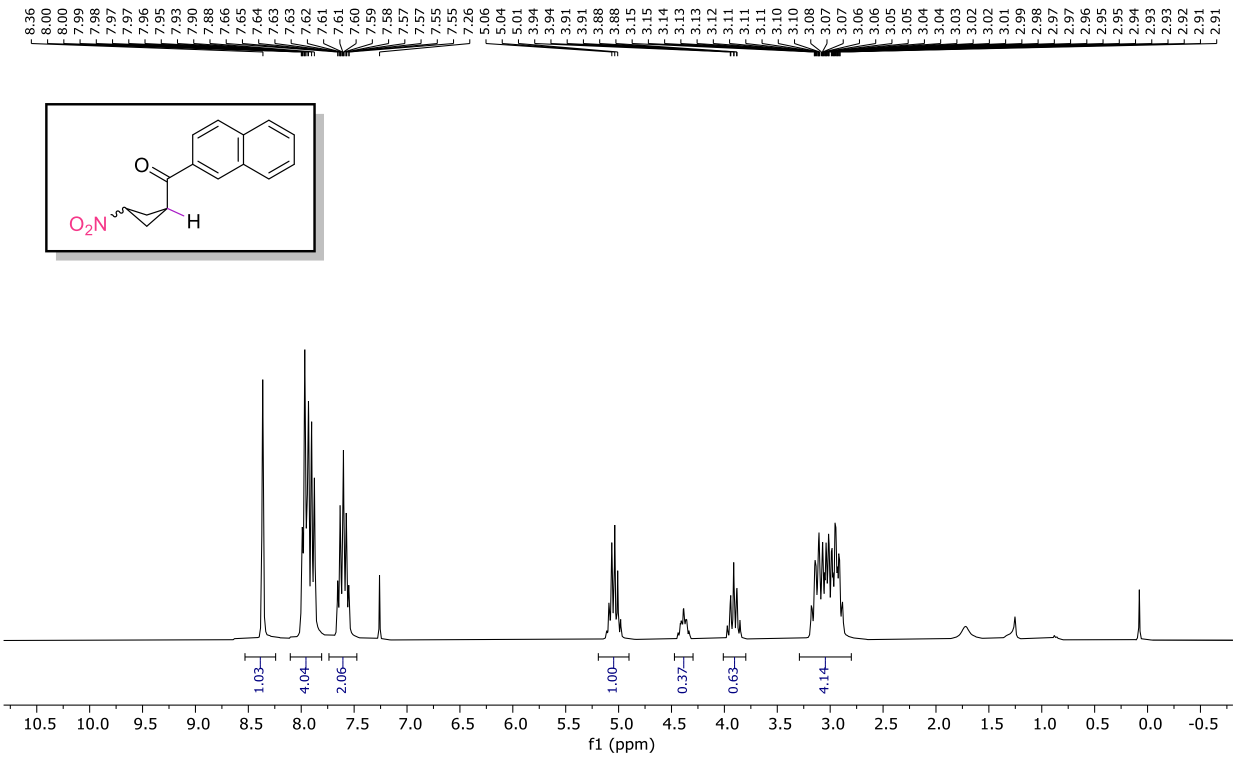

^{13}C NMR of 31 (CDCl$_3$, 76 MHz)

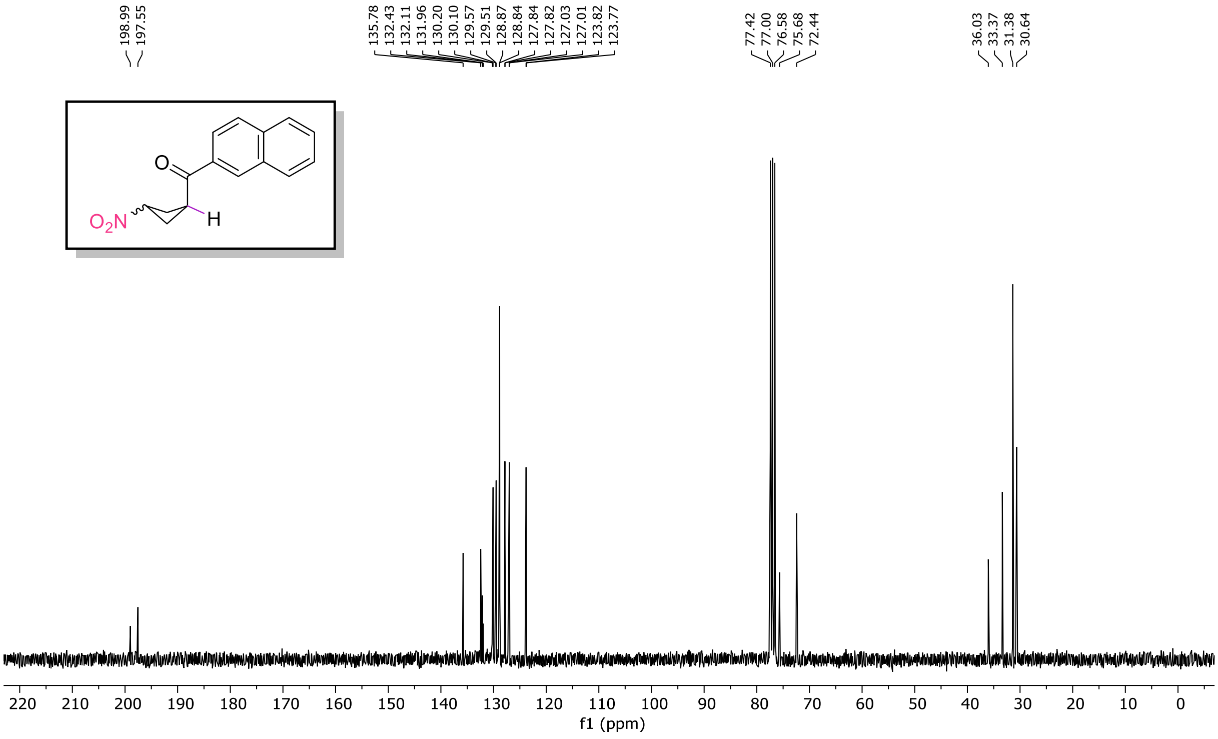

^{1}**H NMR of 32** ($CDCl_3$, 300 MHz)

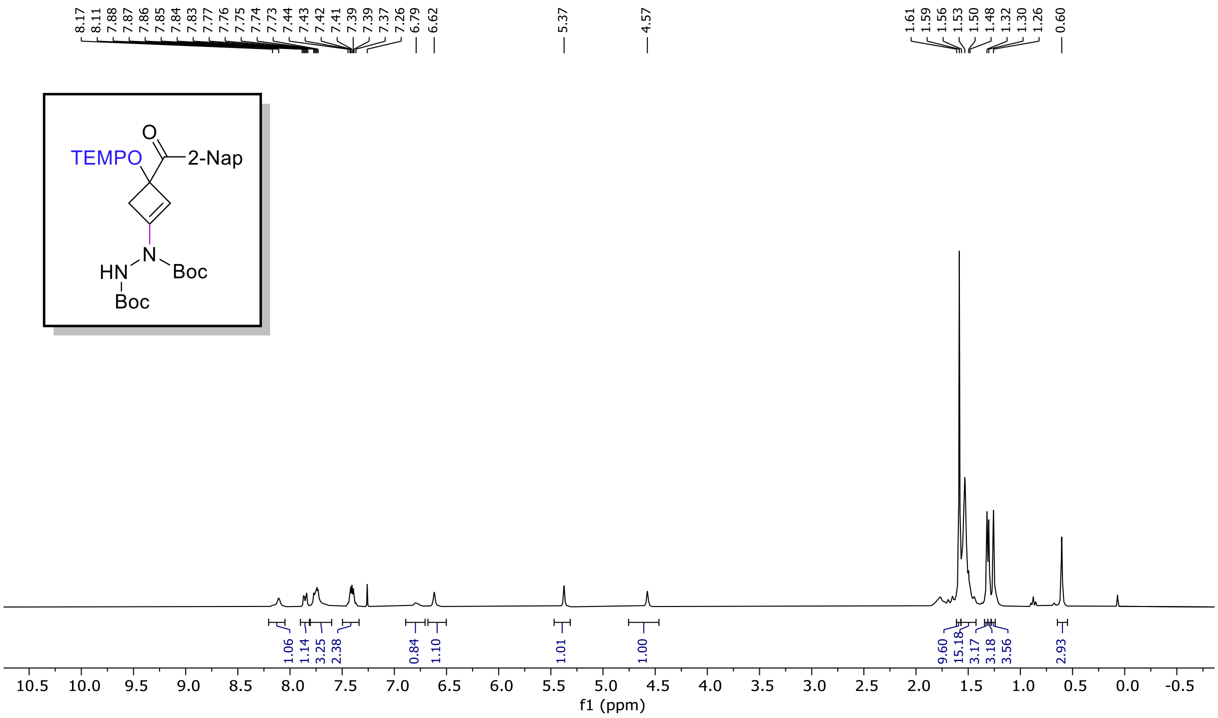

^{13}**C NMR of 32** ($CDCl_3$, 76 MHz)

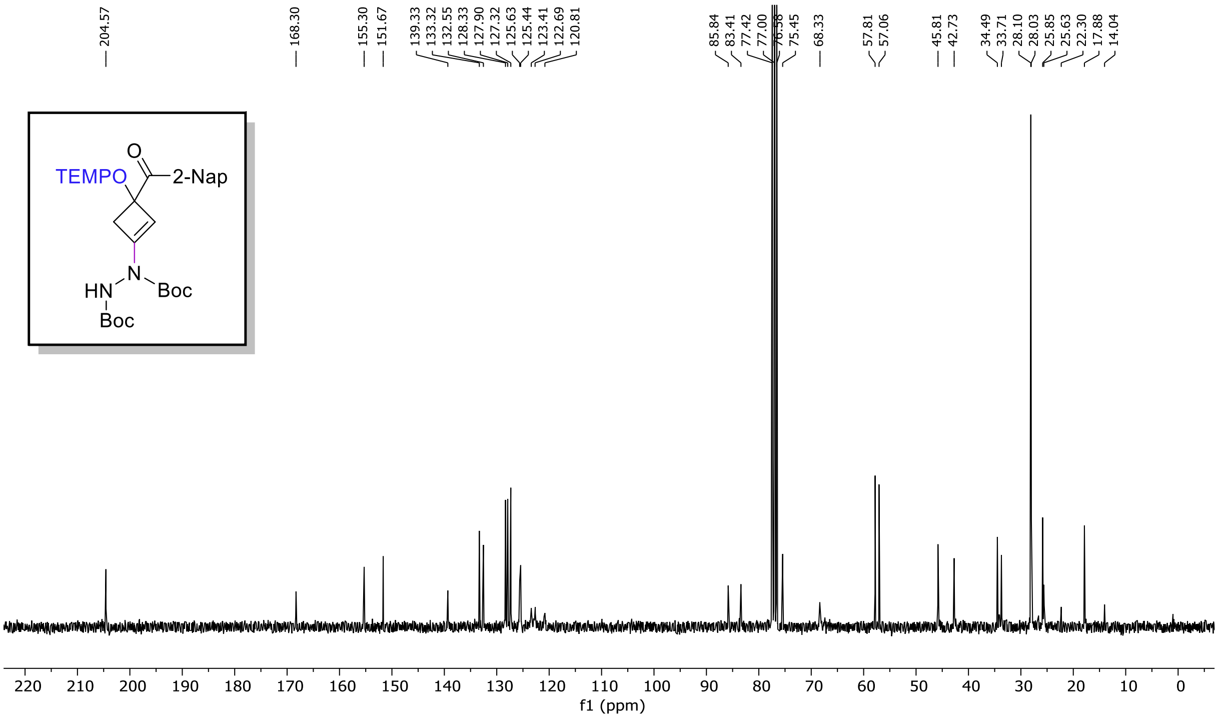

¹H NMR of S18 (CDCl$_3$, 400 MHz)

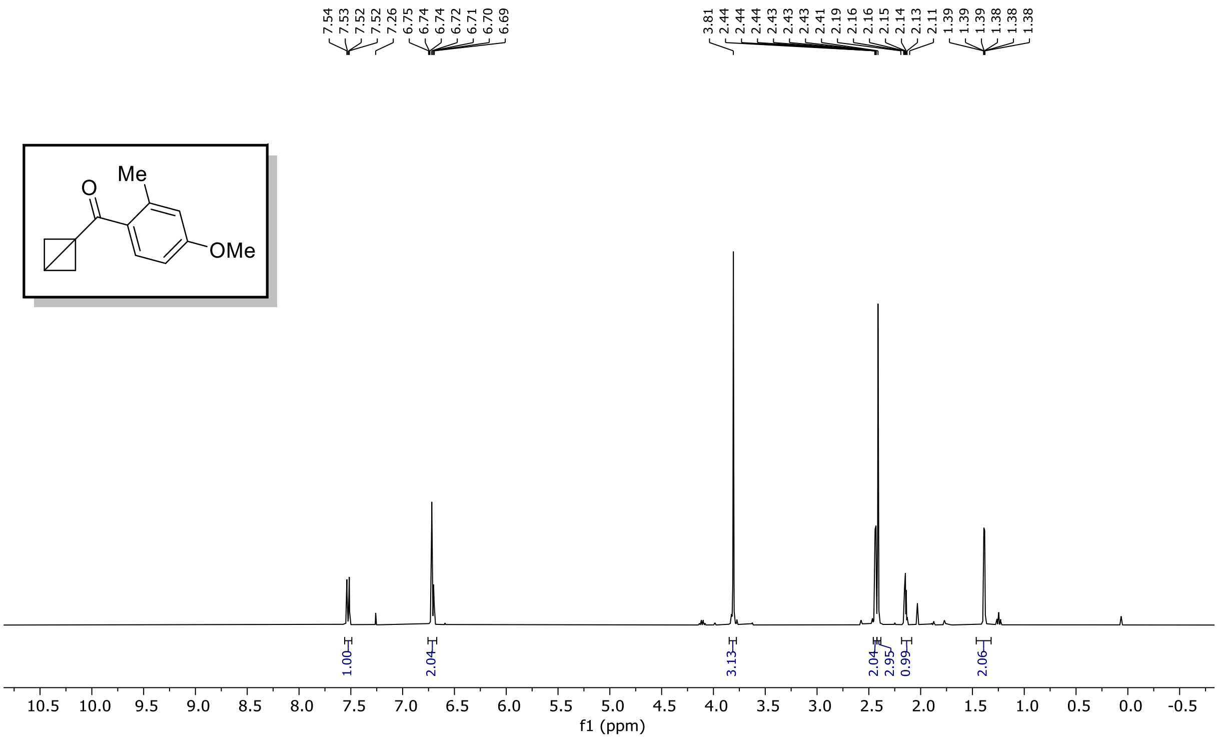

¹³C NMR of S18 (CDCl$_3$, 101 MHz)

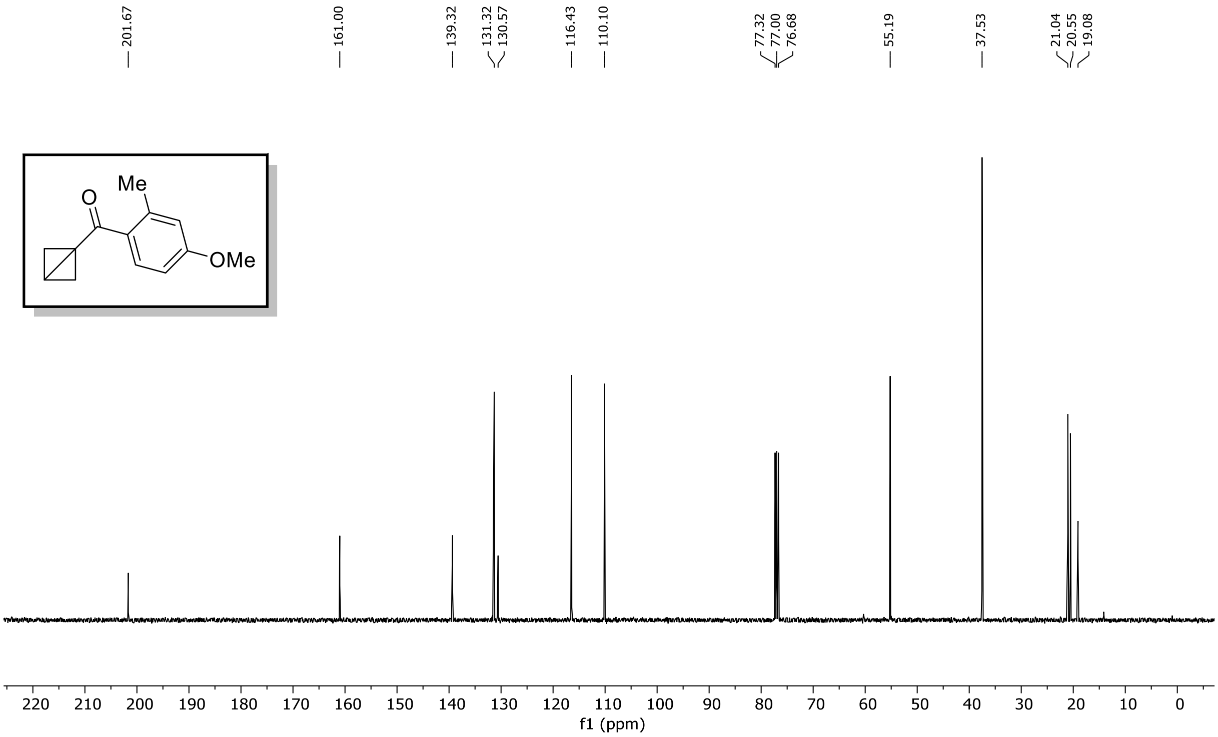

NOE of 29a

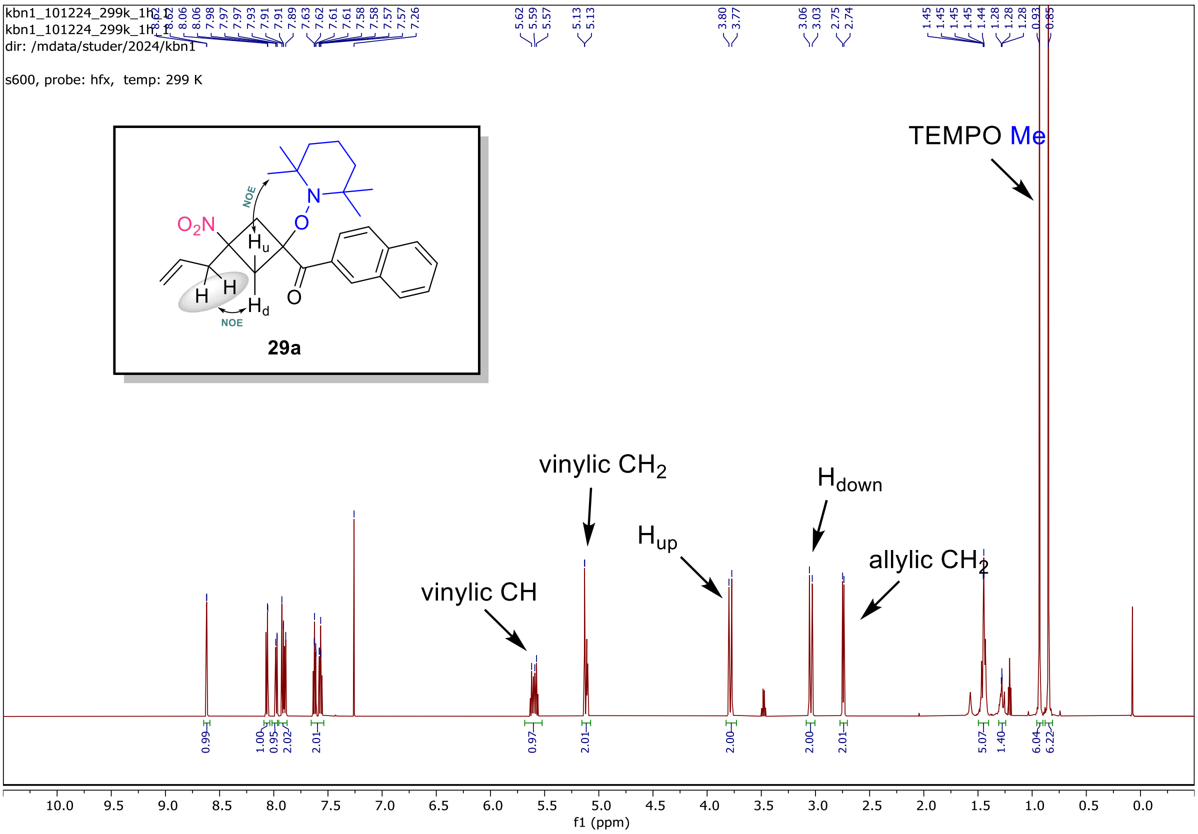
kbn1_101224_299k_1H_1
kbn1_101224_299k_1H_1
dir: /mdata/studer/2024/kbn1
s600, probe: hfx, temp: 299 K
29a
TEMPO Me
vinylic CH$_2$
H$_{up}$
H$_{down}$
allylic CH$_2$
vinylic CH
f1 (ppm)

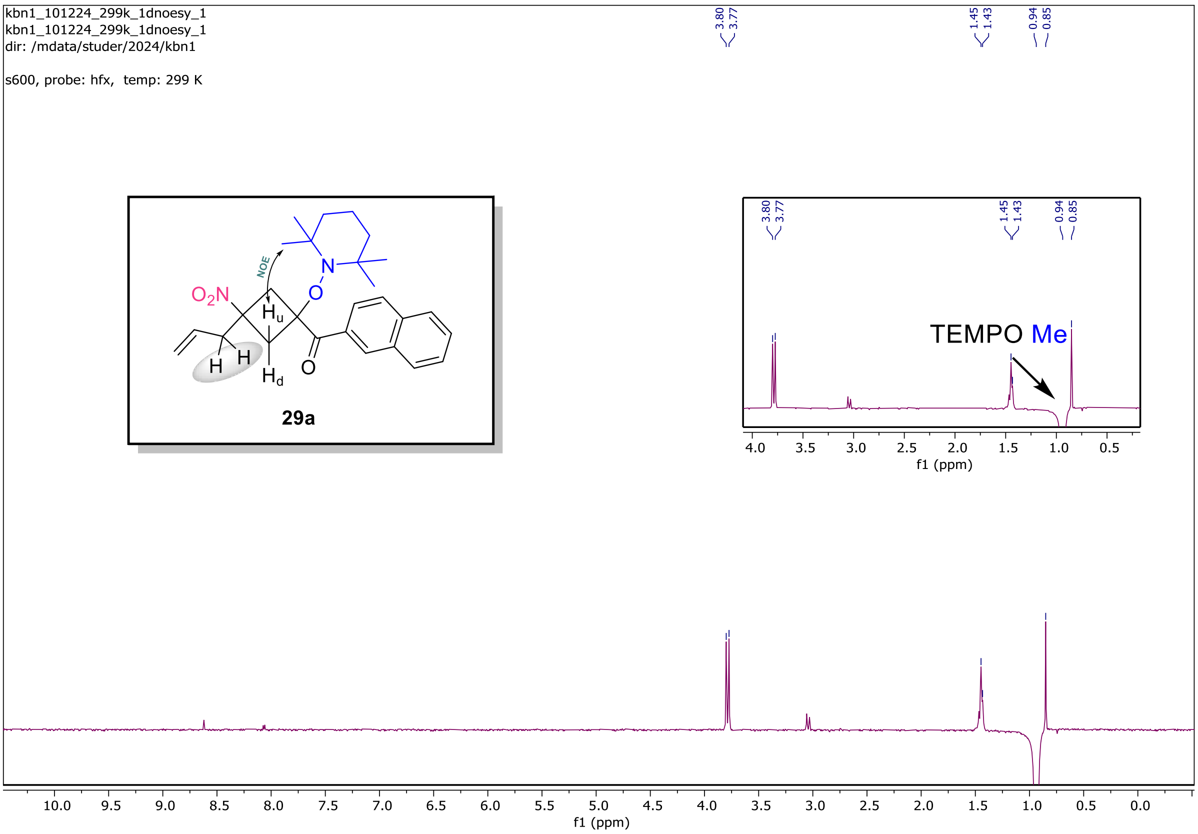
kbn1_101224_299k_1dnoesy_1
kbn1_101224_299k_1dnoesy_1
dir: /mdata/studer/2024/kbn1
s600, probe: hfx, temp: 299 K
29a
TEMPO Me
f1 (ppm)

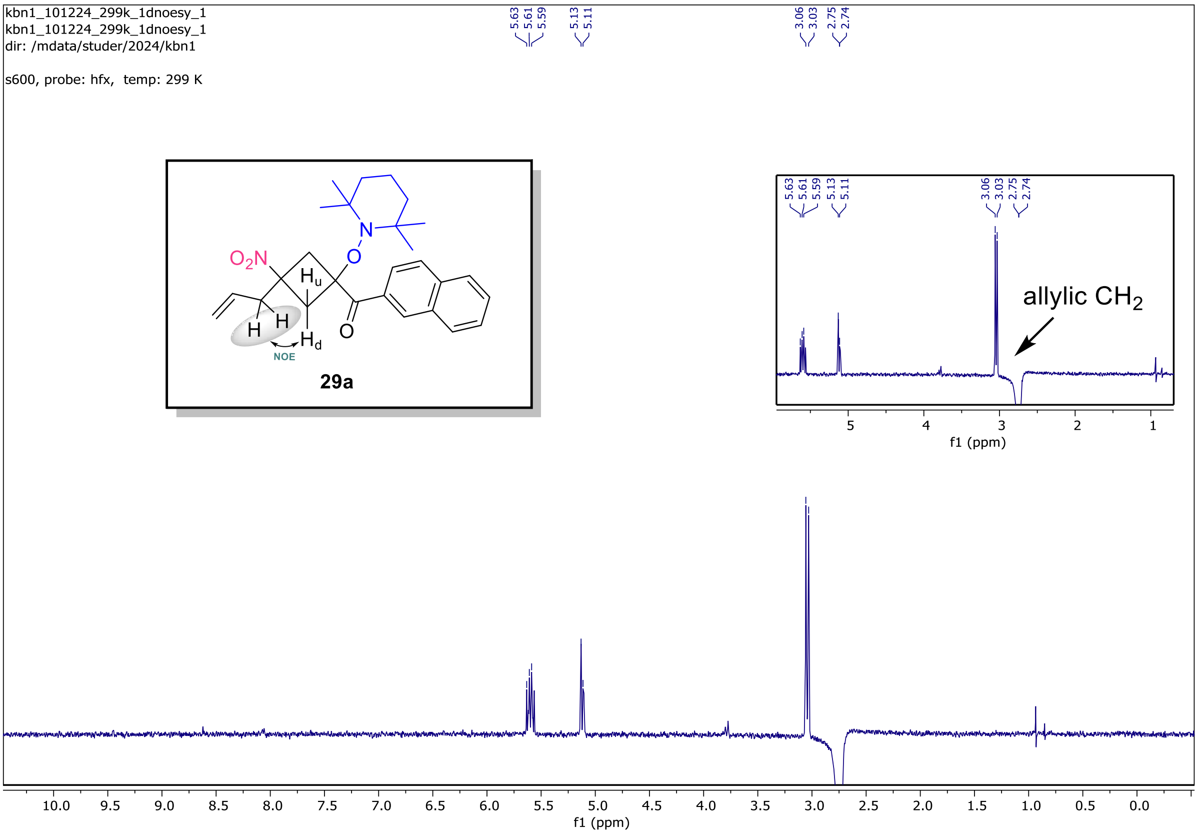
kbn1_101224_299k_1dnoesy_1
kbn1_101224_299k_1dnoesy_1
dir: /mdata/studer/2024/kbn1
s600, probe: hfx, temp: 299 K
5.63
5.61
5.59
5.13
5.11
3.06
3.03
2.75
2.74
O2N
Hu
O
N
H
H
Hd
NOE
O
29a
allylic CH2
f1 (ppm)
f1 (ppm)

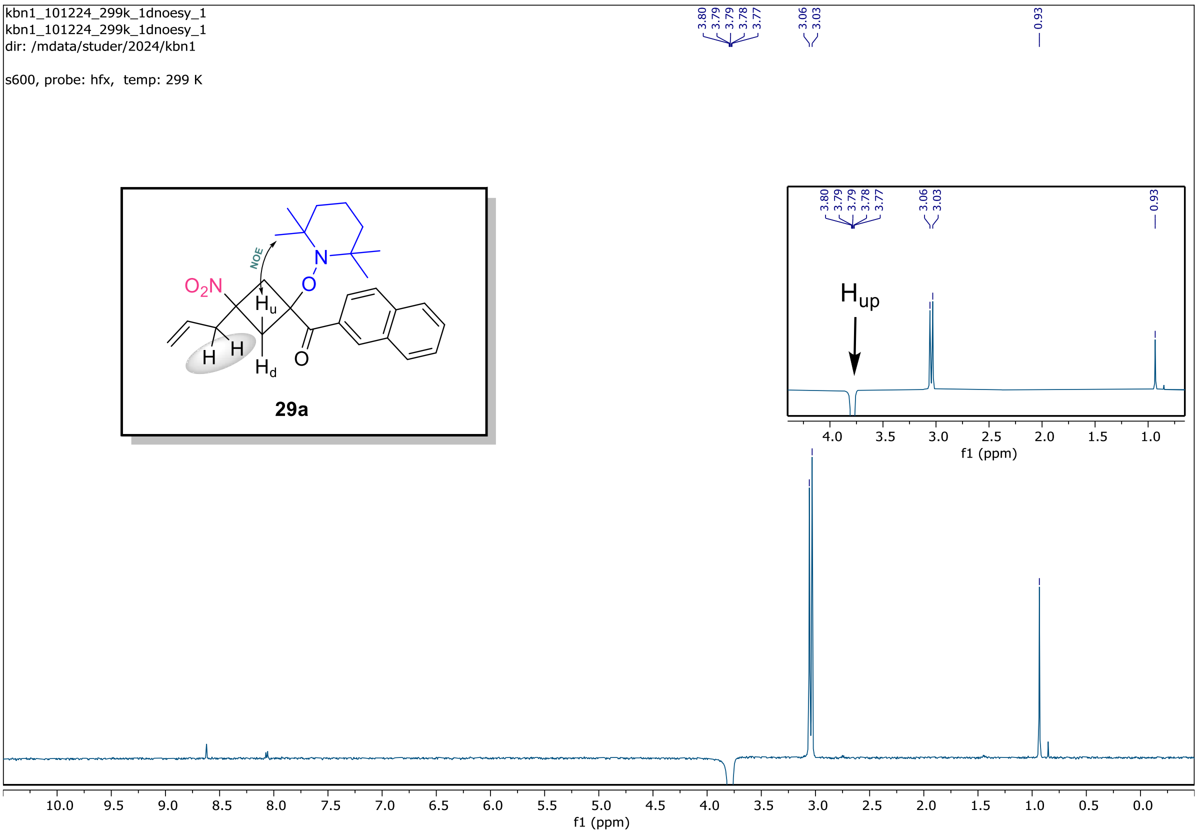
kbn1_101224_299k_1dnoesy_1
kbn1_101224_299k_1dnoesy_1
dir: /mdata/studer/2024/kbn1
s600, probe: hfx, temp: 299 K
3.80
3.79
3.79
3.78
3.77
3.06
3.03
0.93
NOE
O2N
N
O
Hu
H
H
Hd
O
29a
Hup
f1 (ppm)
f1 (ppm)

NOE of 29b

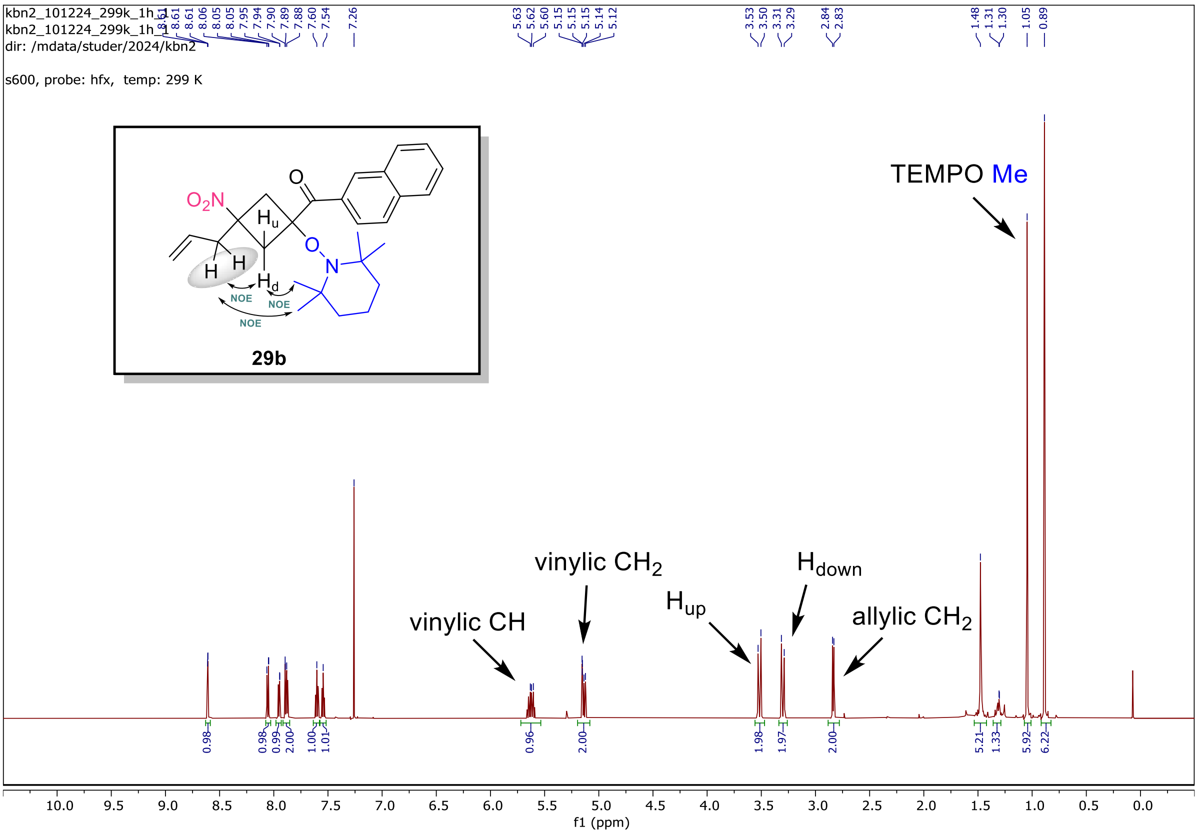
kbn2_101224_299k_1h_1
kbn2_101224_299k_1h_1
dir: /mdata/studer/2024/kbn2
s600, probe: hfx, temp: 299 K
O2N
Hu
Hd
O
N
NOE
29b
TEMPO Me
vinylic CH2
vinylic CH
Hup
Hdown
allylic CH2
f1 (ppm)

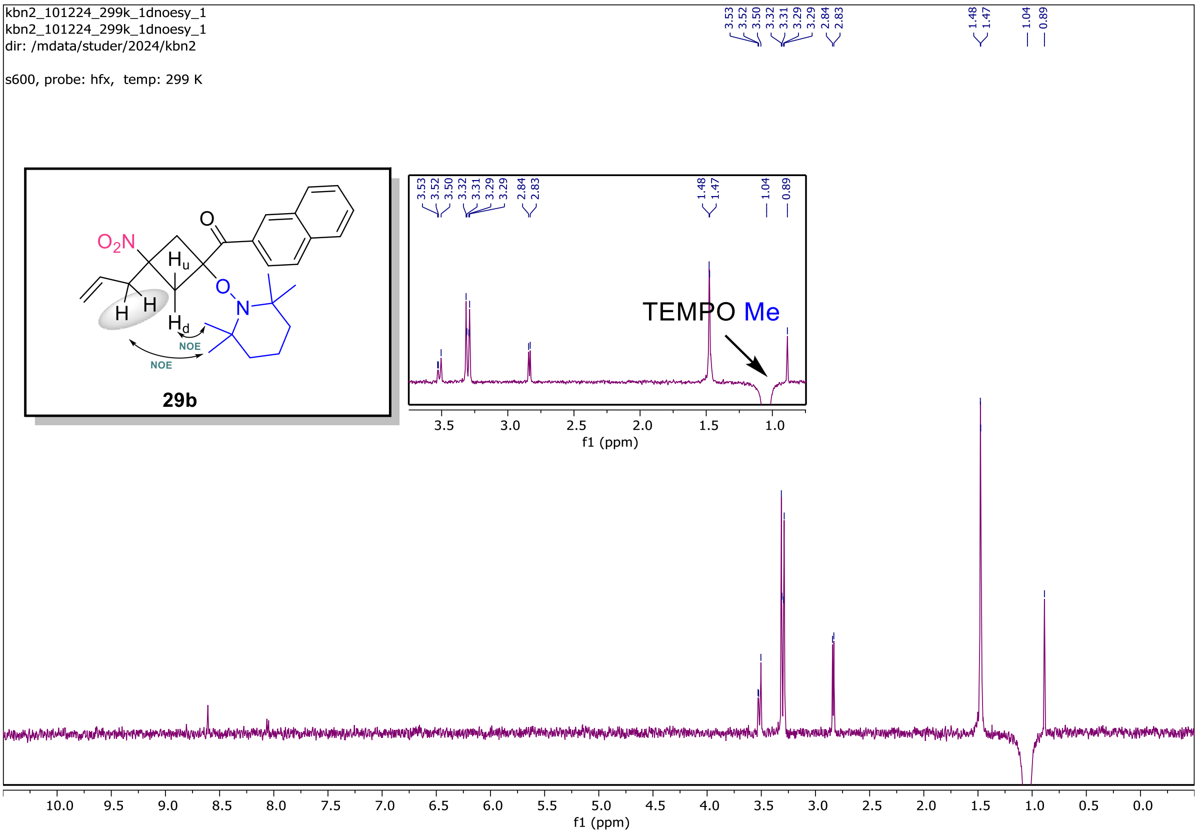
kbn2_101224_299k_1dnoesy_1
kbn2_101224_299k_1dnoesy_1
dir: /mdata/studer/2024/kbn2
s600, probe: hfx, temp: 299 K
O2N
Hu
Hd
NOE
29b
TEMPO Me
f1 (ppm)

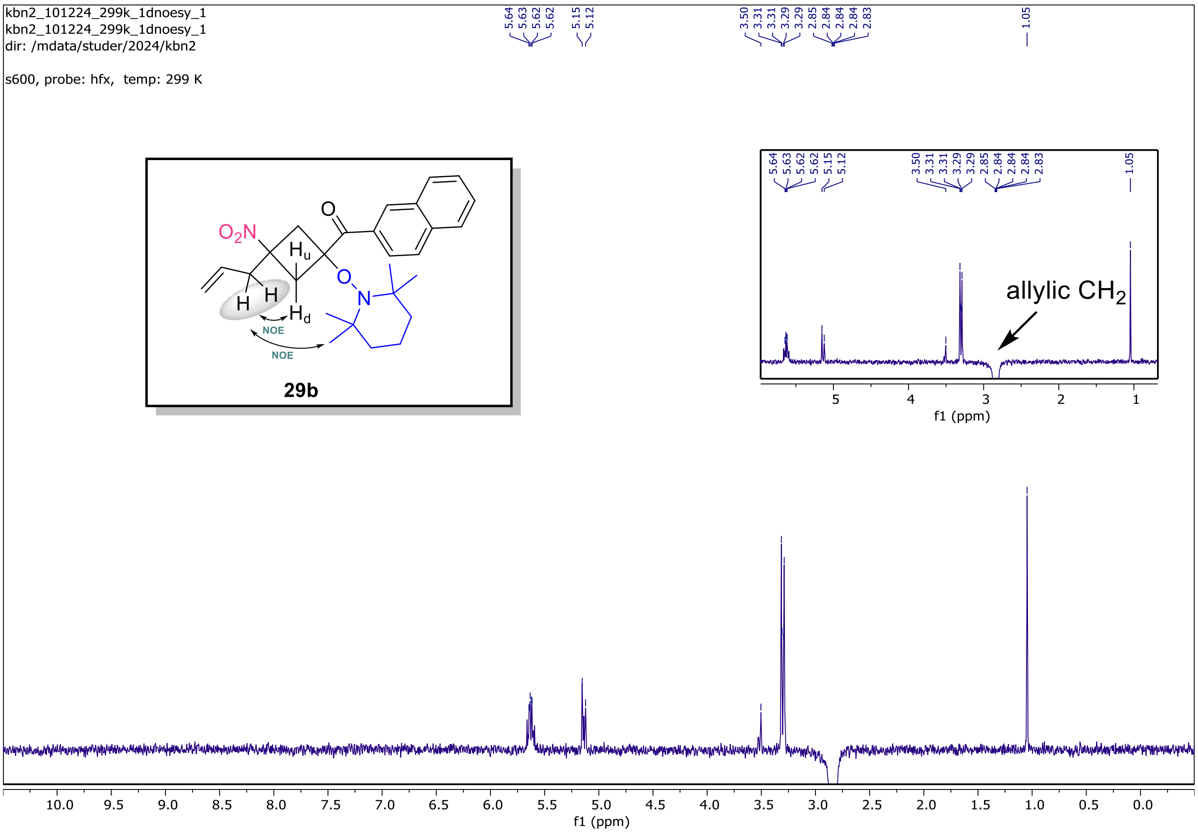
kbn2_101224_299k_1dnoesy_1
kbn2_101224_299k_1dnoesy_1
dir: /mdata/studer/2024/kbn2
s600, probe: hfx, temp: 299 K
5.64 5.63 5.62 5.62 5.15 5.12 3.50 3.31 3.31 3.29 3.29 2.85 2.84 2.84 2.84 2.83 1.05
NOE
NOE
29b
allylic CH2
f1 (ppm)

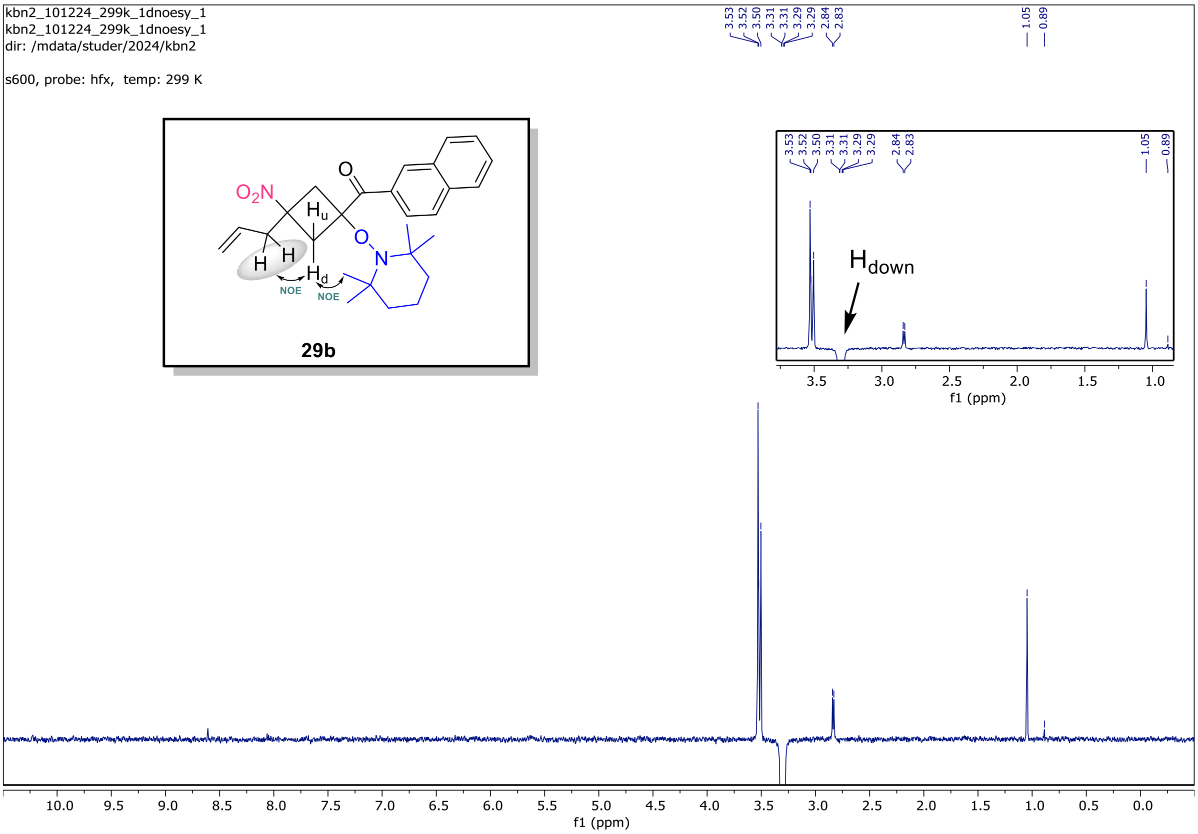
kbn2_101224_299k_1dnoesy_1
kbn2_101224_299k_1dnoesy_1
dir: /mdata/studer/2024/kbn2
s600, probe: hfx, temp: 299 K
3.53 3.52 3.50 3.31 3.31 3.29 3.29 2.84 2.83 1.05 0.89
NOE
NOE
29b
Hdown
f1 (ppm)
